# Chromatin stability safeguards mitochondrial homeostasis and restrains TORC1 signaling

**DOI:** 10.1371/journal.pgen.1012330

**Published:** 2026-10-08

**Authors:** Vinoth Sigamani, Mohd Yousuf, Daniel L. Johnson, Brian D. Strahl, R. Nicholas Laribee

**Affiliations:** 1 Department of Pathology and Laboratory Medicine, College of Medicine and the Center for Cancer Research, University of Tennessee Health Science Center, Memphis, Tennessee, United States of America; 2 Molecular Bioinformatics Core and the University of Tennessee Health Science Center Office of Research, University of Tennessee Health Science Center, Memphis, Tennessee, United States of America; 3 Department of Biochemistry and Biophysics, and the Lineberger Comprehensive Cancer Center, University of North Carolina at Chapel Hill, Chapel Hill, North Carolina, United States of America; Institute of Functional Epigenetics, GERMANY

## Abstract

Chromatin is dynamically regulated in response to nutrient flux to promote the transcriptional changes needed for adaptation. The mechanistic target of rapamycin complex 1 (yeast TORC1) kinase integrates nutrient signaling with chromatin regulation, yet whether chromatin signals back to regulate TORC1 remains unclear. We find that a histone H3 lysine 37 to alanine (H3K37A) mutant predicted to disrupt a histone-DNA contact promotes histone degradation, hyperactivates TORC1, and causes cytotoxicity upon TORC1 inhibition. This cytotoxicity is specific to H3K37A, since chromatin instability in wild-type histone H3 (H3WT) cells reduces histone abundance and hyperactivates TORC1 without cytotoxicity upon TORC1 inhibition. Combining H3K37A with these chromatin stability mutants exacerbates histone loss, TORC1 hyperactivity, and lethality during TORC1 inhibition. Transcriptome analysis indicates H3K37A deregulates mitochondrial retrograde signaling, which is exacerbated upon TORC1 inhibition and becomes cytotoxic due in part to defective mitochondrial import of an OXPHOS subunit. Retrograde inactivation, or neutralization of reactive oxygen species (ROS) prevents this toxicity. These findings identify chromatin stability as an upstream restraint on TORC1 signaling, revealing bidirectional communication between chromatin and a major nutrient-responsive pathway. They further show H3K37 has an additional functionality that prevents mitochondrial dysregulation during metabolic stress adaptation.

## Author summary

The cellular nutrient environment regulates gene expression by signaling through the TORC1 kinase to control chromatin structure and function. However, whether chromatin signals back to regulate TORC1 is unknown. We find that a histone H3 lysine 37 to alanine (H3K37A) mutant promotes histone degradation, hyperactivates TORC1, and causes cell death when TORC1 is inhibited. Surprisingly, in cells with wild-type histone H3 (H3WT), other chromatin-destabilizing mutations also reduce histone abundance and hyperactivate TORC1 without causing cytotoxicity upon TORC1 inhibition. The selective cytotoxic effect in H3K37A cells is linked to deregulated mitochondrial retrograde signaling upon TORC1 inhibition that impairs mitochondrial protein import and causes production of toxic mitochondrial reactive oxygen species (ROS). This H3K37A-dependent cell death is completely prevented by genetic inactivation of the mitochondrial retrograde pathway or by ROS neutralization. Collectively, we provide evidence that chromatin stability restrains TORC1 activity, revealing bidirectional communication between chromatin and a major nutrient-responsive pathway. These effects are separable from the selective cytotoxicity caused by H3K37A, indicating that H3K37 has an additional chromatin functionality required for mitochondrial homeostasis during metabolic stress adaptation.

## Introduction

Cells adjust their growth and metabolism to respond to changes in nutrient availability and environmental stress [1]. A central regulator of this adaptive response is the mechanistic target of rapamycin complex 1 (TORC1), a conserved kinase that integrates nutrient inputs with control of transcription, translation, and metabolism to promote cell growth and proliferation [1,2]. Coordinating TORC1 activity with nutrient resource availability prevents deregulated growth or aberrant stress responses that compromise organismal fitness [1,3,4]. In budding yeast, TORC1 is comprised of either the Tor1 or Tor2 kinases along with the Lst8, Kog1, and Tco89 subunits [5,6]. The vacuole (lysosome equivalent) is a degradative compartment that stores amino acids and other metabolites [7]. TORC1 is recruited to the vacuole surface via interactions with the Rag GTPase containing EGO complex which, in conjunction with the V-ATPase, senses vacuole amino acids to activate TORC1 [8–10]. Activated TORC1 then phosphorylates a complex network of substrates that promote anabolism and proliferation, including the Ypk3 kinase that then phosphorylates ribosomal protein S6 (S6ph), while TORC1 simultaneously inhibits the autophagy nutrient stress response [11–15].

Evidence indicates TORC1 regulates epigenetic signaling and transcription in the nucleus through poorly defined mechanisms [16,17]. Epigenetic regulation through histone post-translational modifications (PTMs) controls gene expression, and this process is intertwined with cellular metabolism [18,19]. Many histone PTMs that affect gene expression, including histone methylation and acetylation, depend upon the availability of their metabolic donor molecules, and this interconnectedness orchestrates chromatin accessibility and transcriptional output with the cellular metabolic state [20]. Importantly, recent studies indicate chromatin not only responds to metabolic cues but it may also signal back to regulate nutrient-responsive pathways such as TORC1 [21,22]. These studies suggest reciprocal communication exists between chromatin and TORC1, but the mechanisms underlying this communication remain undefined.

We previously performed a chemical genetic screen utilizing the TORC1-specific inhibitor rapamycin against a library of histone H3 and H4 (H3/H4) mutants to identify relationships between TORC1 and the epigenome [23]. This screen found that TORC1 exhibits genetic interactions with histone H3 N-terminal residues that are PTM sites. Notably, we found that changing H3K37, which is located at the nucleosomal DNA entry/exit point and is predicted to make a critical histone-DNA interaction [24–26], to an alanine (H3K37A) hyperactivates TORC1 and causes cytotoxicity upon TORC1 inhibition [21,23]. H3K37 is adjacent to H3K36 that is mono-, di-, and tri-methylated (H3K36me1/2/3) by the Set2 methyltransferase to maintain chromatin stability during transcription, and Set2 loss also hyperactivates TORC1 [22,27,28]. Set2 maintains chromatin stability by mediating H3K36me2/3 that recruits diverse epigenetic reader proteins to transcribed chromatin, while H3K36me2/3 also actively prevents transcription-linked histone exchange via the histone chaperone Asf1 [29–34]. These previous studies suggest that the H3K36/K37 di-lysine motif contributes to chromatin stability and the restraint of TORC1 signaling.

In this study, we use the H3K37A mutant as a tool to probe how chromatin signals back to regulate TORC1 activity. We demonstrate that H3K37A increases Tom1 ubiquitin ligase-dependent histone degradation via the proteasome which promotes TORC1 hyperactivation. Genetic analyses reveal that Set2-mediated H3K36me2/3, and the Rpd3S HDAC complex that reads H3K36me2/3, act synergistically with H3K37 to maintain histone levels and suppress TORC1 hyperactivation. These effects are not specific to H3K37A since loss of other chromatin stability factors, including the HIR and CAF-1 histone deposition complexes, also reduces histone abundance and hyperactivates TORC1. However, this chromatin instability-induced TORC1 dysregulation is separable from the cytotoxicity caused by TORC1 inhibition, which occurs selectively in H3K37A cells and is enhanced by loss of chromatin stability effectors. Transcriptome analyses indicate that when TORC1 is inhibited, H3K37A deregulates mitochondrial retrograde signaling, and this mitochondrial transcriptional deregulation is compounded by Set2 loss. Furthermore, H3K37A reduces the abundance of the electron transport chain (ETC) Complex IV subunit Cox4, which is associated with impaired mitochondrial membrane potential and protein import, increased ROS, and cell death [35,36]. Inactivating retrograde signaling or neutralizing ROS broadly restores the viability of H3K37A containing chromatin destabilizing mutants when TORC1 is inhibited. Our findings indicate that maintaining chromatin stability is essential for restraining TORC1 signaling, while H3K37 has an additional role in sustaining mitochondrial homeostasis during TORC1 stress adaptation.

## Results

### H3K37 prevents histone destabilization and sensitivity to TORC1 inhibition

We previously found that the H3K37A mutant inhibits HMGB protein chromatin binding and increases both TORC1 signaling and sensitivity to TORC1 inhibition [21,23]. H3K37 is located at the nucleosome DNA entry/exit point [24], and recent structural studies indicate H3K37 contacts nucleosomal DNA [25], potentially to stabilize epigenetic reader binding to methylated H3K36 (Fig 1A) [26]. We confirmed that H3K37A TORC1 inhibition hypersensitivity is due solely to a histone defect by transforming H3WT and H3K37A with either control vector, or vector expressing all four core histone genes, and then spotting them to control media or media containing the TORC1 inhibitor rapamycin. While H3K37A was profoundly growth impaired on rapamycin compared to H3WT, histone expression restored growth (S1A Fig**).** Immunoblot (IB) analysis of both soluble and denatured cell extracts revealed that H3K37A reduces histone levels, which was rescued by histone re-expression **(**Figs 1B–1E and S1B–S1E). To determine if the histone reduction in H3K37A also occurs on chromatin, we analyzed histone H3 density by chromatin immunoprecipitation (ChIP) on two distinct ribosomal protein genes (*RPL3* and *RPL24A*) whose mRNA expression was not affected in the H3K37A mutant (Fig 1F). While H3 density was not altered in the promoters of either gene in H3K37A, H3 levels were significantly reduced in their gene bodies (Fig 1G and 1H), showing H3K37A-associated histone loss extends to chromatin at these loci. These data indicate H3K37A reduces both global and gene-specific histone levels which is linked to increased sensitivity to TORC1 inhibition.

**Fig 1:**
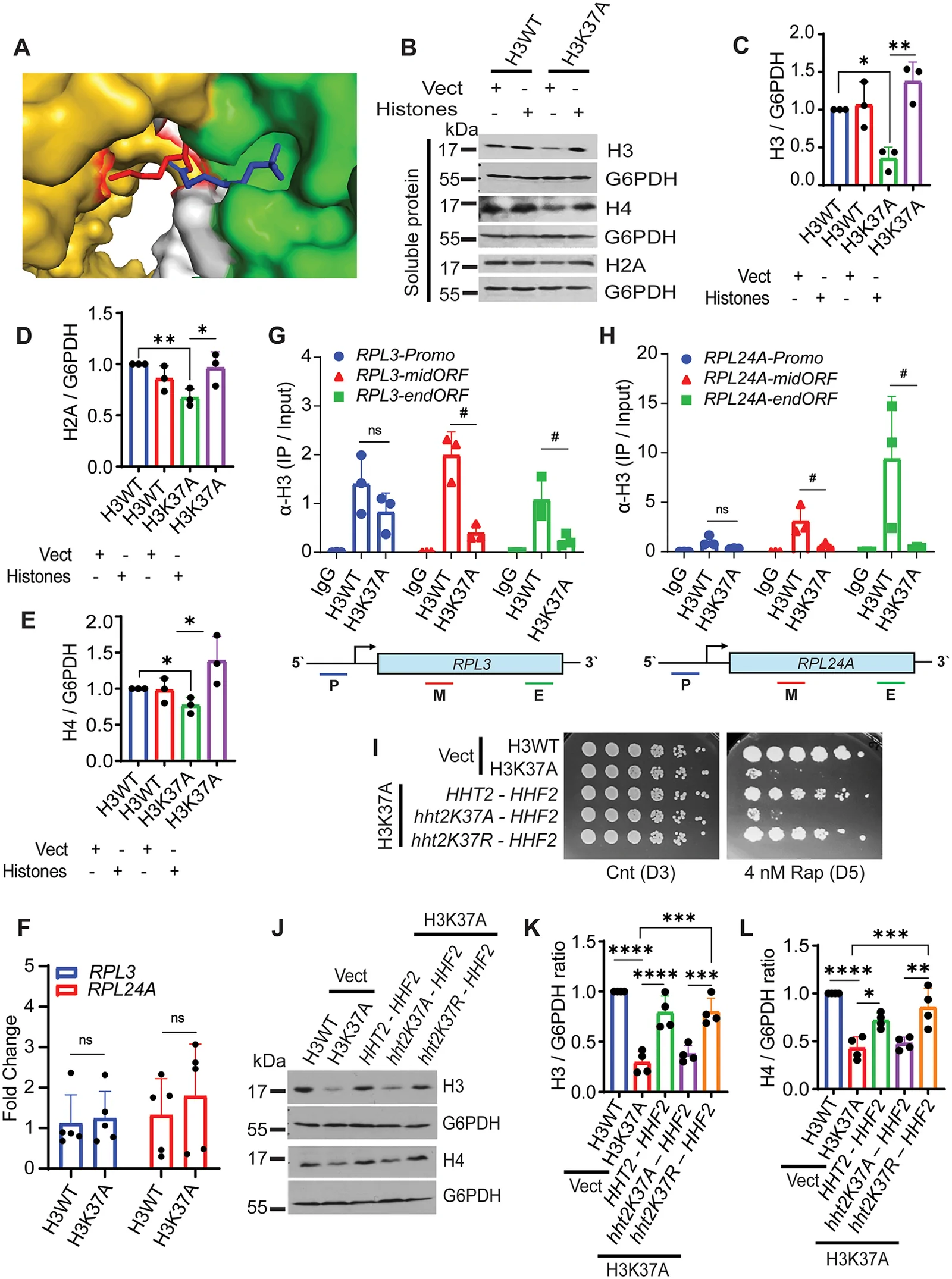
The H3K37A mutant reduces histone expression and causes lethality during TORC1 inhibition. **(A)** The crystal structure of nucleosome-Rpd3S interaction. Red- H3K37; blue- H3K36me3; yellow– DNA; white- histones; green– Eaf3. Structure is PDB ID 8TOF. **(B)** H3WT and H3K37A with control vector or vector expressing all four core histone genes. Soluble protein extracts from H3WT and H3K37A containing the indicated control vector or vector expressing all four histone genes were analyzed by IB. **(C-E)** Quantification of histones H3, H4, and H2A normalized to G6PDH. n = 3; *p < 0.05, **p < 0.01. **(F)** RT-qPCR analysis of *RPL3* and *RPL24A* mRNA levels. n = 5; ns – no significance. **(G and H)** ChIP with IgG or α-histone H3 antibody at the *RPL3* **(G)** and *RPL24A* **(H)** genes. Relative position of the primer sets used for ChIP analysis are indicated below the graphs. n = 3; ns – no significance. ^#^p < 0.05. **(I)** H3K37A transformed with the *HHT2-HHF2, hht2K37A-HHF2*, or *hht2K37R-HHF2* plasmid, along with the control vector in H3WT and H3K37A, were spotted onto the indicated plates for 3-5 days. **(J)** IBs for the total histone H3 and H4 for the cells in **I**. **(K** and **L)** Graphs indicate IB band intensity of H3 and H4 normalized to G6PDH. n = 4; *p < 0.05, **p < 0.01, ***p < 0.001, ****p < 0.0001.

To confirm that H3K37A was solely responsible for these effects, we transformed H3WT and H3K37A with control vector, and H3K37A with vector expressing the wild-type *HHT2-HHF2* histone H3-H4 gene locus, an H3K37A mutation (*hht2K37A-HHF2*), or an H3K37R mutant that restores positive charge (*hht2K37R-HHF2*). Cells were spotted to control or rapamycin media, and H3K37A control cells were severely growth impaired by TORC1 inhibition (Fig 1I). Both *HHT2-HHF2* and *hht2K37R-HHF2* rescued this growth deficiency while *hht2K37A-HHF2* did not (Fig 1I). IB analysis of H3K37A expressing either *HHT2-HHF2* or *hht2K37R-HHF2* revealed that histone expression was restored to H3WT levels, while *hht2K37A-HHF2* had no effect (Fig 1J–1L). Collectively, these data indicate that H3K37A is solely responsible for both the reduction in histone levels and the sensitivity to TORC1 inhibition.

We next determined if H3K37A reduced histone gene expression to explain these effects. H3WT and H3K37A express a single integrated synthetic H3-H4 cassette [37]. Using *HHT1*-specific primers recognizing the synthetic H3 cassette, we performed RT-qPCR to analyze *HHT1, HTA1,* and *HTB1* mRNA expression in H3WT and H3K37A control, and also in H3K37A expressing *HHT2-HHF2* or *hht2K37A-HHF2*. No differences in *HHT1, HTA1, and HTB1* mRNA levels were found between H3WT and H3K37A control, *HHT2-HHF2,* or *hht2K37A-HHF2* expressing cells (S1F–S1H Fig). Additionally, no difference in *HHT2* or *hht2K37A* expression from the exogenous histone vector occurred in H3K37A (S1I Fig). These data indicate that H3K37A reduces histone levels through a post-transcriptional mechanism.

Histones are synthesized during S-phase and are then incorporated into nascent chromatin [38], so we assessed whether cell cycle differences exist between H3WT and H3K37A that explain the histone reduction. Asynchronous H3WT and H3K37A cultures were stained with SYTOX Green and analyzed by flow cytometry, revealing that H3WT and H3K37A have identical cell cycle profiles **(**Figs 2A, S2A and S2B). These data indicate H3K37A reduces histone expression independently of the cell cycle. We next determined whether H3K37A enhanced histone degradation by either autophagy or the proteasome. We blocked autophagy using the autophagy inhibitor phenylmethylsulfonyl fluoride (PMSF) [39,40], but autophagy inhibition did not restore H3K37A histone expression (Fig 2B and 2C). However, proteasome inhibition with MG132 in H3K37A began to reverse the histone loss (for H3) or completely restored histone levels (for H2A) (Fig 2D–2F). We speculated that the H3K37A histone loss may depend on a specific ubiquitin ligase pathway that targets unstable histones. The Tom1 ubiquitin ligase targets excess histones for proteasomal degradation [41], so we generated *tom1*Δ in H3WT and H3K37A and analyzed histone expression. H3WT *tom1*Δ did not affect histones, but H3K37A *tom1*Δ restored H3 and H2A expression back to H3WT levels (Fig 2G–2I). Since reduced histone expression may cause chromatin decompaction [42], we analyzed global chromatin accessibility in H3WT and H3K37A by performing micrococcal nuclease (MNase) digestion. Although H3K37A reduces histone levels (Figs 1B–1E, 1J–1L and S1B–S1E), no global chromatin accessibility difference was detectable between H3WT and H3K37A (S2C Fig). While surprising, a recent report indicates that other histone destabilizing mutants do not alter global chromatin accessibility even though gene-specific chromatin changes do occur [43]. These findings indicate that H3K37A reduces histone levels through a proteasomal dependent degradation pathway involving the Tom1 ubiquitin ligase. Under these assay conditions, the histone reduction was not accompanied by a detectable gross change in global chromatin compaction, although more subtle gene-specific chromatin changes may still exist and not be detected by this approach.

**Fig 2:**
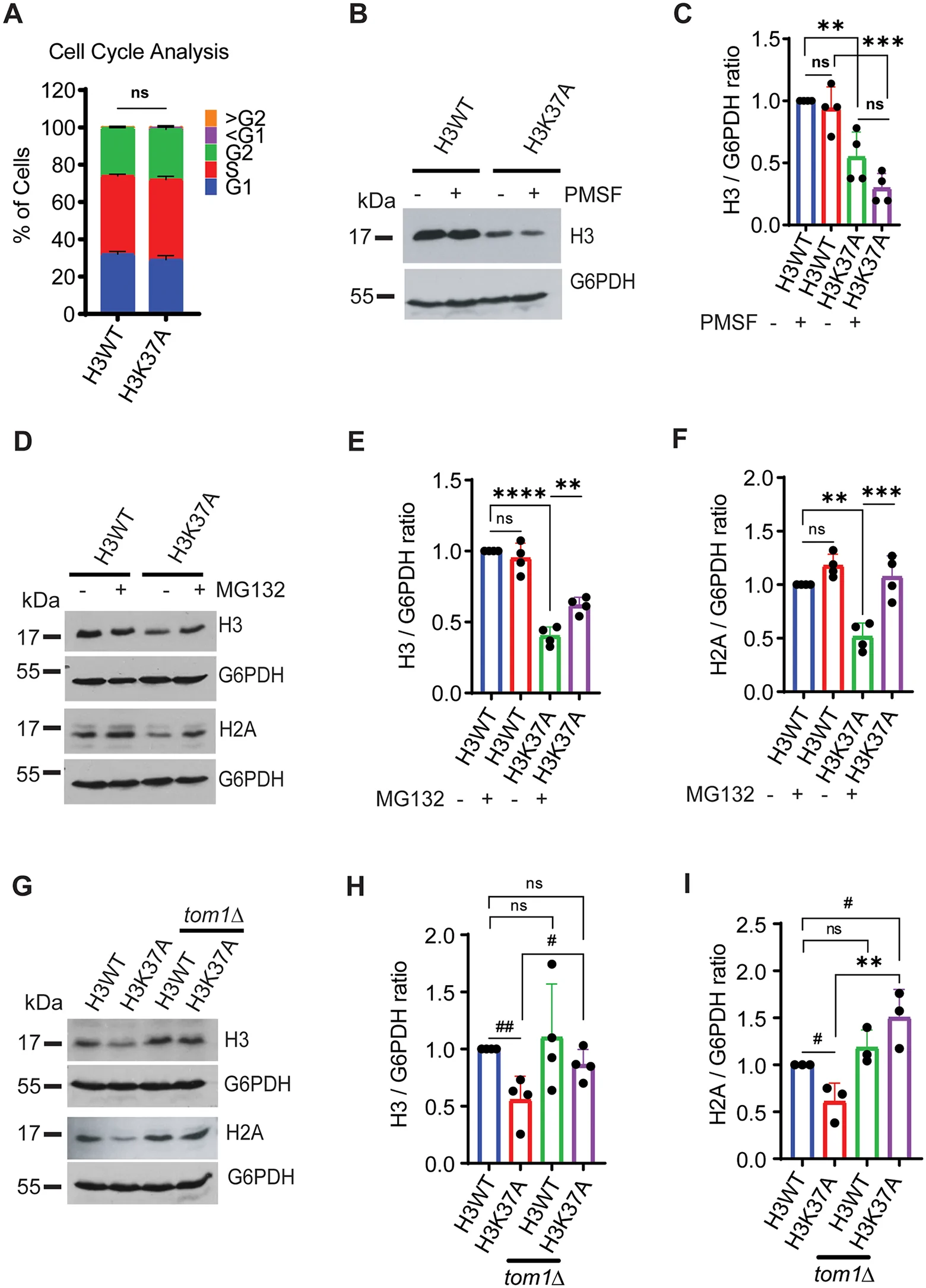
The H3K37A mutant causes histone degradation via the proteasome. **(A)** Cell percentages for each phase of the cell cycle in H3WT and H3K37A. n = 3; ns- no significance. **(B)** H3WT and H3K37A were treated with the autophagy inhibitor PMSF (1 mM) for four hours before histone H3 IB. **(C)** Quantification of histone H3 levels normalized to G6PDH from **B**. n = 4; **p < 0.01, ***p < 0.001. **(D)** H3WT and H3K37A are treated with the proteasome inhibitor MG132 (20 µM) for 30 minutes, and then histones were analyzed by IB. **(E-F)** Quantification of histones H3 **(E)** or H2A **(F)** normalized to G6PDH. n = 3-4; *p < 0.05, ***p < 0.001. **(G)** IB of histones H3 and H2A in H3WT, H3K37A, and their *tom1∆* derivatives **(H-I)** Quantification of histones H3 **(H)** or H2A **(I)** normalized to G6PDH. n = 3-4, ^*#*^*p < 0.05,* ^*##*^*p < 0.01* and **p < 0.01.

### H3K37 and the Set2 methyltransferase collaborate to sustain viability upon TORC1 inhibition

H3K37 is adjacent to H3K36 (Fig 1A) that is methylated co-transcriptionally by Set2, which maintains transcription-linked chromatin stability [28]. We previously found that cells lacking Set2 increase TORC1 signaling [22], so to test if Set2 loss in H3K37A increases TORC1 sensitivity further, *set2*Δ was combined into H3WT and H3K37A. Set2 is one of three histone methyltransferases that regulate transcription, so this analysis also was expanded to examine mutants of these additional methyltransferases. H3WT and H3K37A strains were generated that lack H3K4 methylation due to a Set1 COMPASS complex mutant (*cps50*Δ) or Dot1-dependent H3K79 methylation (*dot1*Δ) [44,45]. All strains were scored for growth on control and rapamycin media and none of the individual methyltransferase mutants in H3WT affected sensitivity to TORC1 inhibition (Fig 3A). As expected, H3K37A was sensitive to TORC1 inhibition and *set2*Δ exacerbated this effect (Fig 3A). We probed H3WT and H3K37A by α-H3K36me3 immunoblot to assess if H3K37A alters Set2-mediated methylation, but we could not detect H3K36me3. This inability to detect H3K36me3 is likely due to disruption of the H3K36me3 epitope recognized by the antibody (Fig 3C). Because the only known Set2 substrate is H3K36 [28], the enhanced sensitivity of H3K37A *set2*Δ to TORC1 inhibition suggests that H3K37A retains some Set2-dependent H3K36 methylation, although a contribution from a non-catalytic role for Set2 cannot be ruled out.

**Fig 3:**
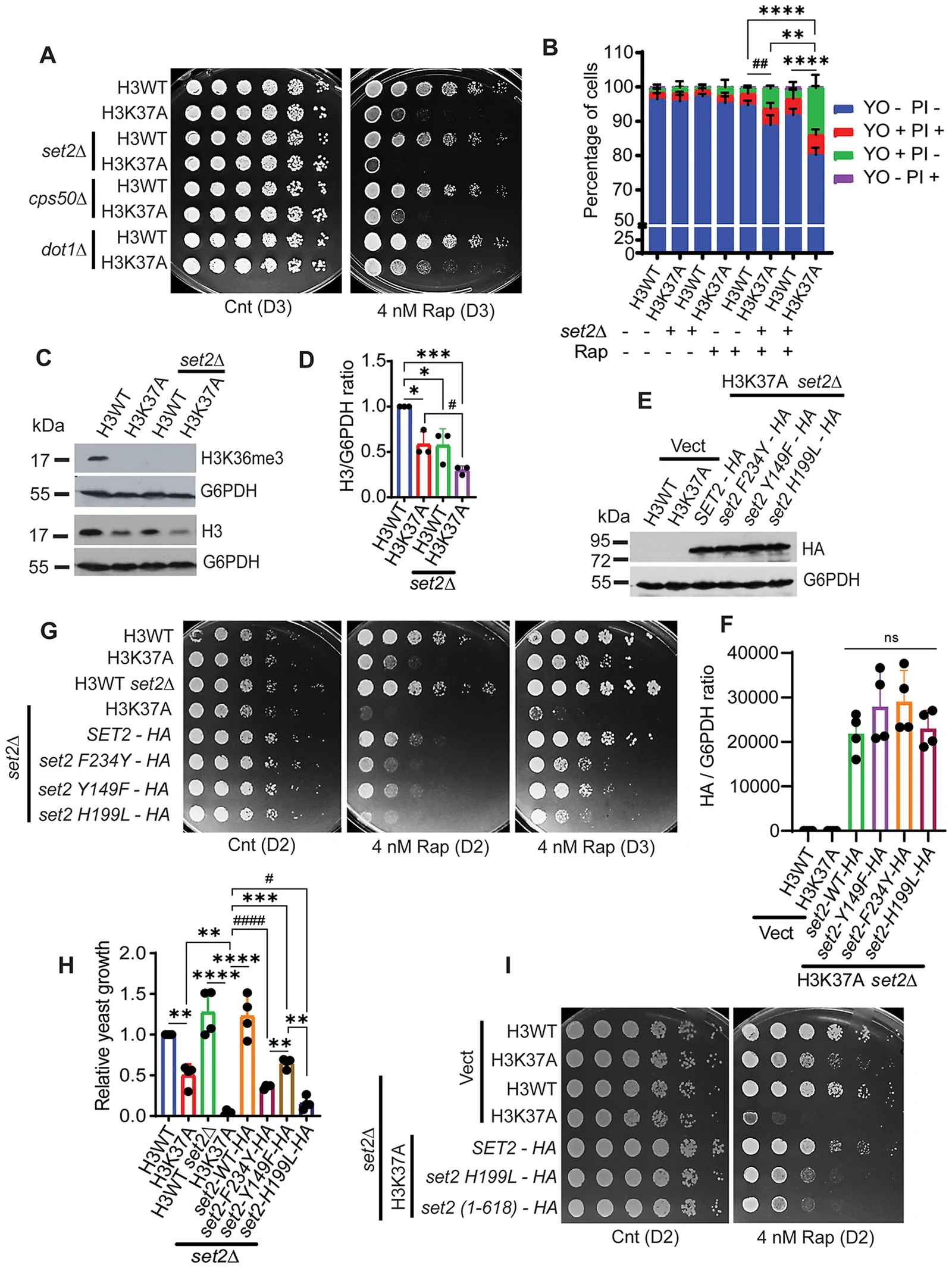
Set2 maintains viability in H3K37A when TORC1 is inhibited through methylation-dependent and -independent mechanisms. **(A)** The *set2*Δ*, cps50*Δ, and *dot1*Δ were spotted onto the indicated plates. **(B)** H3WT, H3K37A, and their *set2*Δ derivatives were mock-treated or 50 nM rapamycin-treated for six hours before YO-PRO-1 and PI staining and flow cytometry analysis. The cell death percentage is represented in the graph. n = 3; **p < 0.01, ****p < 0.0001 and ^*##*^*p < 0.01*. **(C)** IB of H3K36me3 and histone H3 in H3WT, H3K37A, and their *set2*Δ derivatives. **(D)** Quantification of histone H3 normalized to G6PDH from **C**. n = 3; *p < 0.05, ***p < 0.001 and ^*#*^*p < 0.05*. **(E)** Expression of wild-type Set2-HA and the indicated Set2 mutants in H3K37A *set2∆*. **(F)** Quantification of results from **E**. n = 4; ns- no significance. **(G)** Spot assay of the strains transformed with control vector, or vector expressing wild-type *SET2-HA* or the indicated Set2 mutants. **(H)** Quantification of cell density on the rapamycin plate at the third dilution spot (25X). n = 3; **p < 0.01, ***p < 0.001, ****p < 0.0001 and ^*#*^*p < 0.05*, ^*####*^*p < 0.0001*. **(I)** As in **G,** except cells carry control, wild-type *SET2-HA*, the catalytically inactive Set2 (*set2-H199L-HA*), or the SRI domain-truncated Set2 mutant *set2-(1-618)-HA* expression vectors. For **A**, **G**, and **I**, images were captured on the indicated day.

The H3K37A *cps50*Δ caused a negligible increase in sensitivity to TORC1 inhibition, while H3K37A *dot1*Δ modestly rescued this growth defect (Fig 3A). Although COMPASS and Set2 were proposed to methylate H3K37 [46], the rescue of growth by H3K37R during TORC1 inhibition (Fig 1I) or H3K37Q [23], combined with the negligible H3K37A *cps50*Δ effect (Fig 3A), argues against the loss of H3K37 methylation as the primary cause of sensitivity to TORC1 inhibition. As we previously described [21,23], the H3K37R and H3K37Q rescue the TORC1 sensitivity phenotype indicating that H3K37 likely mediates either a protein-DNA or protein-protein interaction that is restored by H3K37R or H3K37Q but not by H3K37A. Set2-mediated H3K36me2/3 prevents nucleosome hyperacetylation to maintain chromatin stability during transcription [28], while Dot1-mediated H3K79 methylation is best understood to promote transcription, but it also has additional roles in DNA damage repair and telomere maintenance [47]. These data indicate that Dot1 promotes sensitivity to TORC1 inhibition in H3K37A, which may be due to its role in transcription, DNA repair, or telomere maintenance, while Set2-mediated H3K36 methylation has a selective role in preventing further sensitization.

We reported the H3K37A sensitivity to TORC1 inhibition is due to cell death [21], so we tested if H3K37A *set2*Δ increased cell death upon rapamycin treatment. H3WT, H3K37A, and their *set2∆* derivatives were mock-treated or treated with 50 nM rapamycin for six hours to ensure robust TORC1 inhibition, stained with YO-PRO-1 (YO, stains apoptotic cells) and propidium iodide (PI, stains late apoptotic and necrotic cells), and analyzed by flow cytometry (Figs 3B and S3A). No cell death was detected in the H3WT, H3K37A, and the *set2*Δ mutants under control conditions (Fig 3B). Although rapamycin had no effect on H3WT or H3WT *set2*Δ, it increased cell death in H3K37A and this was exacerbated by H3K37A *set2*Δ (Fig 3B). To determine if the increased cell death correlated with a further reduction in histone levels, histone H3 was quantified in H3WT, H3K37A, and the *set2*Δ mutants. Both H3K37A and H3WT *set2*Δ reduced H3, while H3K37A *set2*Δ reduced histone H3 levels further (Fig 3C and 3D). Since the H3K37A *dot1*Δ partially rescues growth of TORC1 inhibited cells (Fig 3A), we also quantified histone levels in it, but we did not detect any changes between H3K37A and H3K37A *dot1*Δ (S3B and S3C Fig). Therefore, Set2 loss in H3K37A enhances histone turnover and TORC1 inhibition-induced cytotoxicity, but an increase in histone instability by itself cannot explain the increased cytotoxicity since H3WT *set2*Δ also reduces histones but retains a normal response to TORC1 inhibition.

Set2 mediates H3K36me1/2/3 [28], so we next assessed which H3K36 methylation states contribute to H3K37A *set2*Δ viability when TORC1 is inhibited. H3K37A *set2*Δ was transformed with control vector, or vectors expressing HA-tagged *SET2* wild-type (*SET2-WT*), the *set2-Y149F* mutant that maintains H3K36me3 without H3K36me1/2, the *set2-F234Y* that generates H3K36me1/2 but lacks H3K36me3, or the catalytic mutant *set2-H199L* that ablates H3K36 methylation except for residual H3K36me1 [48]. The Set2-WT and the different mutants were equivalently expressed in H3K37A (Fig 3E and 3F), and they restored the H3K36 methylation states (S3D Fig) [48]**.** Upon TORC1 inhibition, *SET2-WT* rescued H3K37A *set2*Δ viability to that of H3K37A (Fig 3G and 3H). Although less effective than *SET2-WT*, expressing *set2-F234Y*, *set2-Y149F*, or *set2-H199L* in H3K37A *set2*Δ rescued growth relative to H3K37A *set2*Δ control (Fig 3G and 3H)**.** Set2 chromatin recruitment occurs by binding the phosphorylated Pol II C-terminus through the Set2 SRI domain [49,50], and Pol II binding alleviates Set2 autoinhibition to generate H3K36me2/3 [51]. To test if Set2 requires Pol II binding for the response to TORC1 inhibition, we transformed H3K37A *set2*Δ with vectors expressing *SET2-WT-HA*, *set2(1–618)-HA* lacking the SRI domain, or *set2-H199L-HA* catalytic deficient mutant. Spot assays revealed that *SET2-WT* rescued H3K37A *set2∆* back to that of H3K37A control (Fig 3I). Although less efficient than *SET2-WT-HA*, *set2(1–618)-HA* and *set2-H199L-HA* partially restored growth (Fig 3I). The SRI truncated Set2 does not bind Pol II or generate H3K36me3 [50,51], while *set2-H199L* generates minimal residual H3K36me1 [48]. Since no epigenetic reader selectively binds H3K36me1, these data suggest Set2 has methylation-dependent and independent roles in maintaining viability when TORC1 is inhibited that only partially depend on Pol II binding.

To test how loss of the Rph1 H3K36me2/3 demethylase in H3K37A affects the response to TORC1 inhibition [52], *rph1*Δ was introduced into H3WT and H3K37A, and then all strains were scored on control and rapamycin media. While H3K37A *set2*Δ caused synthetic lethality upon TORC1 inhibition, the H3K37A *rph1*Δ modestly rescued growth (Fig 4A), which is consistent with H3K36 methylation in H3K37A suppressing TORC1 inhibition-induced lethality (Fig 3G). H3K36me2/3 binds distinct epigenetic reader proteins, including the Eaf3 subunit of the Rpd3S histone deacetylase (HDAC) that binds H3K36me2/3 [29–31], the Ioc4 subunit of the Isw1b chromatin remodeler that binds H3K36me3 [32], and the Pdp3 subunit of the NuA3b HAT that binds H3K36me3 [33]. To identify readers relevant for the H3K37A response to TORC1 inhibition, we deleted the genes encoding each reader in H3WT and H3K37A and scored them on control or rapamycin media. Loss of either Eaf3 or Rpd3 phenocopied the Set2 mutant (Fig 4A), supporting a role for Rpd3S histone deacetylation in maintaining viability during TORC1 inhibition. Consistent with this concept, we found that H3K23ac levels (an Rpd3S substrate [53]) increased in H3K37A, H3WT *set2∆*, and H3K37A *set2∆* compared to H3WT (S3E and S3F Fig). H3WT *set2*Δ increases histone acetylation due to impaired Rpd3S chromatin binding [28]. These data suggest that H3K37A may modestly either reduce (but not eliminate) H3K36 methylation or disrupt Rpd3S chromatin binding, a possibility that is not unexpected since H3K37 interaction with DNA potentially stabilizes Eaf3-nucleosome interactions (Fig 1A). Intriguingly, the H3K37A *set2*Δ mutant exhibits lower H3K23ac relative to H3WT *set2*Δ (S3E and S3F Fig), although the reason for this reduction remains unclear. The additional H3K36me2/3 reader mutants revealed that H3K37A *ioc4*Δ causes a minor synthetic sick interaction, while H3K37A *pdp3*Δ consistently rescued growth relative to H3K37A (Fig 4B) [33]. Overall, these data indicate H3K37A may increase TORC1 sensitivity to TORC1 inhibition by impinging on chromatin stability.

**Fig 4:**
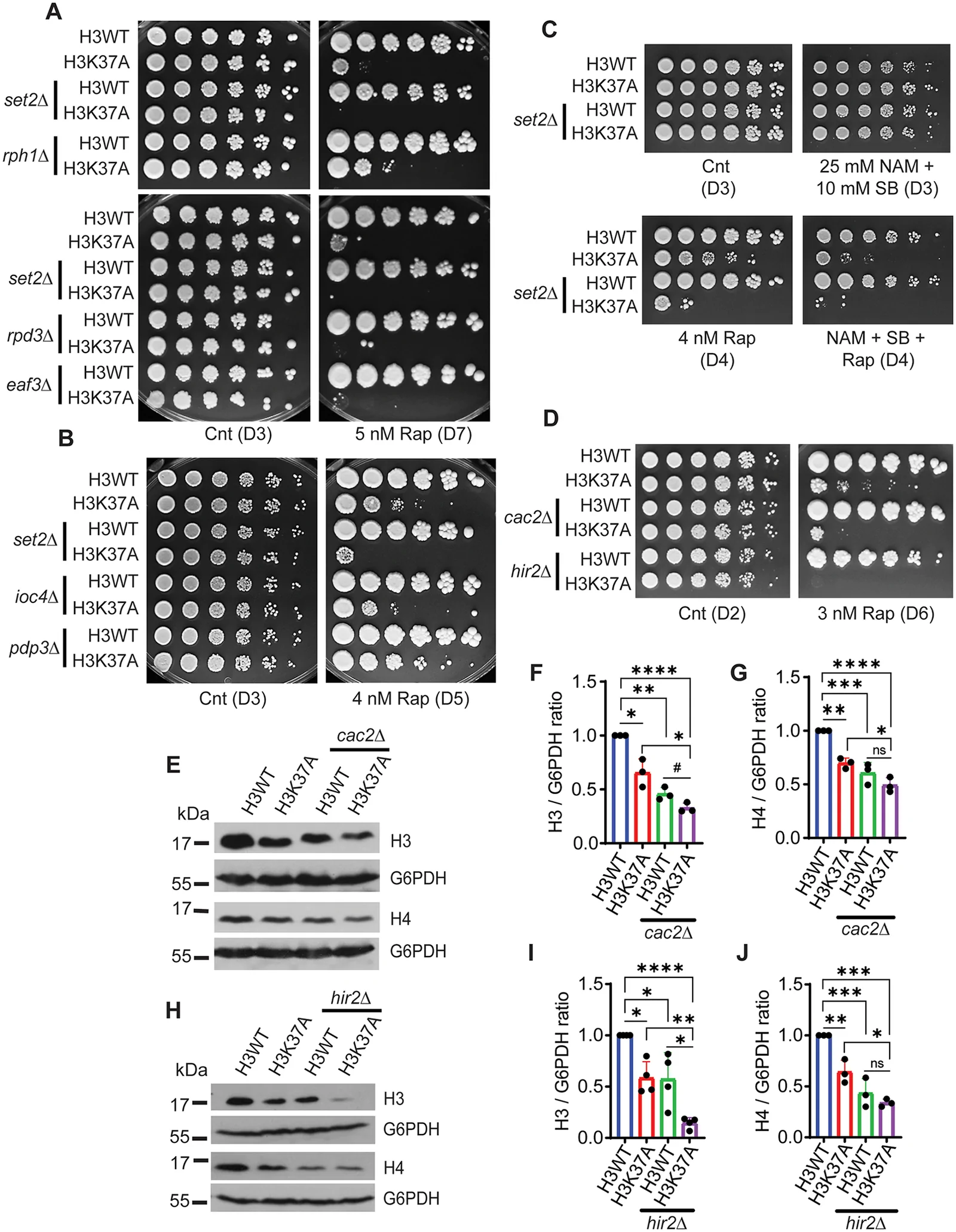
The Set2, HIR, and CAF-1 pathways prevent histone instability and sensitivity to TORC1 inhibition in H3K37A. **(A-B)** The indicated strains were spotted onto control and rapamycin (4-5 nM) plates for 3-7 days. **(C)** H3WT, H3K37A, and their *set2*Δ derivatives cells were spotted onto control plates, or plates containing HDAC inhibitors (25 mM NAM and 10 mM SB), 4 nM rapamycin, or all inhibitors combined for 3-4 days. **(D)** The H3WT and H3K37A *cac2∆* and *hir2∆* were spotted onto the indicated plates for 2-6 days. **(E-G)** IB analysis and histone quantification in H3WT, H3K37A, and their *cac2∆* derivatives. n = 3; *p < 0.05, **p < 0.01, ***p < 0.001, ****p < 0.0001 and ^*#*^*p < 0.05*. **(H-J)** IB analysis and quantification of histones in H3WT, H3K37A, and their *hir2∆* derivatives. n = 4; *p < 0.05, **p < 0.01, ***p < 0.001, ****p < 0.0001.

To independently test this possibility, H3WT, H3K37A, and the *set2*Δ mutants were spotted to control plates, or plates containing rapamycin, the sirtuin (class III HDAC) inhibitor nicotinamide (NAM) [54] and class I/II HDAC inhibitor sodium butyrate (SB) [55], or all three inhibitors combined. H3K37A and H3K37A *set2∆* were not sensitive to combined HDAC inhibition, while both were hypersensitive to TORC1 inhibition (Fig 4C). Combining both TORC1 and HDAC inhibition further increased cell death in H3K37A and H3K37A *set2*Δ, whereas H3WT and H3WT *set2*Δ were unaffected (Fig 4C). Global HDAC inhibition increases histone hyperacetylation to promote nucleosome disassembly and/or histone exchange [56], so these data suggest that enhancing chromatin instability acts synergistically with H3K37A to increase TORC1-inhibition induced cell death.

We probed this possibility further by testing if disrupting other chromatin pathways causes synthetic lethality with H3K37A when TORC1 is inhibited. In H3WT and H3K37A, we deleted the *CAC2* gene that encodes a chromatin assembly factor-1 (CAF-1) subunit that deposits newly synthesized H3-H4 during replication [57], or the *HIR2* gene that encodes a HIR complex subunit that promotes replication-independent histone deposition [58]. Neither H3WT *cac2*Δ nor H3WT *hir2*Δ increased rapamycin sensitivity, but loss of either exacerbated the H3K37A TORC1-inhibition induced cytotoxicity similar to H3K37A *set2*Δ (Fig 4D). Although H3WT *cac2*Δ or *hir2*Δ did not sensitize cells to TORC1 inhibition, histone levels were reduced in both, which was exacerbated by H3K37A (Fig 4E–4J)**.** However, this histone reduction alone does not explain the TORC1 inhibition-induced cell death since H3WT *set2*Δ, H3WT *cac2*Δ, and H3WT *hir2*Δ reduce histone levels without causing TORC1 sensitivity (Fig 4D).

### Histone instability is linked to TORC1 deregulation but is separable from TORC1 inhibition-induced cytotoxicity

We reported that TORC1 signaling increases in both H3K37A and *set2*Δ [21,22], indicating a connection exists between the H3K36/K37 di-lysine motif and TORC1. To probe its mechanistic basis, we assessed TORC1 activity in H3WT, H3K37A, and the *set2*Δ backgrounds by quantifying downstream ribosomal S6 phosphorylation (S6ph) [15]. TORC1 activity increased in H3K37A and H3WT *set2Δ*, while H3K37A *set2*Δ increased it further (Fig 5A and 5B). To confirm this effect is due solely to H3K37A, H3WT and H3K37A were transformed with control vector, while H3K37A was transformed with *HHT2-HHF2*, *hht2K37A-HHF2*, or *hht2K37R-HHF2* expressing vector. TORC1 signaling was higher in H3K37A control compared to H3WT control, while *HHT2-HHF2* and *hht2K37R-HHF2*, but not *hht2K37A-HHF2*, reduced TORC1 activity (Fig 5C and 5D). We also found that compared to H3WT, TORC1 activity was higher in H3WT *cac2Δ* and H3WT *hir2Δ*, but they did not increase it further in the H3K37A background (Fig 5E–5H)**.** Since histone expression in H3K37A was rescued by proteasome inhibition (Fig 2D–2I), we analyzed TORC1 signaling in MG132-treated H3WT and H3K37A. Proteasome inhibition had no effect on TORC1 in H3WT, but it reduced signaling in H3K37A (Fig 5I and 5J)**.** These data suggest that disrupting histone stability (H3K37A), transcription-linked chromatin stability (*set2*Δ), or histone deposition (*cac2*Δ or *hir2*Δ), reduces histone levels and hyperactivates TORC1.

**Fig 5:**
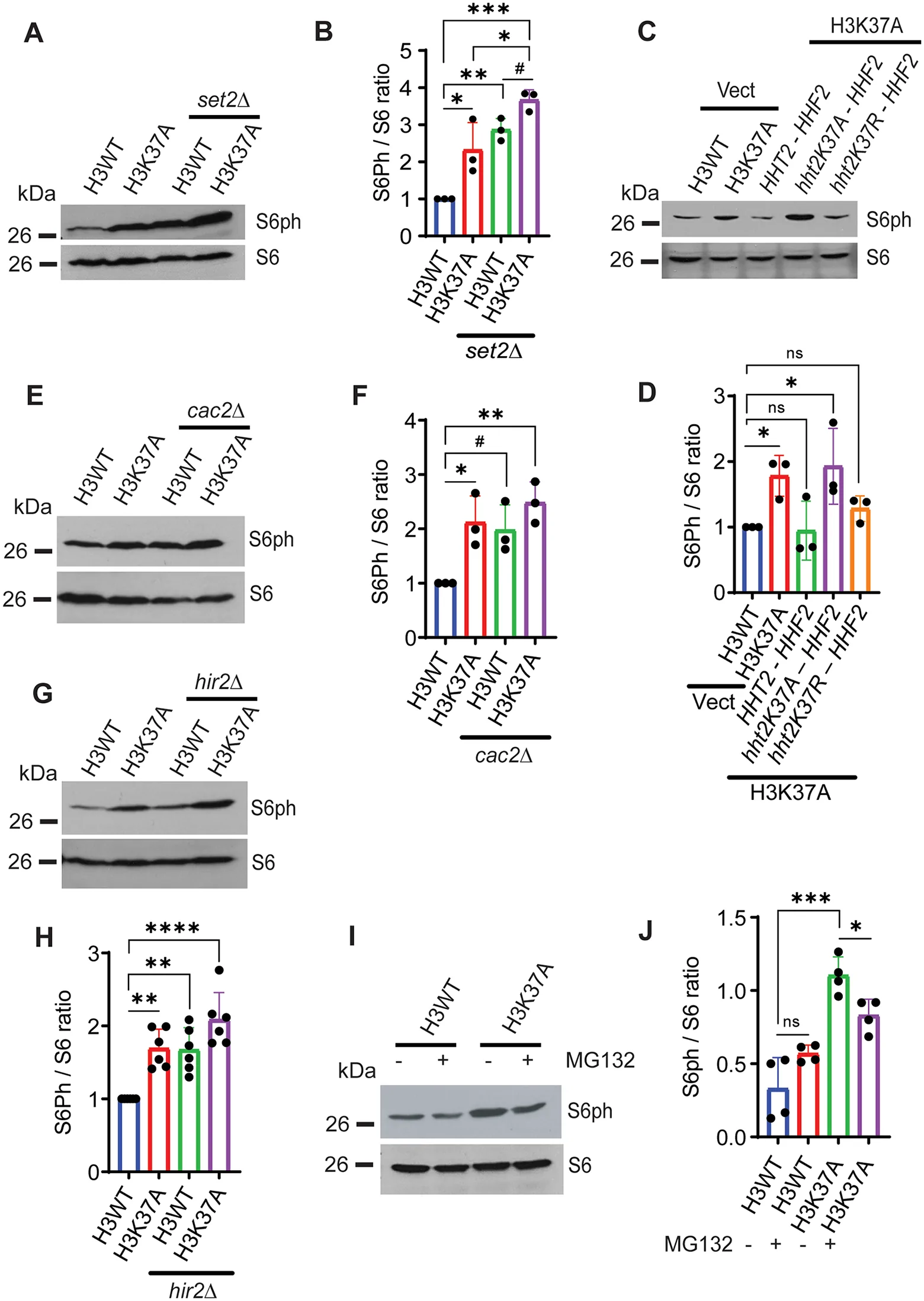
Decreased histone stability correlates with TORC1 hyperactivation. **(A-B)** TORC1 activity in H3WT, H3K37A, and their *set2∆* derivatives. n = 3; *p < 0.05, **p < 0.01, ***p < 0.001 and ^*#*^*p < 0.05*. **(C-D)** As in **A** but TORC1 activity was quantified in H3WT and H3K37A transformed with control vector or vector expressing either H3WT, H3K37A, or H3K37R. n = 3; *p < 0.05. **(E-F)** As in **A** but TORC1 activity in H3WT, H3K37A, and their *cac2∆* derivatives was analyzed. n = 3; *p < 0.05, **p < 0.01, and ^*#*^*p < 0.05*. **(G-H)** As in **A** but TORC1 activity in H3WT, H3K37A, and the *hir2∆* derivatives was analyzed. n = 6; **p < 0.01, ****p < 0.0001. **(I-J)** As in **A** but TORC1 activity was analyzed in H3WT and H3K37A treated with the proteasome inhibitor MG132 (20 µM) for 30 minutes. n = 4; *p < 0.05, ***p < 0.001.

Tco89 is a nonessential TORC1 subunit that recruits TORC1 to the vacuole by interacting with the EGO complex [8,9]. To test if increased TORC1 signaling is sufficient to cause cytotoxicity when TORC1 is inhibited, we transformed H3WT with control or Tco89 overexpressing (*TCO89OE*) vector. Compared to control, *TCO89OE* increased TORC1 activity (Fig 6A and 6B), likely by enhancing TORC1-EGO interactions [8], but it failed to sensitize cells to TORC1 inhibition (Fig 6C)**.** Next, we determined if H3K37A expression in H3WT hyperactivates TORC1 by transforming H3WT with vector expressing *HHT2-HHF2, hht2K37A-HHF2*, or *hht2K37R-HHF2*. Compared to *HHT2-HHF2* cells, *hht2K37A-HHF2* enhanced TORC1 activity whereas *hht2K37R-HHF2* did not (Fig 6D and 6E)**.** To assess if H3K37A expression in otherwise H3WT expressing cells sensitizes them to TORC1 inhibition, we scored them for growth on control media or media containing a lethal (for H3K37A) rapamycin concentration. Higher rapamycin concentrations were used in this experiment since H3K37A competes with H3WT in these cells. *HHT2-HHF2* or *hht2K37R-HHF2* had no effect on rapamycin sensitivity, while *hht2K37A-HHF2* modestly impaired growth (Fig 6F)**.** Since *hht2K37A-HHF2* expression competes with endogenous H3WT in these experiments, the effect H3K7A has is diluted. However, the results do suggest H3K37A is sufficient to increase both TORC1 signaling and, at least in the presence of a higher rapamycin concentration, sensitivity to TORC1 inhibition in H3WT cells. H3WT *set2*Δ, H3WT *cac2*Δ, and H3WT *hir2*Δ reduce histone expression and increase TORC1 activity, but they do not sensitize cells to TORC1 inhibition (Figs 3A–3D, 4D–4J, 5A, 5B and 5E–5H). Therefore, H3K37A causes additional effects beyond reduced histone abundance and TORC1 hyperactivation that are selectively responsible for inducing cytotoxicity when TORC1 is inhibited, possibly by disrupting a key contact residue for HMGB proteins as we previously demonstrated [21,23].

**Fig 6:**
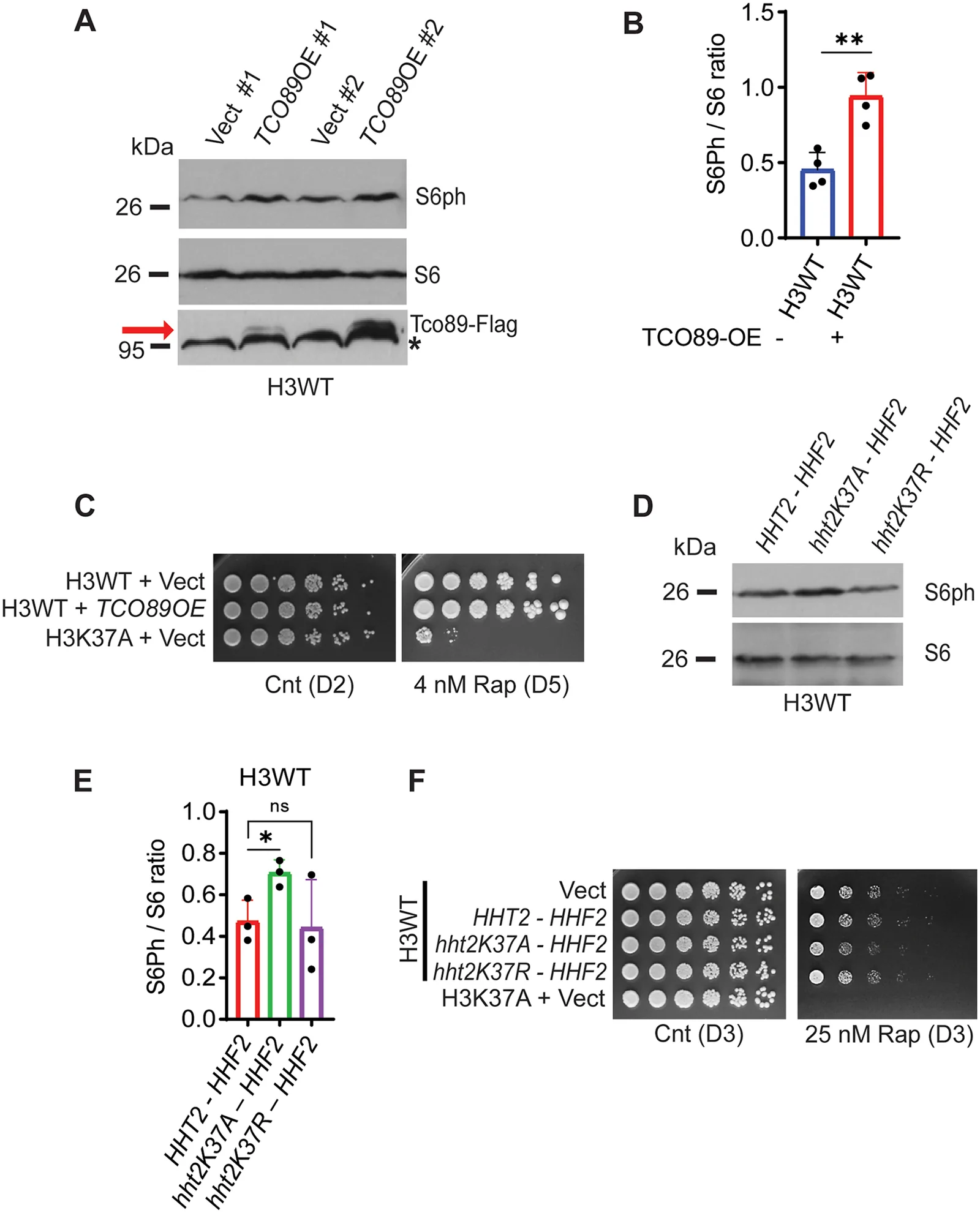
Increased TORC1 activity alone does not cause cell death upon TORC1 inhibition. **(A-B)** H3WT carrying either control vector or vector expressing Tco89-FLAG (*TCO89OE*), was analyzed for TORC1 activity. Two independent transformants are shown. n = 4; **p < 0.01. The red arrow indicates Tco89-FLAG, and the asterisk indicates a cross-reactive protein. **(C)** Cells from **A** were spotted onto the indicated plates for 2-5 days. **(D-E)** TORC1 activity was measured in H3WT expressing either *HHT2-HHF2, hht2K37A-HHF2*, or *hht2K37R-HHF2*. n = 3; *p < 0.05. **(F)** Cells from **D**, along with the control vector H3WT and H3K37A, were spotted onto the indicated plates for three days.

### H3K37A causes mitochondrial transcriptome deregulation that becomes cytotoxic upon TORC1 inhibition

To understand how TORC1 inhibition in H3K37A causes cell death, total RNA was isolated from H3WT and H3K37A that were mock or 20 nM rapamycin treated for one hour, and RNA sequencing (RNA-seq) was performed. Differentially expressed genes (DEGs) and non-coding RNAs (tRNAs, snRNAs, and/or snoRNAs, collectively referred to as DEGs), were defined by comparing each experimental condition to mock-treated H3WT using a 1.5-fold expression change and an FDR < 0.05 as our significance criteria. (S1 Table)**.** Principal component analysis (PCA) showed strong reproducibility among independent replicates (S4A–S4E Fig), with the individual heatmaps shown in S4F and S5 Figs. The mock-treated H3K37A affected 382 DEGs, the rapamycin-treated H3WT altered 140 DEGs, and the rapamycin-treated H3K37A resulted in 1070 DEGs (S1 Table)**.** DEGs were analyzed via the STRING database to identify overrepresented cellular component gene ontologies (GOs) [59]. The mock-treated H3K37A altered GOs annotated to the cell wall, cell periphery, and membrane categories (S6A Fig). Rapamycin-treated H3WT downregulated ribosomal genes (S6B Fig), consistent with TORC1 inhibition repressing ribosomal gene expression [60]. Although rapamycin-treated H3K37A altered the nucleus, nucleolus, and both intracellular and cellular anatomical entity categories, ribosomal gene GOs were not overrepresented (S6C and S6D Fig). The reason for this difference is not clear, but it likely relates to higher TORC1 activity in H3K37A delaying ribosomal gene repression compared to H3WT. The individual gene lists are provided in S2 Table**.** We analyzed the mock-treated H3K37A DEGs but did not find a known TORC1 regulator whose expression was altered, so H3K37A does not deregulate TORC1 through gene expression changes. Our DEGs were then compared to two published *set2*Δ transcriptomes to assess the overlap between H3K37A and Set2 loss [22,61]. Venn analysis of these DEGs identified only 47 genes overlapping between the three studies (S6E Fig and S1 Table), which further supports our genetic analyses that H3K37A does not prevent Set2-mediated H3K36me (Figs 3 and 4).

Manual inspection of the RNA-seq dataset revealed a minor reduction (~1.5-1.7 fold) in multiple histone genes in all three experimental conditions, so we performed RT-qPCR to independently assess their expression. We did not find significant expression differences for these histone genes except for the linker histone *HHO1* that was reduced in H3K37A (S7A–S7F Fig). We tested if reduced *HHO1* mRNA explained the TORC1 sensitivity to TORC1 inhibition by generating a *hho1*Δ in H3WT and H3K37A, but it had no effect on the response to TORC1 inhibition (S7G Fig). A direct comparison of rapamycin-treated H3WT and H3K37A also identified several differentially expressed mitochondrial genes, although mitochondrial categories were not enriched by GO analysis (S4E, S4F and S6D Figs and S1 Table). We next performed GO analysis of the DEGs between mock-treated H3K37A and rapamycin-treated H3K37A to identify GOs relevant to TORC1-inhibition induced cytotoxicity. The rapamycin-treated H3K37A GOs were dominated by mitochondrial GOs, including the respiratory chain complex, oxidoreductase complex, respirasome, mitochondrion, and cytochrome complex (Fig 7A). Manual inspection of these genes revealed most were upregulated in rapamycin-treated H3K37A, which we independently validated by RT-qPCR of some of the most overexpressed mitochondrial genes (*CRC1*, *COX26*, *FMP16*, and *FMP45*). While the rapamycin-treated H3WT did not alter expression of *CRC1* or *COX26*, it caused a minor increase in *FMP16* and *FMP45* expression (S8A–S8D Fig). All four genes were significantly upregulated in rapamycin-treated H3K37A relative to the other conditions (S8A–S8D Fig)**,** and they were increased further by H3K37A *set2*Δ (S8E and S8F Fig). These data indicate that when TORC1 is inhibited, H3K37A deregulates mitochondrial gene expression.

**Fig 7:**
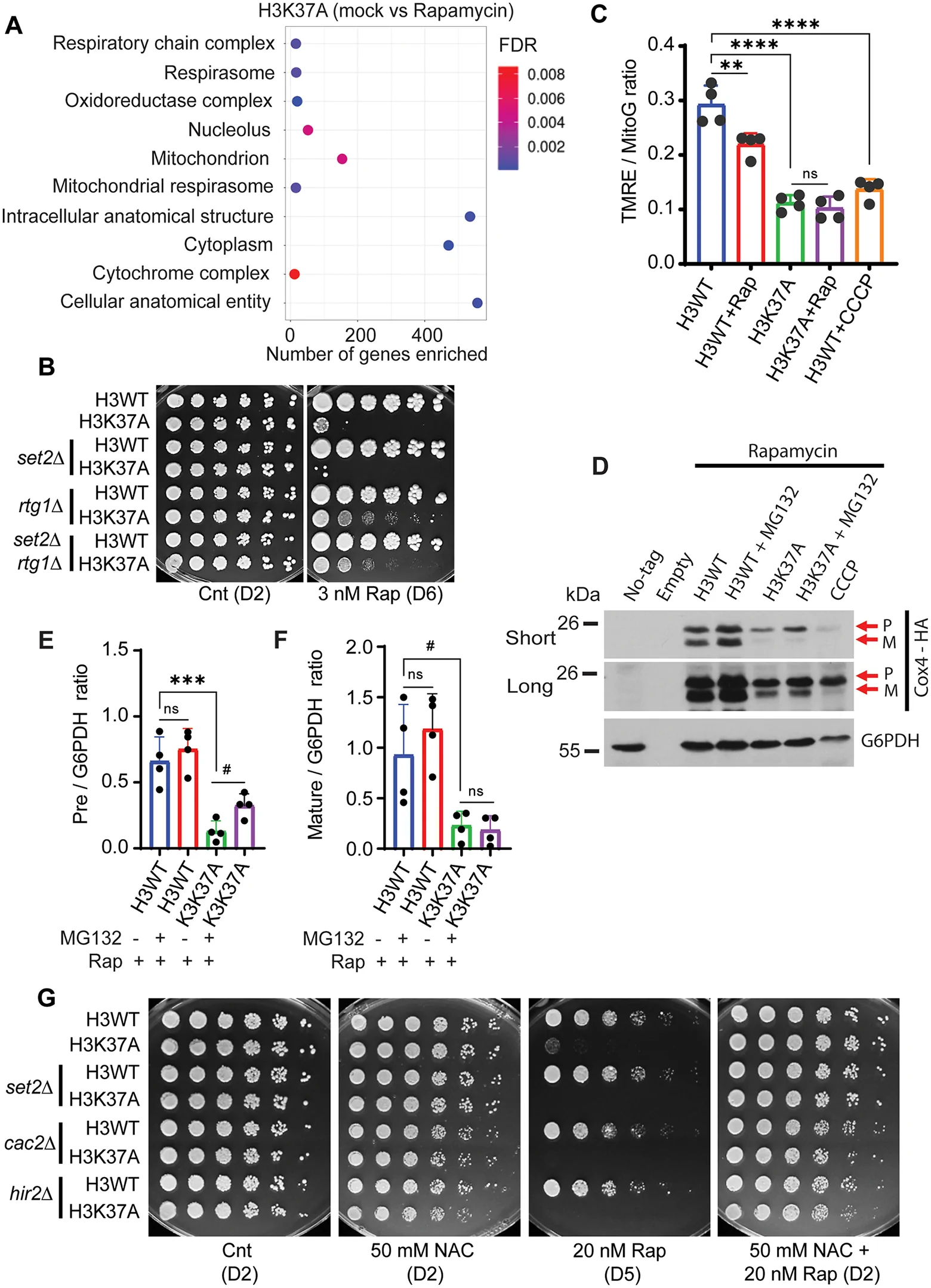
H3K37A deregulates the mitochondrial retrograde response to cause cell death upon TORC1 inhibition. **(A)** Bubble blot analysis of the cellular component GO categories for H3K37A (mock vs rapamycin). FDR – False discovery rate. **(B)** H3WT and H3K37A combined with either *set2*Δ, *rtg1*Δ, or both mutations were spotted onto the indicated plates for 2-6 days. **(C)** H3WT and H3K37A were mock-treated or 20 nM rapamycin-treated for one hour, TMRE and MitoTracker Green stained, and analyzed by flow cytometry. CCCP is an uncoupler used as a positive control. n = 4; **p < 0.01, and ****p < 0.0001. **(D)** IB analysis of Cox4-HA in mock or MG132-treated (20 µM) for 30 mins, and in rapamycin-treated H3WT and H3K37A for 1 hour. P- pre-protein and M-mature protein. **(E)** Quantification of precursor Cox4-to-G6PDH ratio. n = 4; ^***^p < 0.001, and ^*#*^*p < 0.05*. **(F)** Quantification of mature Cox4-to-G6PDH ratio. n = 4; ^*#*^*p < 0.05*. **(G)** H3WT and H3K37A and their *set2*Δ, *cac2*Δ, or *hir2*Δ were spotted to control media, or media containing 50 mM NAC, 20 nM rapamycin, or both inhibitors for 2-5 days.

### H3K37A selectively deregulates mitochondrial retrograde signaling, impairs mitochondrial protein import, and causes TORC1 inhibition-induced cell death

TORC1 inhibition induces transcription of nuclear-encoded mitochondrial genes (mitochondrial retrograde signaling) through the transcription factors Rtg1 and Rtg3, which enhances mitochondrial content and metabolism [62]. We previously found that H3K37A increased ROS compared to H3WT, and that the retrograde gene *CIT2* was induced to higher levels in rapamycin-treated H3K37A relative to rapamycin-treated H3WT [21,62]. Our RNA-seq data indicated that *CIT2* and *CIT1* (another retrograde-activated gene) were overexpressed in rapamycin-treated H3K37A versus rapamycin-treated H3WT (S8G and S8H Fig**)**, suggesting H3K37A may deregulate the retrograde response to cause TORC1 inhibition-induced cell death. We tested this possibility by deleting *RTG1* in H3WT, H3K37A, and their *set2∆* derivatives to block retrograde activation, and then scored them for growth on control or rapamycin-containing media. Although H3K37A and H3K37A *set2*Δ exhibited cytotoxicity (Fig 3B), the viability of both was restored by *rtg1*Δ (Fig 7B). This rescue occurred even though H3K37A *rtg1*Δ and H3K37A *set2*Δ *rtg1*Δ are profoundly slow growing compared to H3WT *rtg1*Δ and H3WT *set2*Δ *rtg1*Δ (Fig 7B). We validated that *rtg1*Δ blocked retrograde activation due to TORC1 inhibition by confirming that *CIT1* and *CIT2* mRNA induction was dramatically impaired (*CIT1*) or completely prevented (*CIT2*) (S8I and S8J Fig). Intriguingly, we also noted that mock-treated H3K37A exhibited increased expression of both *CIT1* and *CIT2* (S8I and S8J Fig), suggesting that it induces low-level retrograde activation even without TORC1 inhibition. To test if the rescue of H3K37A and H3K37A *set2*Δ viability by retrograde inactivation was due to ROS inhibition, we mock-treated or 50 nM rapamycin-treated cells for 24 hours and quantified ROS by dihydroethidium (DHE) staining. ROS were not different under control conditions (S9A Fig), but TORC1 inhibition increased ROS in both H3K37A and H3K37A *set2*Δ, which was blocked by *rtg1*Δ (S9B Fig). These data indicate that retrograde deregulation in H3K37A and H3K37A *set2∆* causes excessive ROS and TORC1 inhibition-induced cytotoxicity.

Loss of mitochondrial membrane potential (MMP) triggers retrograde signaling [63], so we tested if H3K37A affects MMP. H3WT and H3K37A were mock-treated or treated with 20 nM rapamycin for one hour before staining with the mitochondrial dyes tetramethylrhodamine ethyl ester (TMRE), whose incorporation depends on MMP, and with MitoTracker Green (MitoG), whose mitochondrial incorporation is MMP independent [64]. All samples then were analyzed by flow cytometry, and the TMRE signal was normalized to the MitoG signal to control for mitochondrial content differences (Figs 7C and S9C) [64]. Treatment of H3WT with the mitochondrial uncoupler carbonyl cyanide m-chlorophenyl hydrazone (CCCP) confirmed the assay measures MMP since CCCP treatment reduced the TMRE/MitoG ratio (Fig 7C). In H3WT, TORC1 inhibition modestly reduced MMP compared to the H3WT control (Fig 7C), indicating TORC1 inhibition decreases MMP, while H3K37A reduced MMP compared to H3WT without TORC1 inhibition (Fig 7C). This H3K37A MMP reduction is consistent with the low level of retrograde signaling activation that is indicated by increased *CIT1* and *CIT2* expression (S8I and S8J Fig). Importantly, the MMP in H3K37A was not reduced further by TORC1 inhibition, suggesting that TORC1 inhibition and H3K37A may act through the same pathway to inhibit MMP. To determine whether this H3K37A retrograde activation and reduced MMP were linked to histone instability, or if they were due to a histone-instability independent role of H3K37A, we analyzed retrograde activation and MMP in H3WT and the H3WT *cac2*Δ, *hir2*Δ, and *set2*Δ derivatives. Surprisingly, we find that even though these mutants increased histone instability (Figs 3 and 4), the *cac2*Δ, *hir2*Δ, and *set2*Δ mutants did not increase retrograde activation unless TORC1 was inhibited as indicated by *CIT2* expression (S10A Fig). Furthermore, neither *cac2*Δ nor *set2*Δ altered MMP, while *hir2*Δ increased MMP (S10B and S10C Fig). Because the H3K37A *dot1*Δ partially rescued growth upon TORC1 inhibition (Fig 3A), but it does not rescue histone levels (S3B and S3C Fig), we also analyzed whether it affects MMP. Intriguingly, we found that H3K37A *dot1*Δ increased MMP relative to H3K37A under both mock-treated and TORC1-inhibited conditions (S11A and S11B Fig). These results suggest that enhancing MMP in H3K37A *dot1*Δ partially mitigates the cytotoxic effects that occur upon TORC1 inhibition. Collectively, these data strongly suggest that H3K37 provides a unique functionality to chromatin beyond maintaining histone stability and inhibition of TORC1 signaling that is required to prevent MMP loss and TORC1 inhibition-induced cell death.

Mitochondrial MMP controls mitochondrial protein import [65], so we tested if the reduced MMP in H3K37A causes mitochondrial protein import defects [65]. Analysis of the RNA-seq data indicated that *COX4* mRNA, which is a nuclear-encoded ETC Complex IV subunit, is not affected in any of the experimental samples, which we validated by RT-qPCR (S12A Fig)**.** Cox4 is synthesized in the cytoplasm as a precursor protein that is then imported into the mitochondria where the N-terminal targeting sequence is cleaved to generate mature Cox4 protein [35,65]. Cox4 is essential for Complex IV assembly [66], and its loss reduces MMP and activates the retrograde response [63], so we analyzed Cox4 mitochondrial import to assess if the H3K37A MMP inhibition causes mitochondrial protein import defects. To do so, we genomically tagged Cox4 with a 3XHA epitope at its C-terminus in H3WT and H3K37A to use as a reporter for monitoring mitochondrial protein import. In glucose conditions, mitochondrial proteins are expressed at low levels, so to test if H3K37A affects Cox4 import, we treated Cox4–3XHA H3WT and H3K37A with rapamycin to activate retrograde signaling [67,68]. As a control, H3WT was treated in parallel with CCCP to dissipate the MMP. In rapamycin-treated H3WT, Cox4 migrated as a doublet with the uppermost band corresponding to the preprotein since it accumulates in H3WT CCCP-treated cells (Fig 7D). Importantly, rapamycin-treated H3K37A reduced both the preprotein and mature Cox4 forms **(**Fig 7D–7F**)**. When mitochondrial preproteins accumulate in the cytoplasm due to mitochondrial import defects, cells use multiple quality control pathways to enhance their proteasome degradation [65]. To test if Cox4 reduction in H3K37A is due to enhanced proteasome-mediated degradation, H3K37A was treated with MG132 and Cox4 was then analyzed. Proteasome inhibition increased Cox4 preprotein levels, but it did not affect mature Cox4 (Fig 7D–7F). Interestingly, proteasome inhibition did not increase Cox4 preprotein levels when cells were grown without TORC1 inhibition (S12B–S12D Fig). These data suggest that mitochondrial protein import is significantly impaired only when TORC1 is inhibited. They also suggest H3K37A inhibits Cox4 expression through a translational control mechanism since *COX4* mRNA expression is unaffected (S12A Fig), and proteasome inhibition does not restore Cox4 levels (Figs 7D and S12B). These results suggest retrograde transcriptional deregulation in H3K37A may overwhelm the mitochondrial protein import machinery to cause TORC1-inhibition induced cytotoxicity.

Cox4 is an essential ETC Complex IV subunit and disrupting ETC stoichiometry increases ROS and causes cell death [35,36]. We tested if ROS deregulation explains the H3K37A TORC1 inhibition-induced cytotoxic response by scoring H3WT, H3K37A, and their *set2*Δ, *hir2*Δ, and *cac2*Δ derivatives on control media, media containing either 20 nM rapamycin (which is lethal for H3K37A) [21,23], 50 mM of the antioxidant N-acetyl cysteine (NAC), or both conditions. H3WT backgrounds grew upon TORC1 inhibition, NAC exposure, or with both conditions combined (Fig 7G). Upon TORC1 inhibition alone, all H3K37A backgrounds failed to grow, consistent with this level of TORC1 inhibition being lethal in H3K37A; however, combined TORC1 and ROS inhibition completely restored their viability (Fig 7G). To confirm NAC did not prevent TORC1 inhibition by rapamycin, we assessed TORC1 signaling and found that NAC treatment does not prevent rapamycin-mediated TORC1 inhibition (S12E Fig). Therefore, these findings support a model in which the TORC1 inhibition-induced deregulated retrograde response in H3K37A, combined with defective mitochondrial protein import and altered stoichiometry of Cox4 and possibly other ETC subunits, contributes to ROS accumulation and cell death. Collectively, these data provide strong evidence that chromatin stability acts broadly to restrain TORC1 signaling, whereas H3K37A provides an additional function needed to prevent mitochondrial dysregulation and cell death during TORC1 stress, which is likely due at least in part to its role as a histone contact residue for binding of HMGB proteins [21,23].

## Discussion

Herein, we demonstrate that disrupting pathways that stabilize chromatin, either by blocking a critical histone H3 N-terminal tail interaction with DNA (the H3K37A mutant), interfering with transcription-coupled chromatin stability (Set2-Rpd3S pathway mutants), or impairing nucleosome deposition by compromising the HIR or CAF-1 pathways, reduces histone abundance and hyperactivates TORC1 signaling. Utilizing the H3K37A mutant to probe the underlying mechanisms involved, we demonstrate that H3K37 maintains histone stability by preventing Tom1 ubiquitin ligase-dependent histone degradation via the proteasome. Thus, disrupting a stabilizing histone-DNA interaction promotes histone degradation via a pathway originally identified to mediate degradation of excess histones [41].

H3K37 interacts with DNA to stabilize the nucleosome and/or the binding of additional chromatin proteins, including HMGB proteins as we previously demonstrated [21,23]. A role for H3K37 in stabilizing the nucleosome is supported by two independent structural analyses of either Set2 or Rpd3S bound to the nucleosome that reveal H3K37 is oriented away from H3K36 towards the DNA [25,26]. The loss of the Set2, HIR, or CAF-1 chromatin stability pathways in the H3K37A mutant further decreases histone stability, while their disruption also hyperactivates TORC1 signaling in H3WT cells. These results support the concept that reduced histone abundance and increased chromatin instability signals back to hyperactivate TORC1 independently of altered gene expression. Intriguingly, we find that H3K37A increases histone degradation without altering global chromatin accessibility. We would emphasize that while we find no gross chromatin compaction changes occur in H3K37A, there are likely gene-specific chromatin compaction changes occurring in H3K37A that will only be detectable by more refined analyses utilizing approaches such as ATAC-seq. However, our results mirror those findings from a recent report demonstrating that histone destabilizing mutants do not increase global chromatin accessibility, but they do change gene-specific transcription [43].

How chromatin disruption, either through disruption of H3K37, or the Set2, CAF-1, or HIR pathways, causes TORC1 hyperactivation currently remains unclear. Our results for the H3K37A mutant indicate that gene expression changes do not account for the increased TORC1 activity. One potential explanation could be that chromatin destabilization results in increased histone degradation by the proteasome, which boosts free amino acid levels that then are funneled into vacuole-dependent or independent pathways to activate TORC1 [2]. Because of the large histone complement needed for genome packaging, it is not unreasonable to speculate that even a small percentage increase in histone degradation boosts intracellular amino acids to enhance TORC1 activity. Although TORC1 is an epigenetic regulator [16,17], such a mechanism would indicate that changes to the chromatin environment also signals back to regulate TORC1 activity.

Because H3K37A disrupts the epitope used to generate H3K36me antibodies, we cannot reliably assess if it affects H3K36me. The enhanced sensitivity of H3K37A *set2∆* suggests that H3K37A retains some Set2-dependent H3K36 methylation, although a contribution from a non-catalytic role for Set2 cannot be ruled out. These results also remain compatible with a partial reduction in H3K36 methylation or altered Rpd3S chromatin binding in H3K37A. Structural studies of either Set2 or Rpd3S bound to the nucleosome indicate that H3K37 is oriented away from H3K36 where it contacts nucleosomal DNA [25,26]. While H3K37A is expected to reduce H3-DNA interactions, it likely does not completely compromise either Set2-mediated H3K36 methylation or Rpd3S chromatin binding and histone deacetylation, possibilities that are consistent with our genetic analyses. Surprisingly, both the Set2 catalytically deficient mutant, and the SRI-truncated Set2 mutant, partially rescue viability of H3K37A *set2*Δ upon TORC1 inhibition, supporting potential methylation-dependent and non-catalytic roles for Set2 when TORC1 is inhibited. A recent study reported that mice lacking the Set2 homolog Setd2 hyperactivate TORC1 signaling similar to what occurs in yeast [22,69,70]. Thus, the Set2/SETD2 pathway may have an evolutionarily conserved role in restraining TORC1 activity. Since human SETD2 functions as a tumor suppressor in multiple tumor types [71], and TORC1 deregulation is a driving force in a majority of cancer, this functional relationship between the Set2/SETD2 and TORC1 pathways has important ramifications for SETD2-mediated tumor suppression [71]. Although Set2 has no known non-histone substrates, SETD2 methylates some non-histone substrates [71]. An intriguing possibility could be that Set2 and SETD2 methylate a conserved non-histone substrate independently of Pol II binding required for mediating the response to TORC1 inhibition. Set2 interacts with an array of non-histone proteins as annotated in the BioGRID database, including ribosomal proteins that would be relevant to TORC1-dependent translational regulation [72]. Recently, a SET domain methyltransferase was shown to methylate ribosomal proteins [73], so it will be interesting to test whether Set2 and SETD2 methylate ribosomal proteins to sustain viability when TORC1 activity is inhibited.

Although reduced histone abundance and TORC1 hyperactivation are shared by H3K37A and other chromatin stability mutants, only H3K37A causes basal mitochondrial membrane depolarization and low-level retrograde activation. Thus, H3K37 provides an additional function required for surviving TORC1 inhibition. TORC1 regulates mitochondrial metabolism by inhibiting retrograde signaling [68], and we demonstrate that when TORC1 is inhibited, H3K37A deregulates retrograde signaling and causes cell death, with this phenotype further exacerbated when H3K37A is paired with Set2, HIR, or CAF-1 pathway mutants. How this transcriptional deregulation occurs is unclear, but one possibility could be that chromatin destabilization through these mechanisms reduces promoter nucleosome stability at retrograde-inducible genes. While constitutively expressed genes have a nucleosome-free, or nucleosome-depleted, promoter structure, inducible genes that include many metabolic responsive genes have positioned promoter nucleosomes that must be remodeled for transcriptional induction [74]. Previous studies demonstrated that chromatin destabilizing mutants activate mitochondrial gene expression in repressive nutrient conditions [75]. Since H3K37A, as well as Set2, CAF-1, and HIR mutants, reduce histone levels, their promoter nucleosomes at retrograde-activated genes may either be reduced or more fragile and susceptible to remodeling, leading to their transcriptional deregulation.

Retrograde activation normally does not cause cell death as it is a metabolic response for surviving nutrient stress [62]. Our results indicate that the TORC1 inhibition-induced cytotoxicity in H3K37A, either in isolation or when paired with additional chromatin destabilizing mutants, is separable from H3K37 prevention of histone turnover and TORC1 hyperactivation. These data indicate that H3K37 provides an additional chromatin-mediated function required for cell survival when TORC1 activity is decreased. H3K37 is a contact residue for the mammalian HMGB1 protein to bind nucleosomes *in vitro* [76,77], and our previous work demonstrated that H3K37A disrupts chromatin binding of a subset of yeast HMGB proteins [21,23]. Non-chromatin bound mammalian HMGB1 can localize to mitochondria where it can modify mitochondrial function [78,79]. We suggest the possibility that H3K37A inhibits chromatin binding of a subset of HMGBs that then relocate to the cytoplasm where they contribute to TORC1 hyperactivation, which we previously suggested occurs [21]. These cytoplasmic HMGBs may also cause low level mitochondrial stress as supported by our data that indicate H3K37A reduces MMP even in the absence of TORC1 inhibition. Under normal growth conditions this mitochondrial stress is buffered and remains insufficient to cause cell death. However, upon H3K37A TORC1 inhibition and the corresponding retrograde transcriptional deregulation, the increase in cytoplasmic mitochondrial proteins likely overwhelms the mitochondrial import machinery due to the impaired H3K37A MMP since MMP is required for mitochondrial protein import [65]. The accumulating cytoplasmic mitochondrial proteins likely activate a proteostatic stress response to cause cell death as has been documented occurs in other scenarios of mitochondrial protein import stress [80,81]. Such a mechanism also would explain why pairing H3K37A with other mutants that increase histone turnover becomes synthetic lethal when TORC1 activity is impaired. The histone H3 N-terminal tail provides additional stabilizing contacts for HMGB nucleosome binding [76,77], so enhanced histone turnover and chromatin instability in H3K37A would be expected to exacerbate the HMGB chromatin binding defects, causing more cytoplasmic HMGBs to accumulate and an even greater level of mitochondrial stress upon TORC1 inhibition.

Another possible explanation for the cytotoxic response to TORC1 inhibition in H3K37A is that the retrograde transcriptional deregulation may be uneven, leading to imbalances in mitochondrial gene expression that become cytotoxic when TORC1 activity is impaired. Stoichiometric production of ETC components is critical for mitochondrial function, while ETC subunit imbalances due to defective transcription, translation, and/or mitochondrial protein import cause elevated ROS and cell death [35,36]. Consistent with this possibility, we find that H3K37A inhibits expression of the ETC Complex IV subunit Cox4 through what appears to be a post-transcriptional mechanism. This Cox4 reduction likely contributes to the reduced MMP since ETC activity maintains MMP to promote mitochondrial protein import [35,65]. Indeed, we demonstrate that H3K37A impairs Cox4 mitochondrial import when TORC1 is inhibited, which leads to proteasome-mediated degradation of the Cox4 preprotein. Since Cox4 is critical for Complex IV assembly [66], and its loss increases ROS [63], we speculate that chromatin destabilization by H3K37A also perturbs ETC function in part through impaired Complex IV function. Indeed, the reduced MMP in H3K37A likely causes a generalized defect in mitochondrial protein import of other ETC subunits. Upon TORC1 inhibition, the combination of the deregulated retrograde transcriptional response combined with mitochondrial ETC protein import defects likely increases proteostatic stress and ROS production to cause cell death.

Histone destabilizing mutants alter gene expression or chromosome function to impair development and stress resilience [43]. Mitochondria are also sensitive to chromatin stability as mutants that perturb chromatin deregulate mitochondrial function in yeast and metazoans [75,82,83]. These phenotypic effects mirror changes that occur during aging, and both worm and fly models expressing histone destabilizing mutants exhibit age-related functional decline [43,82,83]. Yeast and human cells expressing histone destabilizing mutants also exhibit defective environmental stress responses [43], supporting a role for chromatin stability in maintaining stress responsiveness [84]. Aging causes epigenome deterioration due to reduced histone expression [85,86], while TORC1 activity increases during aging and TORC1 inhibition extends longevity [87]. We speculate that aging-dependent histone loss may fuel the increased TORC1 signaling if the histones are degraded and their amino acids recycled back into TORC1 activation pathways. Future experiments will delineate the extent of this TORC1-epigenetic bidirectional communication, and whether its deregulation contributes to aging or diseases caused by TORC1 dysregulation, including cancer.

## Materials and methods

### Yeast strains, plasmids, and cell growth conditions

The YNL406 (H3WT) and YNL403 (H3K37A) parent strains used are *ura3∆* derivatives from the histone H3/H4 library [37]. Strain engineering was performed as previously described [88]. All strains used in this study are listed in S3 Table**.** Unless otherwise specified, strains were cultivated in either nutrient-rich YPD (1% yeast extract, 2% peptone, and 2% glucose) or in nutrient-defined SC (0.2% yeast nitrogen base devoid of amino acids, 0.5% ammonium sulfate, 2% dextrose, and 0.19% amino acids). Dropout media to select for plasmid maintenance was prepared by leaving out the specific nutrient, and plasmids are listed in S4 Table. Specific inhibitors (rapamycin, nicotinamide (NAM), sodium butyrate (SB), or N-acetyl cysteine (NAC)) were added just before pouring plates. Cells were cultured in a shaker at 30°C and harvested during the mid-exponential phase (OD_600_ = 0.8-1.0). All yeast growth media and reagents were obtained from Research Products International, US Biologicals, Fisher Scientific, and Millipore Sigma. For spotting assays, the OD_600_ from overnight cultures was determined, and then an equivalent of OD_600_ = 1.0 cell number was serially diluted 1:5 before spotting onto the designated plates. Images were captured using a MultiDoc-It Digital Imaging System at the indicated time in days.

### Spot assay quantification

Spot assay quantification was performed using ImageJ, as described [89], with minor modifications as necessary. In brief, the image was imported into ImageJ, and the image type was set to 8-bit. The diameter of the largest spot in the spot assay was measured in pixels using the straight-line tool. The background was then subtracted by selecting the sliding paraboloid and setting the rolling ball diameter to the measured largest spot diameter + 20 pixels. Then, using the circular selection tool, we quantified the third dilution (25X) by selecting Analyze > Measure. To subtract the gray value of the spotting assay image background, the same circular selection was applied to five different locations on the plate. The quantified value of each spot was used to generate graphical data with GraphPad Prism.

### Site-directed mutagenesis

The *HHT2-HHF2* genetic locus was cloned into the 2µ plasmid pRS425, and this plasmid was then used as a template for site-directed mutagenesis. Briefly, primers were designed using NEBaseChanger to change H3K37 to H3K37A or H3K37R (see S5 Table for primer details). PCR using high-fidelity NEB Q5 enzyme was performed for each primer set to create mutations at H3K37A and H3K37R, and PCR products were stored at 4 °C. To remove the template plasmid, the PCR products were treated with NEB Kinase, Ligase, & DpnI (KLD) per the manufacturer’s instructions and incubated at room temperature for five minutes. After incubation, 5 μL of the KLD mix was transformed into JM109 competent cells and plated. Colonies were picked, plasmid was purified, and positive clones were identified by sequencing.

### RNA isolation

RNA was isolated using the hot acid phenol approach [90]. All RNAs were digested with RQ1 DNase (Promega) in a 50 μL total reaction volume incubated at 37 °C for 45 minutes. RNA was purified by phenol/chloroform extraction, precipitated, and then resuspended in 80 µL of nuclease-free water. RNA concentrations were determined by Nanodrop analysis and then stored at −70 °C.

### RNA sequencing

RNA libraries were created using the Qiagen FastSelect RNA kit, and the libraries were quantified by Qubit and Qiaxcel RNA size selection. Libraries were then sequenced on Illumina NextSeq 2000 using 2x50 P3 chip, and 20 million reads per sample were generated. Each fastq was subjected to quality control and correction using FASTQC and FASTX trimmer. The trimmed fastq was aligned to the S288 reference genome using RNA-STAR. The SAM files were then mined for the alignment of primary counts using feature Counts. The counts are normalized using TMM normalization and Log2 transformed. The transformed data matrix was loaded into R to gather statistics and determine differential expression. The mean, variance, standard deviation, and standard error of means were calculated for each gene across each condition. A principal component analysis was performed to determine different clusters in the samples. Limma with a linear regression model was used to identify genes with log2 fold change >= 0.58 and p value < 0.05. P values were then adjusted for multiplicity using the Benjamini-Hochberg method. Only genes with an adjusted p-value < 0.05 were considered differentially expressed. These genes were used to create a heatmap with unsupervised hierarchical clustering. The gene list was loaded into String DB for pathway and functional analysis. Sequencing data have been deposited at the National Institutes of Health Gene Expression Omnibus (GEO) repository with accession number GSE312461.

### cDNA synthesis and real-time quantitative PCR (RT-qPCR)

Briefly, 2 µg of template RNA was reverse transcribed in a final volume of 20 µL using the ImProm-II Reverse Transcription System from Promega per the manufacturer’s protocol. The total 20 µL reaction mix was incubated at 25 °C for five minutes, shifted to 42 °C for 60 minutes, and then inactivated by incubating at 70°C for 15 minutes. The resulting cDNA was diluted 1:10 before performing qPCR using PowerUp SYBR Green Master Mix according to the manufacturer’s protocol. The cycle threshold (Ct) at which the fluorescence intensity crossed the threshold was used to compare samples. Target genes were normalized to the signal of the endogenous control gene *ACT1* (S5 Table for primer details). Results were expressed as fold changes in relative expression. All statistical analyses and graphs were generated using GraphPad Prism 10.

### Chromatin immunoprecipitation RT-qPCR (ChIP-qPCR)

Chromatin immunoprecipitation qRT-PCR (ChIP-qPCR) was performed as previously described with minor modifications [91]. Briefly, H3WT and H3K37A cells were grown to mid-log phase and cross-linked with 1% formaldehyde for 15 minutes, followed by quenching for 5 minutes with 125 mM glycine at RT. Cells were lysed in FA lysis buffer containing 50 mM HEPES-KOH (pH 7.5), 300 mM NaCl, 1 mM EDTA, 1% Triton X-100, and 0.1% sodium deoxycholate supplemented with protease and phosphatase inhibitors. Chromatin was fragmented by Bioruptor sonicator for ~7 minutes of sonication at high power with 30 sec on/off and clarified by centrifugation. Equal amounts of chromatin were incubated with the indicated antibody, and immune complexes were captured using Protein A agarose beads. Beads were sequentially washed with 300 mM and 500 mM FA lysis buffers (50 mM HEPES-KOH (pH 7.5), 500 mM NaCl, 1 mM EDTA, 1% Triton X-100, and 0.1% sodium deoxycholate), LiCl wash buffer (250 mM LiCl, 0.5% NP-40, 0.5% sodium deoxycholate, and 1 mM EDTA), and 1X TE buffer. Chromatin was eluted in 0.1 M sodium bicarbonate containing 1% SDS, and cross-links were reversed in the presence of 2.5 M NaCl. DNA was purified using silica membrane columns and analyzed by RT-qPCR for the indicated primers, and the fragment enrichment was normalized to the corresponding input DNA.

### Immunoblot (IB) analysis

Total protein was isolated by bead beating from 50 mL cultures at OD_600_ = 0.8 - 1.0 that were lysed in 120 µL of lysis buffer (10% glycerol, 10 mM Tris (pH 8.0), 0.1% NP40, 300 mM NaCl) with freshly added protease inhibitors (2 µg/mL leupeptin, 2 µg/mL pepstatin, 2 µg/mL aprotinin, 1 mM Dithiothreitol (DTT), 1x protease inhibitor cocktail and 1 mM PMSF). Protein concentration was determined by Bradford assay, samples (40 µg) were prepared in 2X sample buffer and resolved by either 10% or 12% SDS-PAGE, and then gels were transferred to PVDF membrane. IBs were probed with primary antibody to the following proteins and/or modified proteins: H3 (cat# 39763), H4 (cat# 39269), H2A (cat# 39235), H3K36me3 (cat# 61101), and H3K23Ac (cat#38131) from Active Motif; G6PDH (cat# A9521) and FLAG-M2 (cat# F1804) from Sigma-Aldrich; RPS6/S6 (cat# ab40820) and Tubulin (cat# ab184970) from Abcam; phosphoS6 (Ser235/236) (cat# 22115) from Cell Signaling; HA (cat# SC805) from Santa Cruz; H3K36me2 [48]. Primary antibodies were diluted in 5% milk/TBST buffer, incubated at 4 °C overnight with rocking, and then the blot was washed three times with 1X TBST. Membranes then were incubated with HRP-conjugated secondary antibody (mouse anti-rabbit peroxidase-conjugated IgG (cat# 211-032-171) or goat anti-mouse peroxidase-conjugated IgG (cat# 115-035-174) from Jackson Immuno Research), washed extensively, and then IBs were developed using the Immobilon Western Chemiluminescent HRP Substrate system. IBs were analyzed with ImageJ, and the band intensity data were used to generate graphical data using GraphPad Prism.

### Cell death analysis

Cell death analysis was performed using propidium iodide (PI) and YO-PRO-1 (YO) as described previously [21]. Briefly, cells were grown in SC media overnight to an OD_600_ = 0.6-0.8 and then either mock-treated or treated with 50 nM rapamycin for six hours before harvesting. An OD_600_ = 1.0 cells were pelleted, washed with cold PBS, and resuspended in 1 mL cold PBS. To the cell suspension 5 µg/mL of PI and 5 µM of YO were added, mixed well, and incubated at room temperature for 20 minutes. Subsequently, stained cell samples were recorded on a Bio-Rad ZE5 flow cytometer using 488 nm excitation, and data were collected from either a 530/30 bandpass nM green fluorescence emission for YO, or 610/20 bandpass nM red fluorescence emission for PI. Gating on cells was performed to exclude debris using single-color stained controls with standard compensation, and cell populations were categorized into three groups: live cells with low green fluorescence, apoptotic cells with high green fluorescence, and dead cells with both red and green fluorescence.

### Mitochondrial membrane potential (MMP) analysis

The MMP analysis was conducted using tetramethyl rhodamine ethyl ester (TMRE) and MitoTracker Green (MTG) as previously described with minor modifications [92]. Cells were grown in SC media overnight to an OD_600_ = 0.6-0.8, mock-treated or treated with 20 nM rapamycin, and after 30 minutes of treatment, TMRE and MTG were added and incubated at 30 °C for a further 30 minutes. The uncoupler carbonyl cyanide m-chlorophenylhydrazone (CCCP) was added 10 minutes before TMRE and MTG, and it was used as the positive control. After a total of one hour rapamycin treatment and staining, OD_600_ = 1.0 cells were pelleted, washed twice with PBS, and collected by centrifugation. Cell pellets were resuspended in 500 µl PBS, and samples were analyzed on a Bio-Rad ZE5 flow cytometer using a 530/30 bandpass filter for MTG and a 585/40 bandpass filter for TMRE.

### Cell cycle analysis

Cells were grown in SC medium overnight to an OD_600_ = 0.8-1.0, and cell cycle analysis was performed as previously described [93,94]. Briefly, 1.5 mL culture was transferred into a 15 mL centrifuge tube and pelleted at 3000xg for five minutes. Cell pellets were washed once with 1 mL cold water, pelleted again at 3000xg for five minutes, and then pellets were resuspended in 1.5 mL cold MilliQ water. To the cell suspension 3.5 mL of ice-cold ethanol (100%) was added dropwise while vortexing to prevent clumping, and then the samples were either incubated at room temperature for one hour or overnight at 4 °C. Cells were pelleted at 3000xg for five minutes, and pellets were washed twice with 1 mL ice-cold sodium citrate buffer (50 mM sodium citrate, pH 7.2 adjusted with citric acid). Pellets were resuspended in 300 µL of RNase A solution (2 mg/mL of RNase A in 50 mM Tris (pH 8) and 15 mM NaCl) and then incubated at 37 °C for four hours with periodic mixing before centrifuging at 3000xg for five minutes. Pellets then were resuspended in 500 µL of freshly prepared protease solution (5 mg/mL pepsin in water with 4.5 µL/mL concentrated HCl added), incubated at 37 °C for 20 minutes, and then centrifuged at 3000xg for five minutes. Cell pellets were resuspended in 500 µL of ice-cold sodium citrate buffer and either kept on ice or at 4 °C until staining. For staining, 50 µL of a fresh 1 mL SYTOX solution (1 µM SYTOX in sodium citrate buffer) was added to the cell suspension, cells were incubated at room temperature for 10 minutes, and then they were transferred to ice. Samples were sonicated for 15 seconds at low power (20% amplitude) while on ice, and then they were analyzed on a Bio-Rad ZE5 flow cytometer using FlowJo software as previously described [94].

### MNase assay

The MNase assay was performed as described with minor modifications [42,95]. Cells grown in 100 mL SC media overnight were collected at an OD_600_ = 0.8-1.0, washed with 1.5 mL of cold water, and then pelleted before either continuing the protocol or freezing pellets at -80 °C. Cell pellet were washed in cold PSB buffer (20 mM Tris-HCl (pH 7.4), 100 mM NaCl, and 10 mM 2-β-mercaptoethanol (2-βME)), followed by cold ST buffer (1 M sorbitol and 20 mM Tris-HCl (pH 7.4)), and then resuspended in 1.5 mL of cold ST buffer. The initial OD_600_ was measured, and then 10 units/mL of Zymolyase 20T in 1 mM DTT was added to the cell suspension and samples were incubated at 30 °C for 30 minutes. The OD_600_ was measured and once the OD_600_ was less than 80% of the initial starting OD_600_, fresh ST buffer was added. Spheroplasts then were pelleted by centrifuging at 7500 rpm for five minutes at 4 °C, washed with 1.5 mL of cold ST buffer, and then pelleted again at 7500 rpm for five minutes at 4 °C. Spheroplasts were resuspended in 800 µL of F-buffer, pH 6.5 (18% Ficoll-400, 40 mM K-phosphate monobasic (pH 7.0), 1 mM MgCl_2_), to which 5 mM 2-βME and protease inhibitors (10 µg/mL leupeptin, 1 µg/mL pepstatin, 1 µg/mL aprotinin, 1x protease inhibitor cocktail and 1 mM PMSF) were freshly added. Samples were incubated on ice for five minutes before layering 600 µL FG-buffer pH 6.5 (7% Ficoll-400, 20% glycerol, 40 mM K-phosphate monobasic (pH 7.0), 1 mM MgCl_2,_ and freshly added 5 mM 2-βME and protease inhibitors) on top of the samples and centrifuging at 12,500 rpm for 20 minutes at 4 °C. The supernatant was discarded and the nuclear pellet placed on ice. Nuclei were resuspended in 500 µL of MNase digestion buffer (10 mM HEPES (pH 7.5), 35 mM NaCl, 0.5 mM MgCl_2_, and 0.5 mM CaCl_2_), divided into 5 aliquots, and then MNase was added at the following concentrations: 0 U (undigested), 2.5 U, 5 U, 7.5 U, and 10 U. Samples were incubated at 37 °C for 10 minutes before adding 12.5 µL of MNase stop solution (65 mM EDTA, 7% SDS). To each sample, 20 µL of 2 mg/mL RNase A was added, incubated at 37 °C for one hour, and then 20 µL of 10% SDS and ~1 M of potassium acetate was added before performing phenol/chloroform extraction. DNA was precipitated, the pellet was resuspended in 20 µL of water and then visualized by resolving on a 2% agarose gel prestained with Midori green safe stain.

### Reactive oxygen species (ROS) quantification

ROS levels were quantified by DHE staining as previously described [96]. Cells were grown in SC media overnight before mock treating or treating with 50 nM rapamycin for 24 hours at an OD_600_ = 0.8-1.0. An equivalent of OD_600_ = 1.0 number of cells then was centrifuged, washed twice with PBS, and resuspended in 200 µL of staining buffer (0.1% glucose and 1 mM EDTA, pH 8.0, in PBS). Cells were transferred to a 96-well black flat-bottom microplate, and 30 µM of DHE was added to each well and incubated at 30 °C for 15 minutes in the dark. ROS levels were measured using a fluorescence plate reader (BioTek Cytation 5) with an excitation wavelength of 520 nm and an emission wavelength of 600 nm [97,98]. For the positive control, cells were treated with 10 mM hydrogen peroxide to increase ROS levels, while 50 mM NAC was used to confirm ROS quenching.

### Software and statistical analysis

All experiments were conducted with n = 3 or more independent biological replicates, and GraphPad Prism was used to calculate statistical significance. For comparing multiple groups, a one-way ANOVA with Bonferroni’s multiple comparisons test was performed, while comparing two groups was performed using an unpaired t-test. Results are presented as the mean ± standard deviation (SD) with a 95% confidence interval, and a p-value of <0.05 was considered significant. To represent the significance, we used (*) for a one-way ANOVA and (^#^) for t-test. Hypergeometric testing was used to test significance of transcriptome overlap for the data represented in S6E Fig using the online software (https://flaski.age.mpg.de/venndiagram/).

## Supporting information

S1 Fig Characterization of histones in the H3K37A mutant. **(A)** The H3WT and H3K37A cells were transformed with either control vector, or a vector expressing all four core histones, and then 5-fold serially diluted and spotted to the indicated plates for 3–8 days. **(B)** Whole cell extracts from cells in **A** were analyzed by IB for histone expression. **(C-E)** Quantification of histones H3, H4, and H2A normalized to α-tubulin. n = 3–4; *p < 0.05, ****p < 0.0001. **(F-I)** RT-qPCR analyses were performed for each of the indicated histone genes. n = 3–4; ns – no significance.
(TIF)

S2 Fig Analysis of the cell cycle and global chromatin accessibility of the H3K37A mutant. **(A-B)** H3WT and H3K37A were grown to mid-exponential phase, stained with SYTOX Green, and analyzed by flow cytometry. The histogram displays the SYTOX green fluorescence intensity (DNA content) on the x-axis and cell count on the y-axis. Data are representative of the cell cycle results represented in Fig 2A. **(C)** H3WT and H3K37A were treated with 2.5U, 5U, 7.5U, and 10U of MNase for 10 minutes at 37 °C before resolving samples on a 2% agarose gel. Lane 1 – 100 base pair (bp) DNA ladder, Lane 2 – 6 H3WT, Lane 7 empty, Lane 8 – 12 H3K37A. UD – Undigested.
(TIF)

S3 Fig A *set2∆* causes a synthetic lethal TORC1 phenotype in the H3K37A mutant. **(A)** The dual fluorescence scatter plot flow cytometry analysis of H3WT, H3K37A, and their *set2Δ* derivatives cells that were mock or 50 nM Rap-treated for 6 hours. YO-PRO-1 (YO) on the X-axis and propidium iodide (PI) on the Y-axis. Data are representative for the cell death results in Fig 3B. **(B)** IB analysis of histone H3 in H3WT, H3K37A, and their *dot1Δ* derivatives. **(C)** Quantification of histone H3 normalized to G6PDH from **B**. n = 5; **p < 0.01 and ns – no significance. **(D)** H3WT and H3WT *set2Δ* were transformed with the control vector, or with the vector expressing wild-type Set2 or the indicated Set2 catalytic mutants (in H3WT *set2Δ*), and H3K36me2, H3K36me3, and total H3 were analyzed by IB. **(E)** IB analysis of histone H3K23Ac and total H3 in H3WT, H3K37A, and their *set2Δ* derivatives. **(F)** Quantification of **E**. n = 3; *p < 0.05, **p < 0.01 and ^*#*^*p < 0.05*.
(TIF)

S4 Fig Principal component analysis (PCA) of the RNA-seq independent replicates. **(A)** H3WT (mock vs rapamycin), **(B)** H3WT vs H3K37A (mock), **(C)** H3WT mock vs H3K37A rapamycin, **(D)** H3K37A (mock vs rapamycin). **(E)** H3WT rapamycin vs H3K37A rapamycin. **(F)** Heatmap analysis of H3WT rapamycin vs H3K37A rapamycin.
(TIF)

S5 Fig Heatmap analysis of the RNA-seq independent replicates. **(A)** H3WT (mock vs rapamycin), **(B)** H3WT vs H3K37A (mock), **(C)** H3WT mock vs H3K37A rapamycin, **(D)** H3K37A (mock vs rapamycin).
(TIF)

S6 Fig Bubble blot analysis of the cellular component gene ontology (GO) categories. **(A)** H3WT (mock vs rapamycin), **(B)** H3WT vs H3K37A (mock), **(C)** H3WT mock vs H3K37A rapamycin, **(D)** H3WT rapamycin vs H3K37A rapamycin. GO analysis was performed through the STRING database. FDR – False discovery rate. **(E)** Venn analysis of differentially expressed genes (DEGs) from H3WT vs H3K37A (current study), WT vs *set2∆* (McDaniel *et al.,* 2017), and WT vs *set2∆* (Li *et al.,* 2023). Hypergeometric testing was performed to analyze significance using a population size of 6275 total genes.
(TIF)

S7 Fig RT-qPCR validation of histone gene expression. **(A-F)** RT-qPCR analysis for the indicated histone genes. n = 3; *p < 0.05, ns – no significance. **(G)** The H3WT, H3K37A, and the *hho1Δ* derivatives of each were serially diluted 5-fold and spotted to the indicated plates for four days.
(TIF)

S8 Fig Characterization of the retrograde pathway in the H3K37A mutation. **(A-D)** RT-qPCR analysis of the indicated mitochondrial genes from the H3K37A+rapamycin sample as identified by RNA-seq. n = 6; ****p < 0.0001 and ^*##*^*p < 0.01*, ns – no significance. **(E-F)** RT-qPCR analysis of the overexpressed mitochondrial genes (C) and (D) validated in the H3WT, H3K37A, and their *set2∆* derivatives samples. n = 3; *p < 0.05, **p < 0.01, ***p < 0.001, ****p < 0.0001, and ^*#*^*p < 0.05.* **(G-H)** The graphical data of log2-normalized counts for *CIT1* and *CIT2* from the RNA-seq analysis. n = 3 – 4; *p < 0.05, **p < 0.01, ***p < 0.001, ****p < 0.0001, ns – no significance. **(I)** RT-qPCR analysis of *CIT1* to validate that *rtg1∆* suppresses retrograde activation in H3WT and H3K37A. n = 3; *p < 0.05, **p < 0.01, ****p < 0.0001 and ^*#*^*p < 0.05*. **(J)** RT-qPCR analysis of *CIT2* to validate that *rtg1∆* suppresses retrograde activation in H3WT and H3K37A. n = 3; *p < 0.05, **p < 0.01, ****p < 0.0001. ns – no significance.
(TIF)

S9 Fig The H3K37A mutation deregulates mitochondrial function. **(A)** The indicated strains from Fig 7B were cultured to exponential phase and stained with DHE before quantifying DHE fluorescence. A.U.– Arbitrary Units of DHE fluorescence. n = 7; ns– no significance. **(B)** As in **A**, except the cells were treated with 50 nM rapamycin for 24 hours before staining with DHE. n = 8; **p < 0.01, ***p < 0.001, ****p < 0.0001 and ^*#*^*p < 0.05*, ns– no significance. **(S)** The dual fluorescence scatter plot flow cytometry analysis of H3WT and H3K37A was mock or 20 nM Rap-treated for 1 hour. TMRE on the Y-axis and MitoTracker Green (MTG) on the X-axis. The data are representative of the mitochondrial depolarization shown in Fig 7C. No stain is the negative control. A single stain was used for compensation control, and CCCP with TMRE and MTG staining was used for a positive control.
(TIF)

S10 Fig Characterization of the histone instability mutants in H3WT. **(A)** RT-qPCR analysis of *CIT2* to analyze retrograde activation the histone instability mutants in H3WT. n = 3; **p < 0.01, and ^*#*^*p < 0.05,* ^*##*^*p < 0.01*. (**B**) TMRE and MitoTracker Green staining of the histone instability mutants in H3WT and analysis by flow cytometry. CCCP is an uncoupler used as a positive control. n = 3; *p < 0.05, and ^*#*^*p < 0.05,* ns – no significance**. (C)** The dual fluorescence scatter plot data are representative of the mitochondrial depolarization shown in **B**. TMRE on the Y-axis and MitoTracker Green (MTG) on the X-axis. No stain is the negative control. A single stain was used for compensation control, and CCCP with TMRE and MTG staining was used for positive control.
(TIF)

S11 Fig MMP analysis of *dot1∆* in H3WT and H3K37A. **(A)** The H3WT, H3K37A, and their *dot1∆* derivatives were mock-treated or treated with 20 nM rapamycin for one hour, and then they were stained with TMRE and MitoTracker Green and analyzed by flow cytometry. CCCP is an uncoupler used as a positive control. n = 3; *p < 0.05, **p < 0.01,***p < 0.001 and ^*#*^*p < 0.05,* ^*##*^*p < 0.01,* ^*###*^*p < 0.001,* ns – no significance**. (B)** The dual fluorescence scatter plot data are representative of the mitochondrial depolarization shown in A. TMRE on the Y-axis and MitoTracker Green (MTG) on the X-axis. No stain is the negative control. A single stain was used for compensation control, and CCCP with TMRE and MTG staining was used for positive control.
(TIF)

S12 Fig Characterization of Cox4 protein expression in H3K37A. **(A)** RT-qPCR analysis of *COX4* expression. n = 3; ns – no significance. **(B)** IB analysis of Cox4-HA assesses mitochondrial protein import in control H3WT and H3K37A. P- precursor protein and M- mature protein. CCCP is an uncoupler used as a positive control, and G6PDH is used as a loading control. **(C)** Quantification of precursor Cox4-to-G6PDH ratio. n = 4; ns – no significance. **(D)** Quantification of mature Cox4-to-G6PDH ratio. n = 4; ***p < 0.001. **(E)** H3WT was either mock-treated or treated with 20 nM rapamycin, 50 mM NAC, or both inhibitors for one hour before analyzing TORC1 activity by S6ph/S6 IB.
(TIF)

S1 Rawgel Uncropped images of all immunoblots. The red boxes in each panel are the representative bands show for each immunoblot. The loading order for all blots is from left to right.
(PDF)

S1 Table RNA-sequencing data and differentially expressed gene lists. (XLSX)

S2 Table Cellular component gene ontology (GO) results. (XLSX)

S3 Table List of yeast strains. (DOCX)

S4 Table List of plasmids. (DOCX)

S5 Table List of primers. (DOCX)
