# Supplementary material for: Chromatin stability safeguards mitochondrial homeostasis and restrains TORC1 signaling
Source: PLoS Genet. 2026 Oct 8;22(10):e1012330. doi: 10.1371/journal.pgen.1012330 (PMC13667920; doi:10.1371/journal.pgen.1012330)

S1_raw_images

- This document contains the uncropped images of the immunoblots shown in the manuscript entitled "Chromatin stability safeguards mitochondrial homeostasis and restrains TORC1 signaling" by Sigamani et al.
- The representative image panel used in the manuscript is highlighted by red boxes in each raw image.
- All the blots shown in this document were developed using chemiluminescence, and the films were then scanned to create a soft copy.
- The lane loading order for the immunoblot shown in this manuscript is from left to right.

Figure 1B

H3

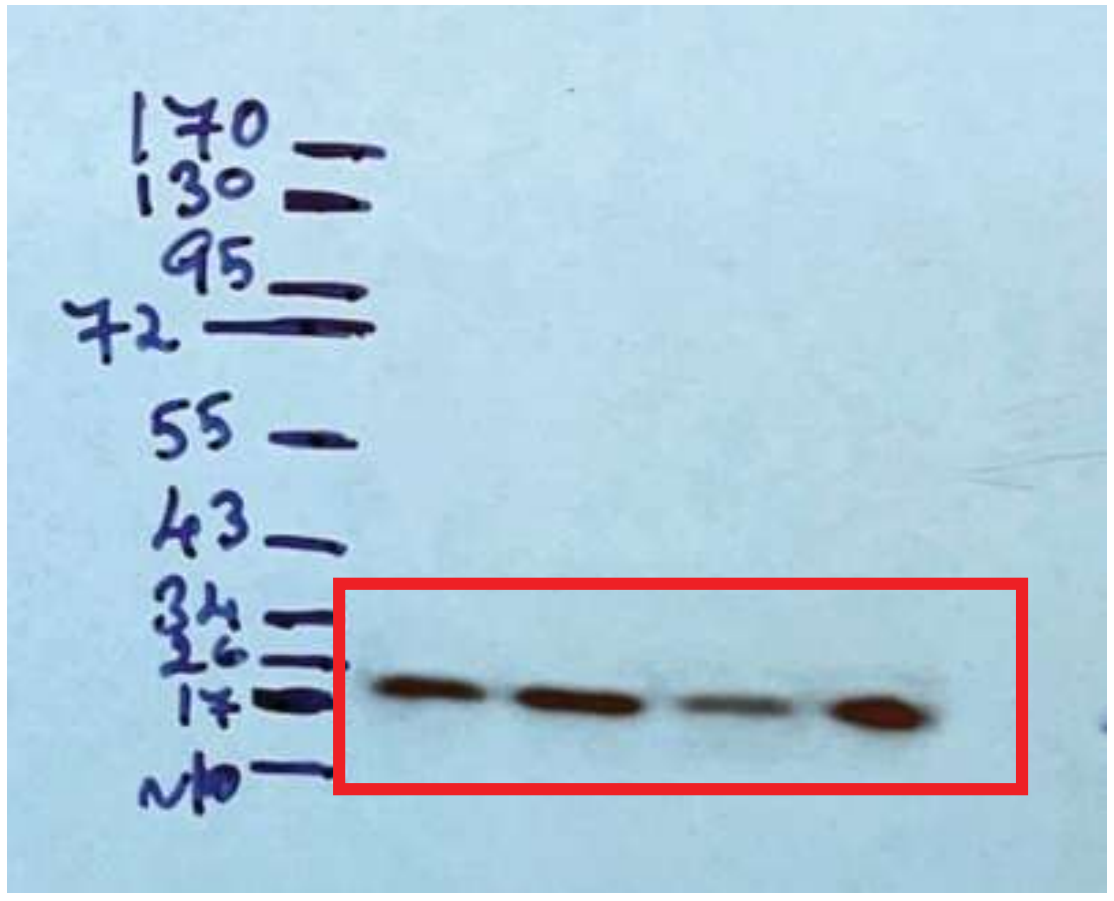

G6PDH for H3

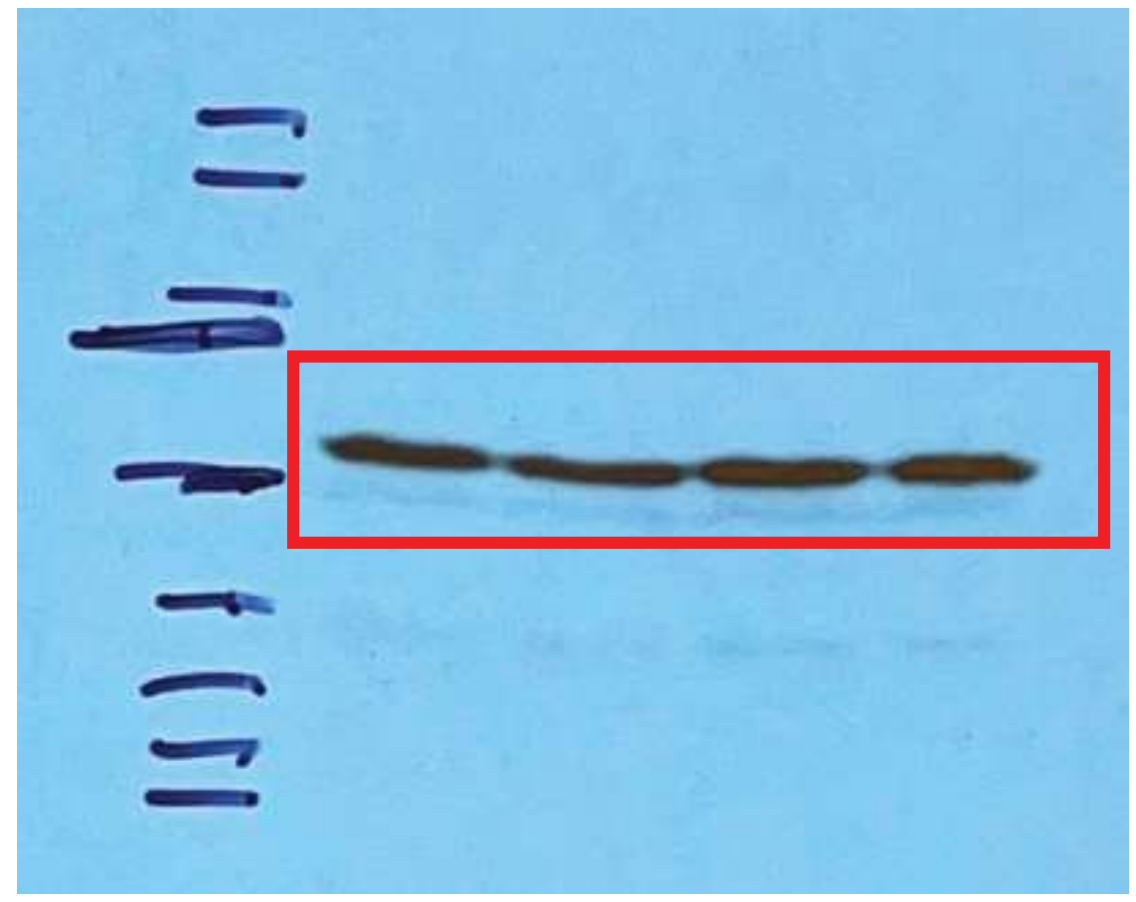

H2A

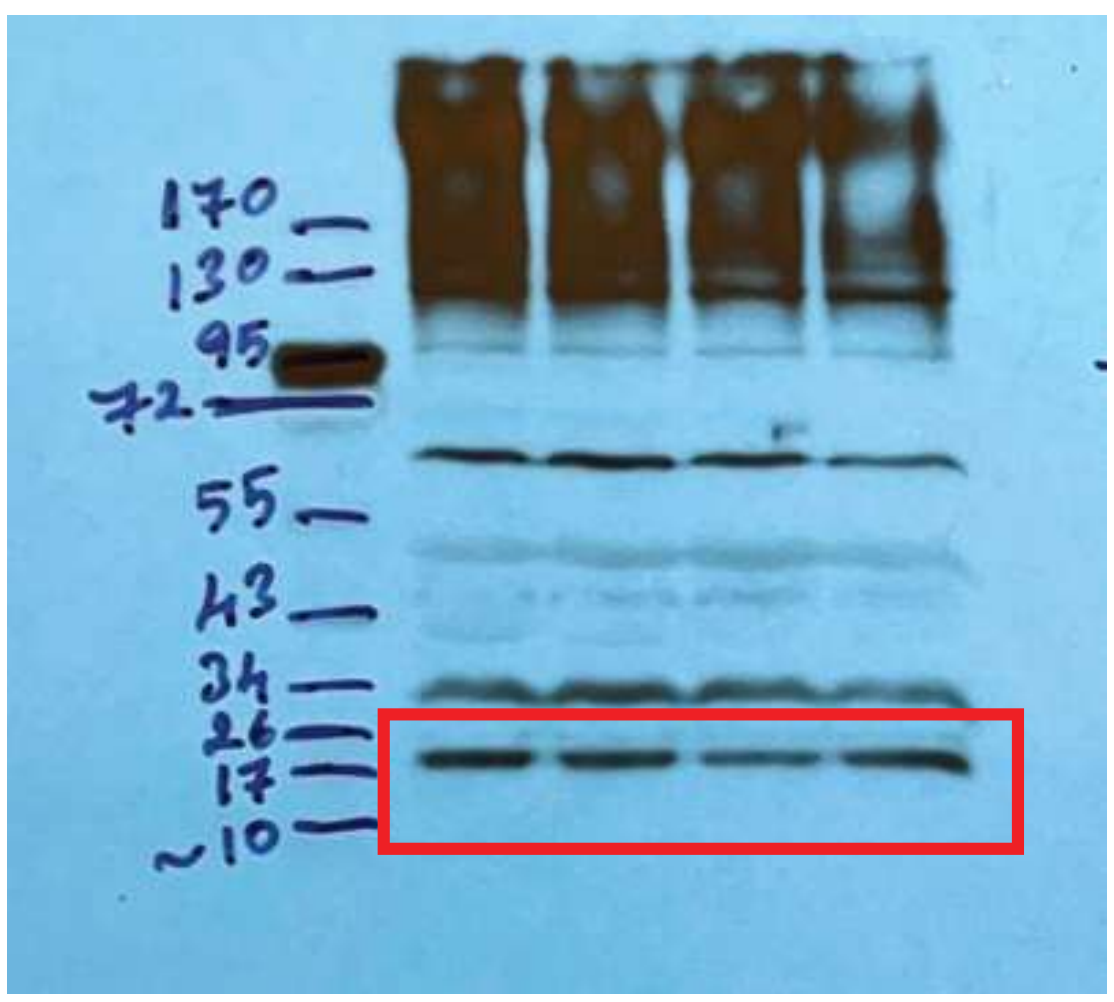

G6PDH for H2A

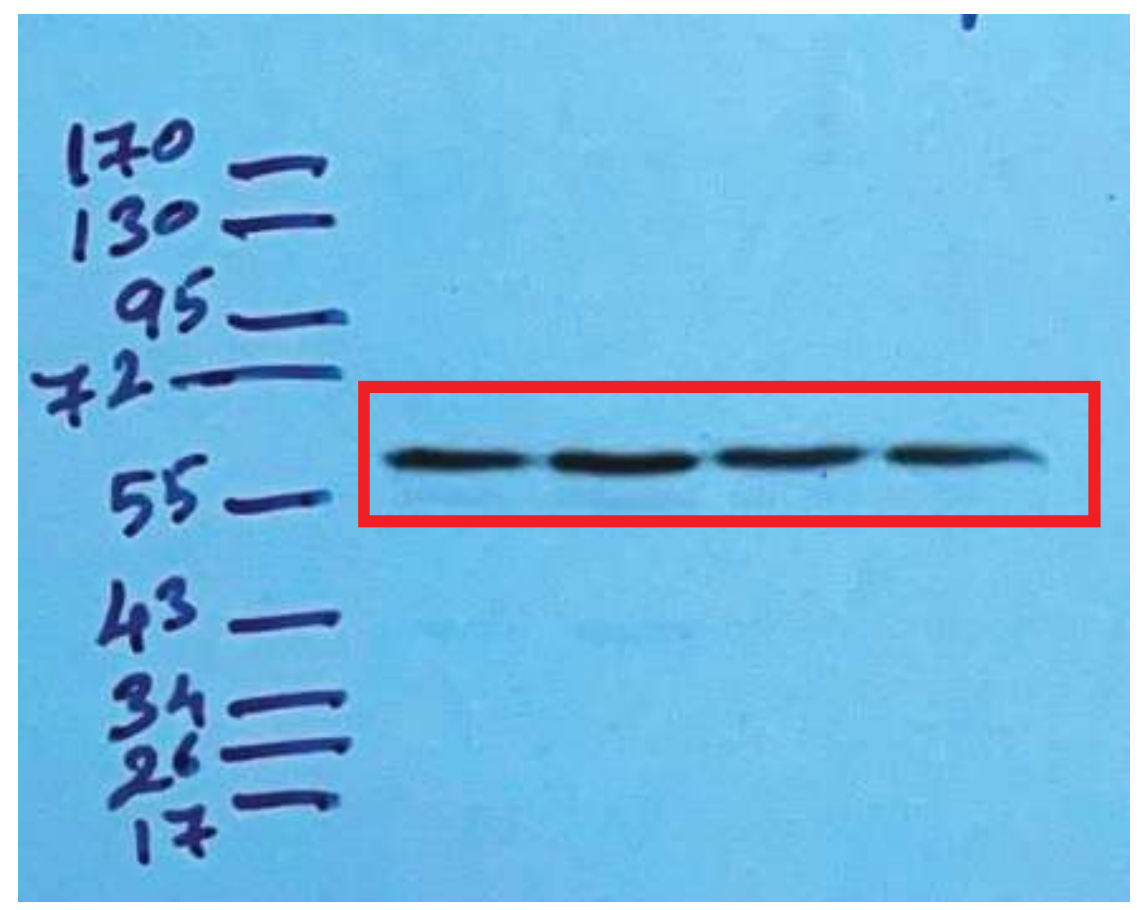

H4

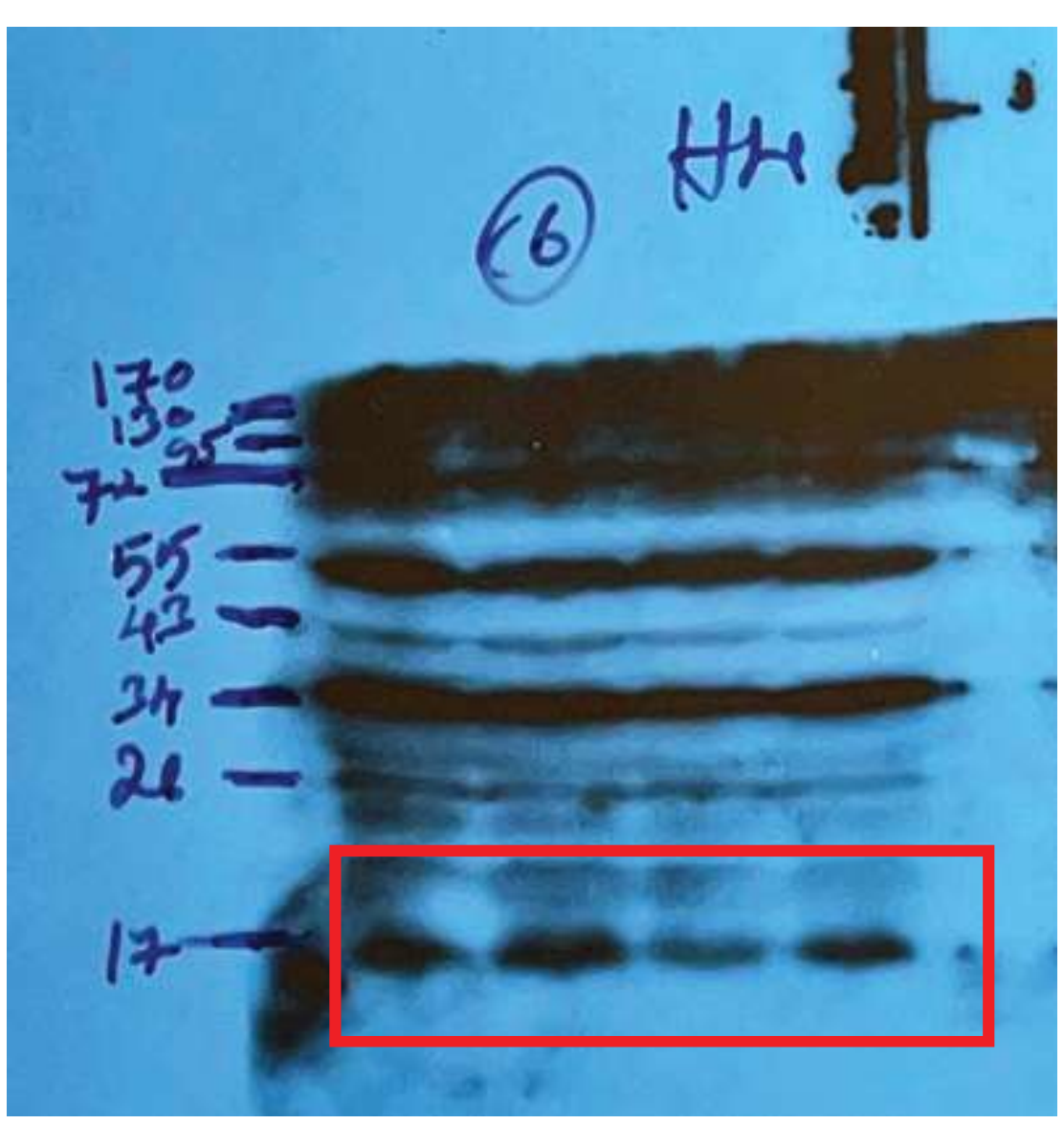

G6PDH for H4

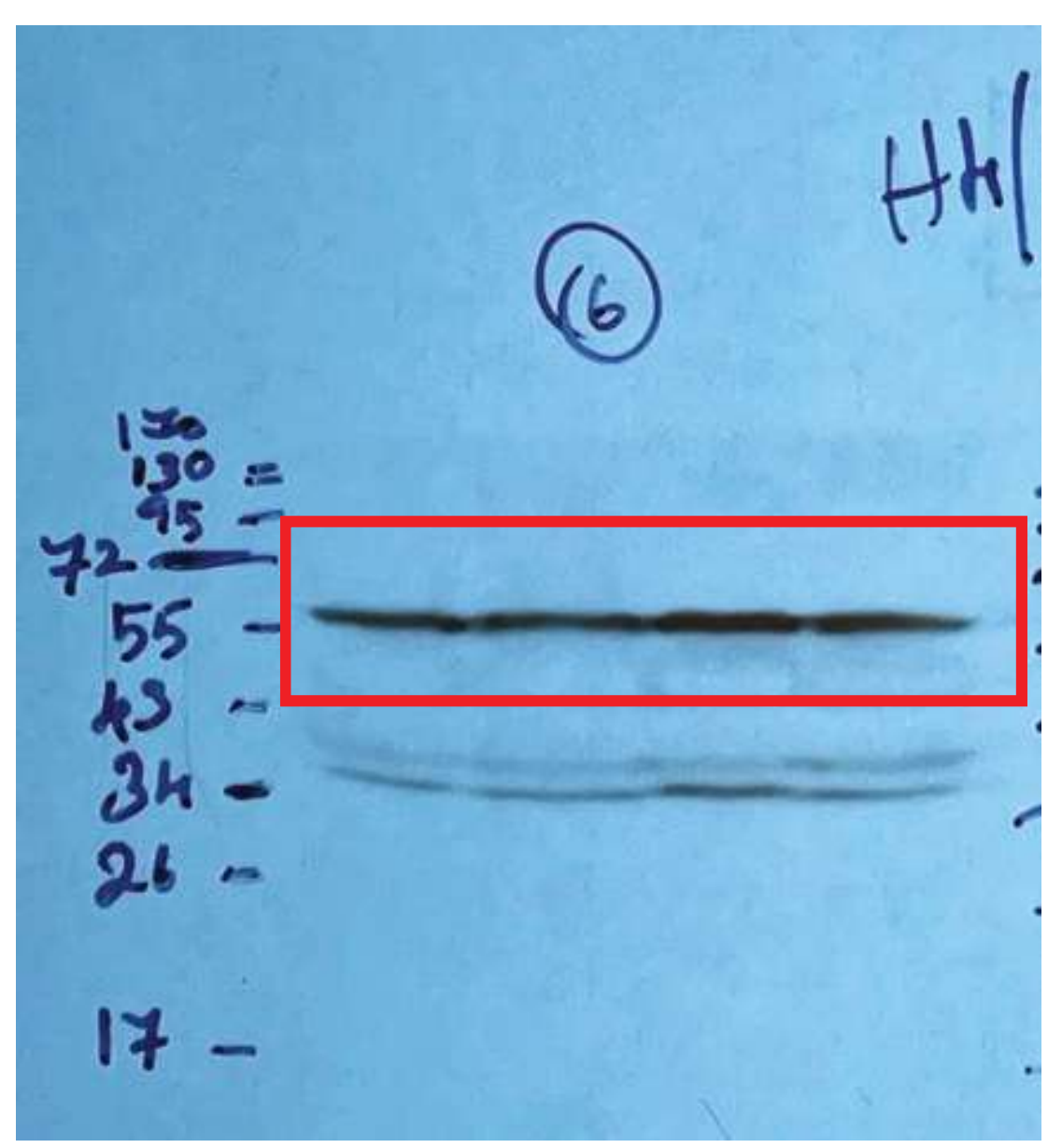

Figure 1J

H3

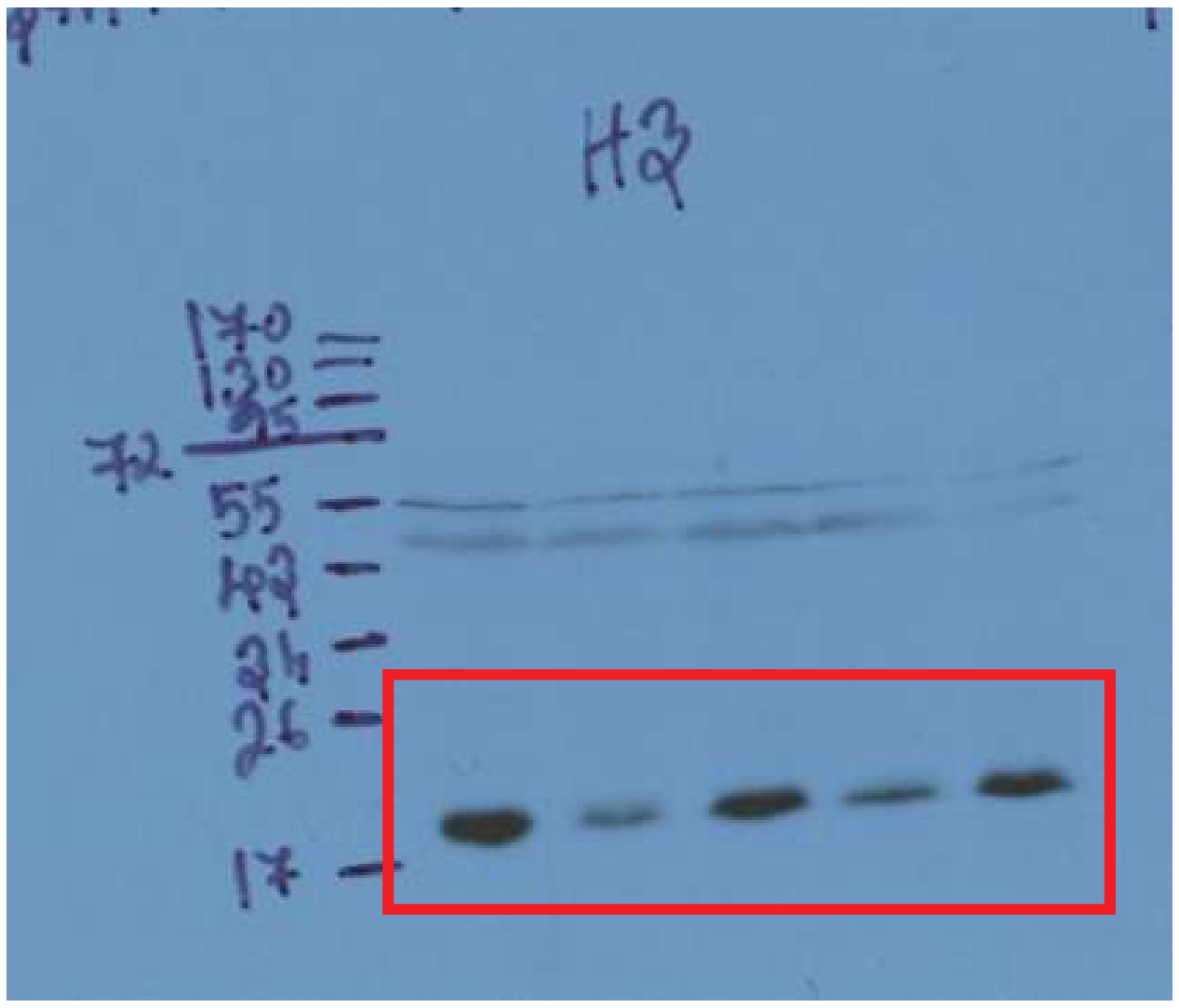

G6PDH for H3

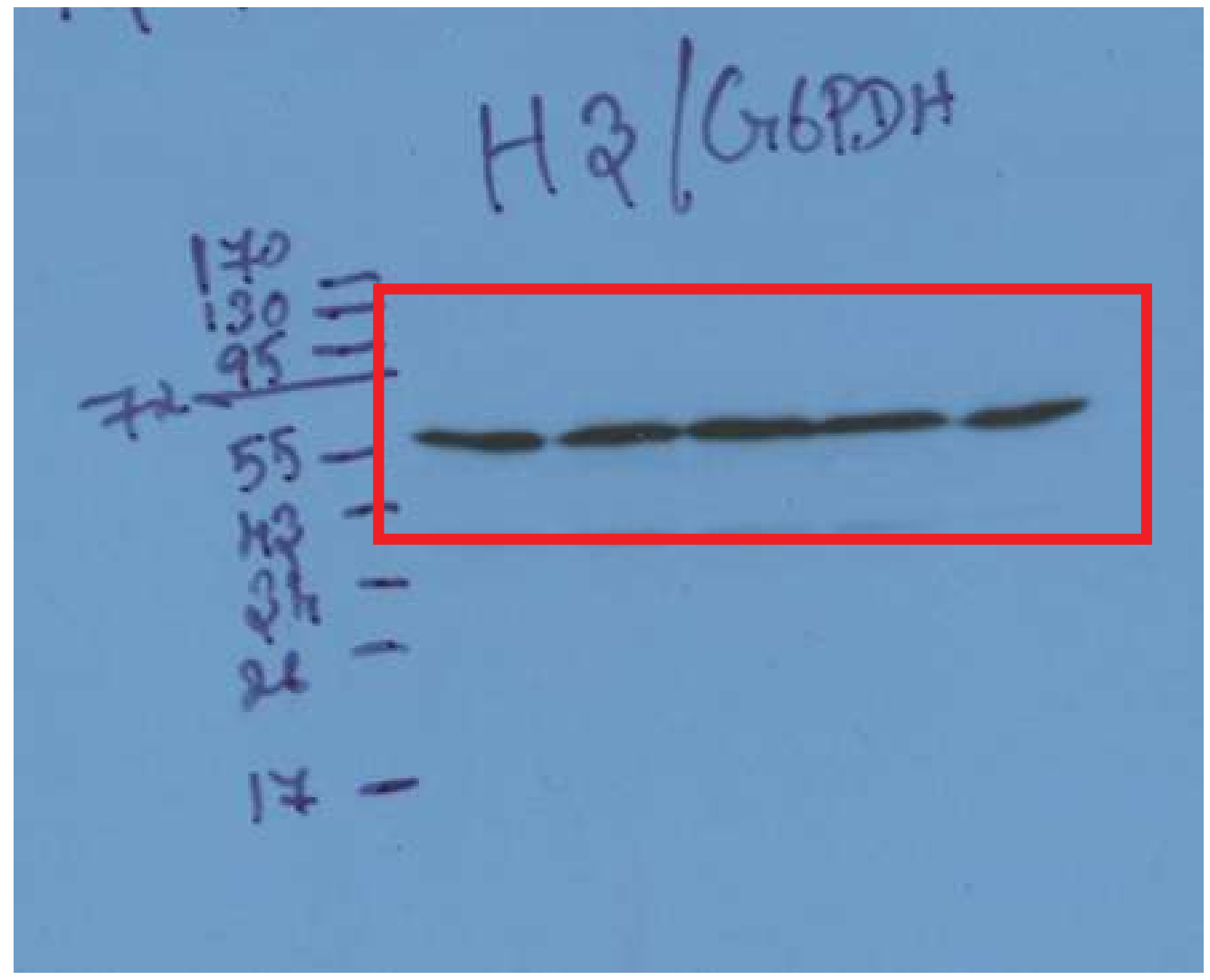

H4

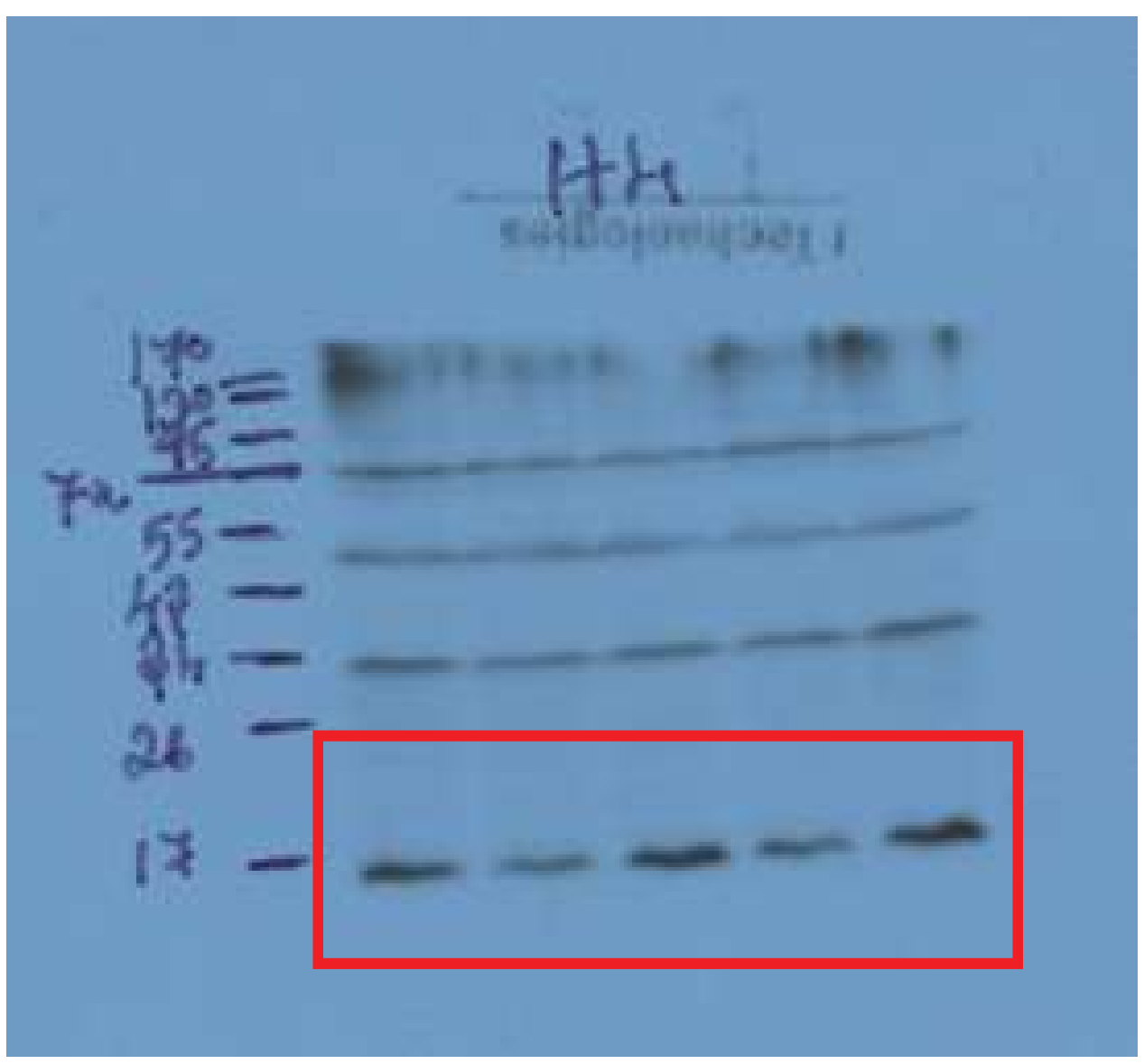

G6PDH for H4

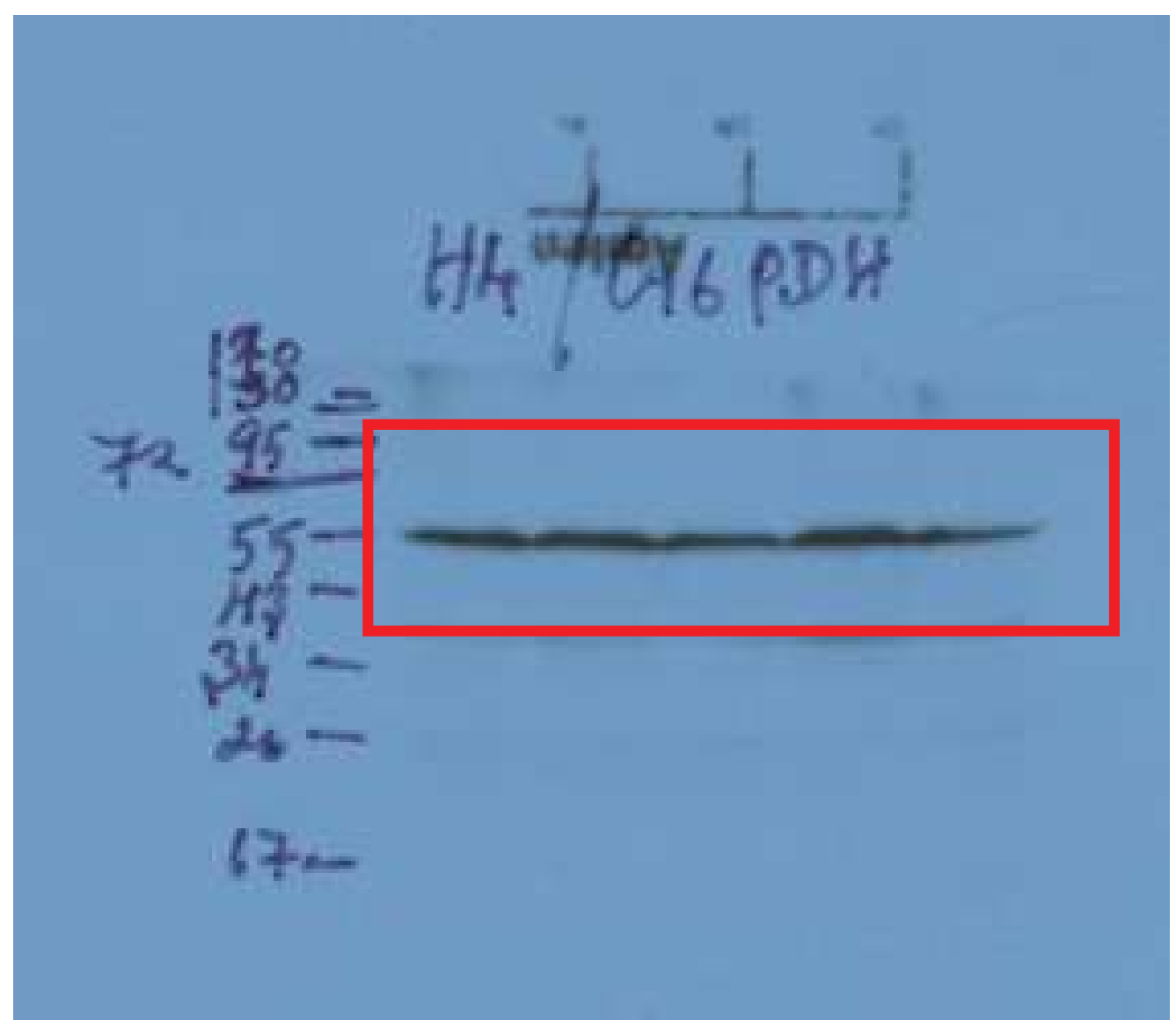

Figure 2B

H3

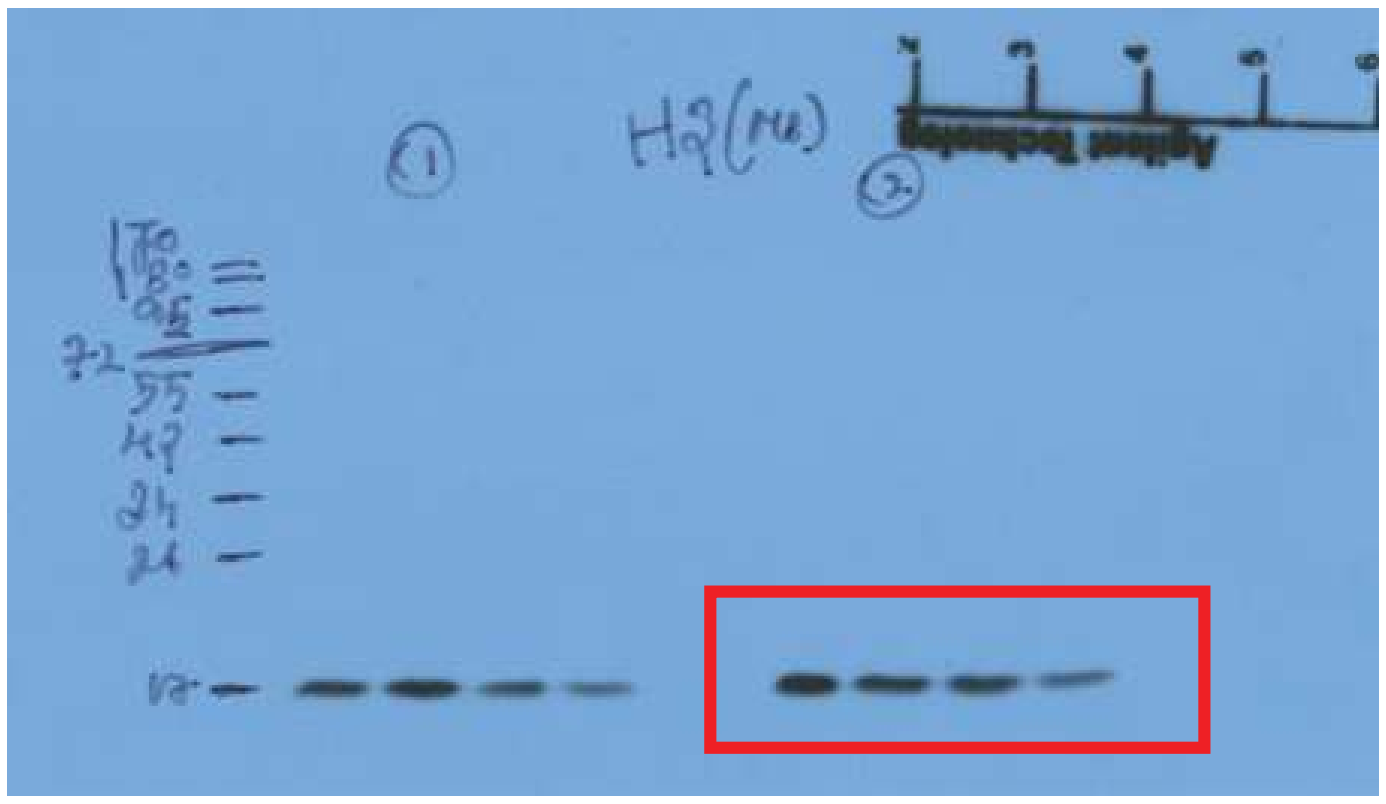

G6PDH for H3

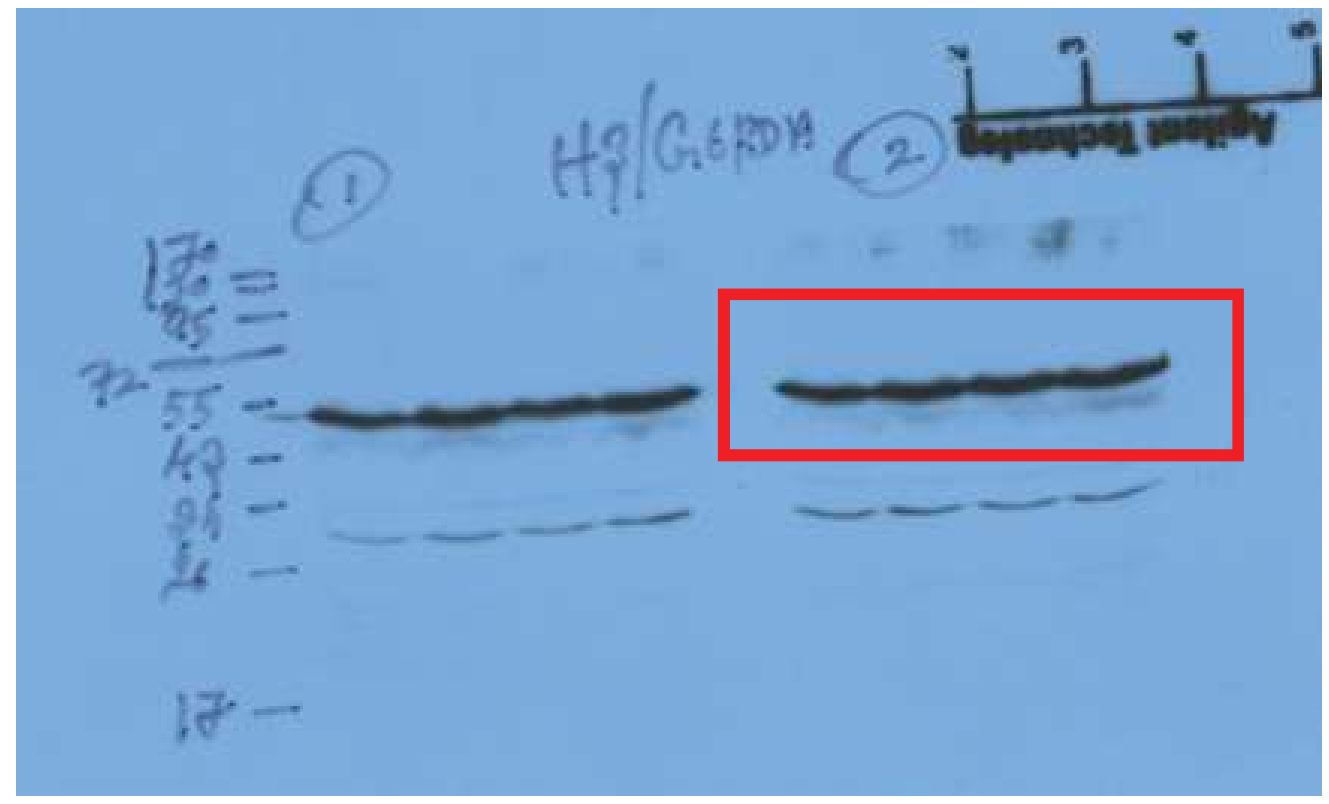

Figure 2D

H3

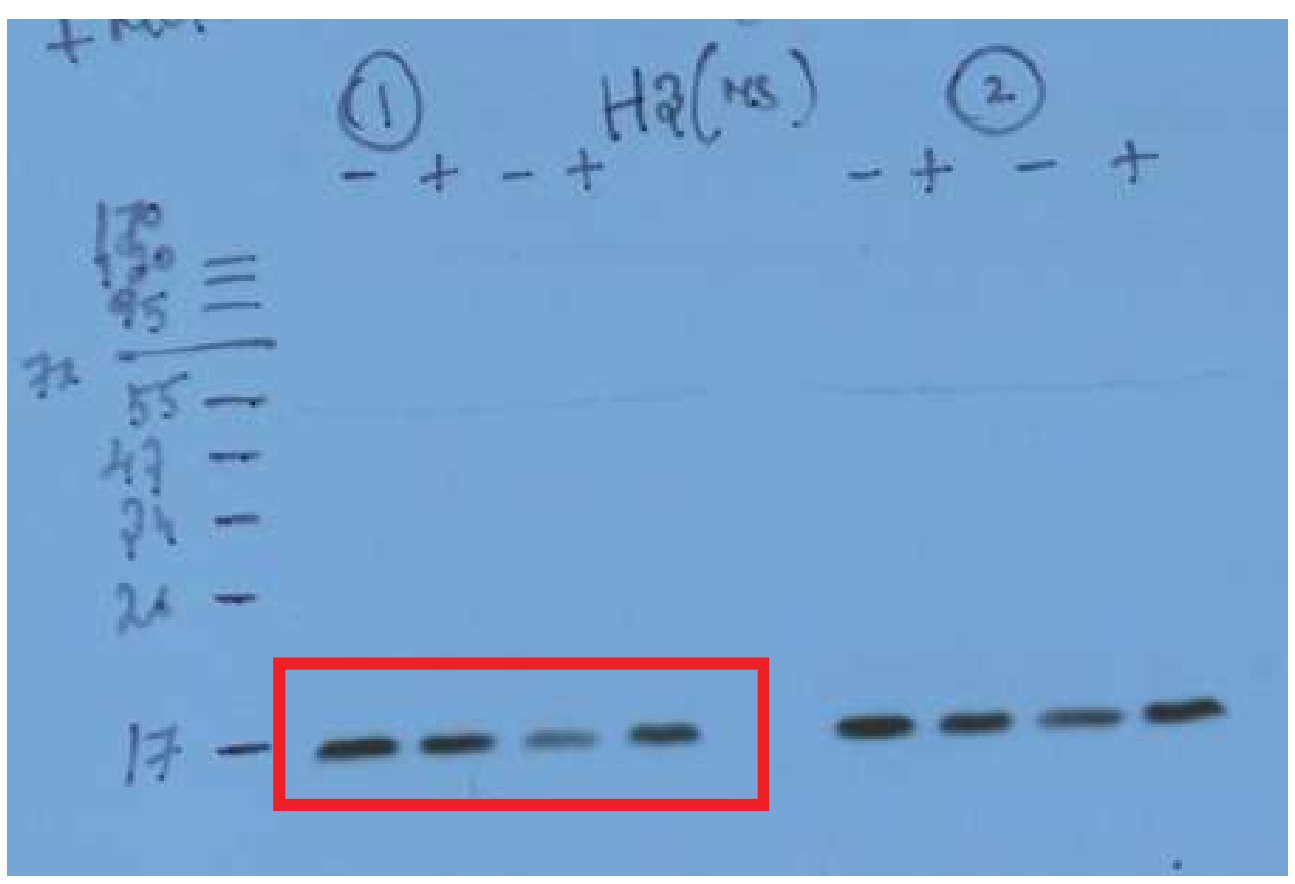

G6PDH for H3

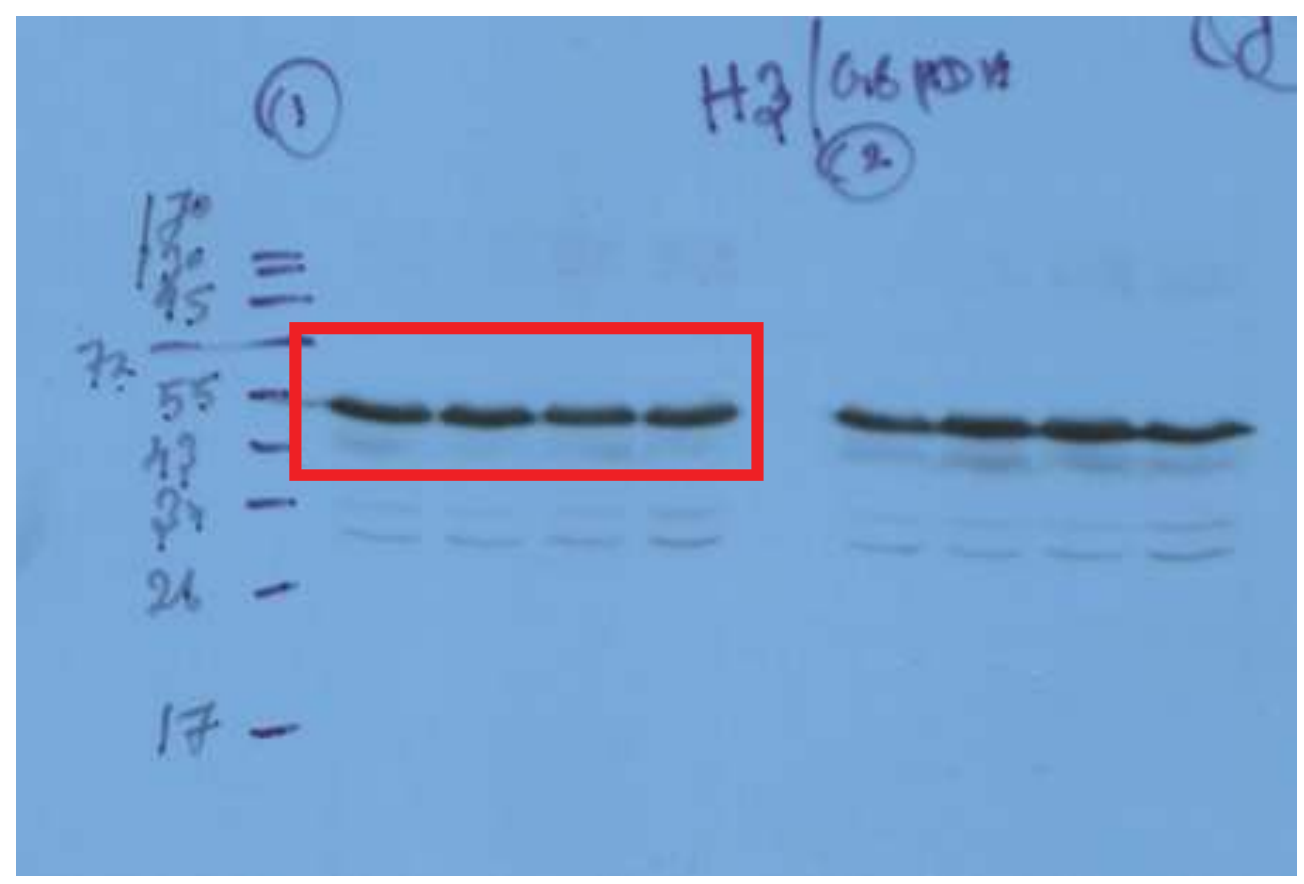

H2A

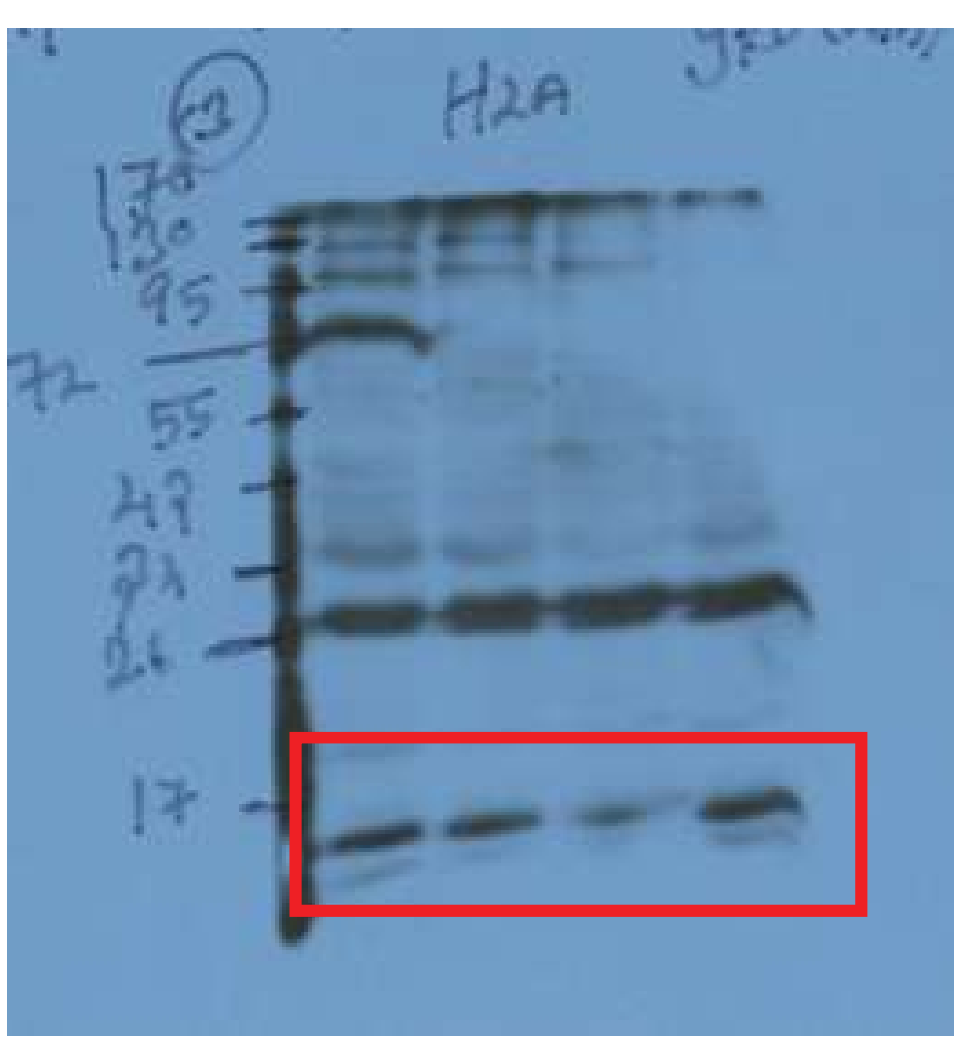

G6PDH for H2A

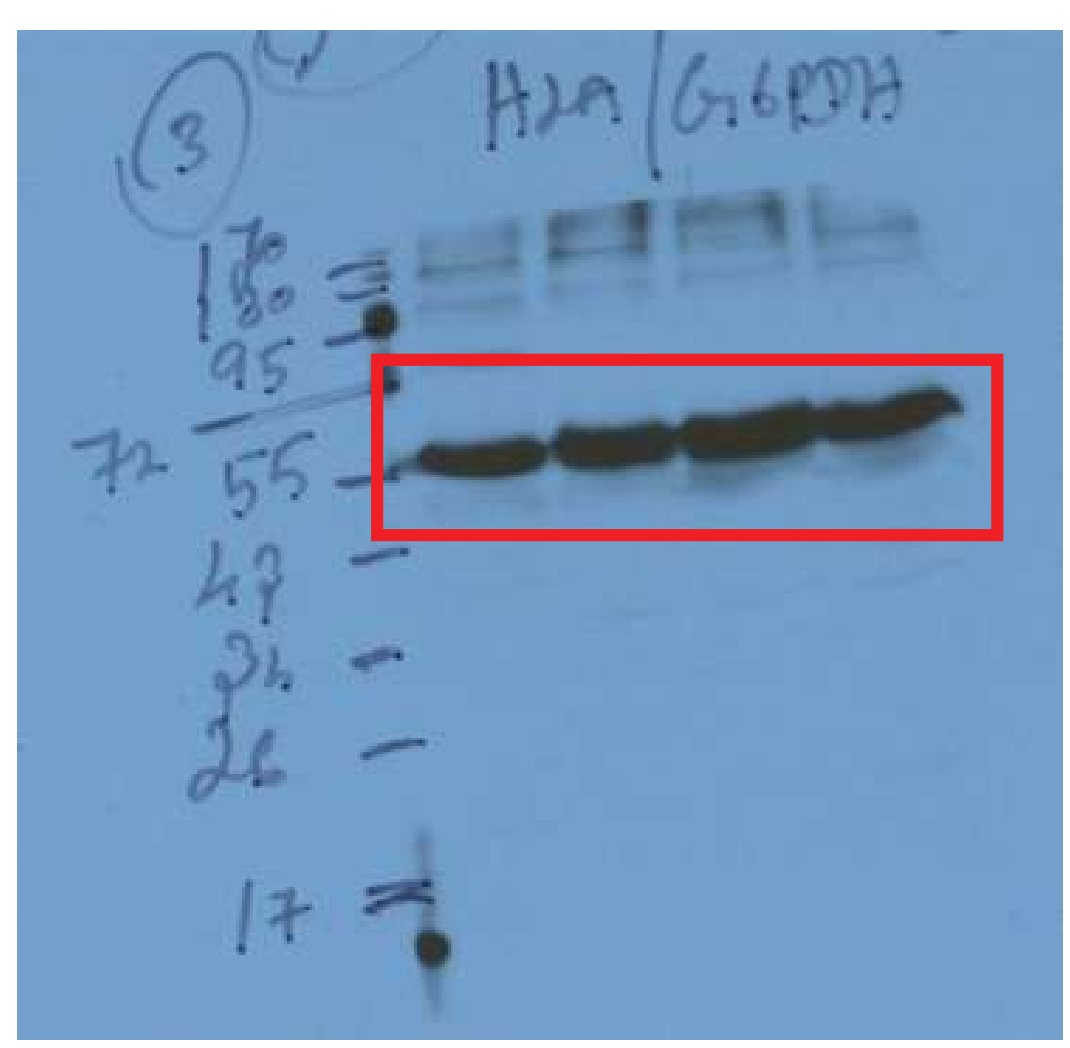

Figure 2G

H3

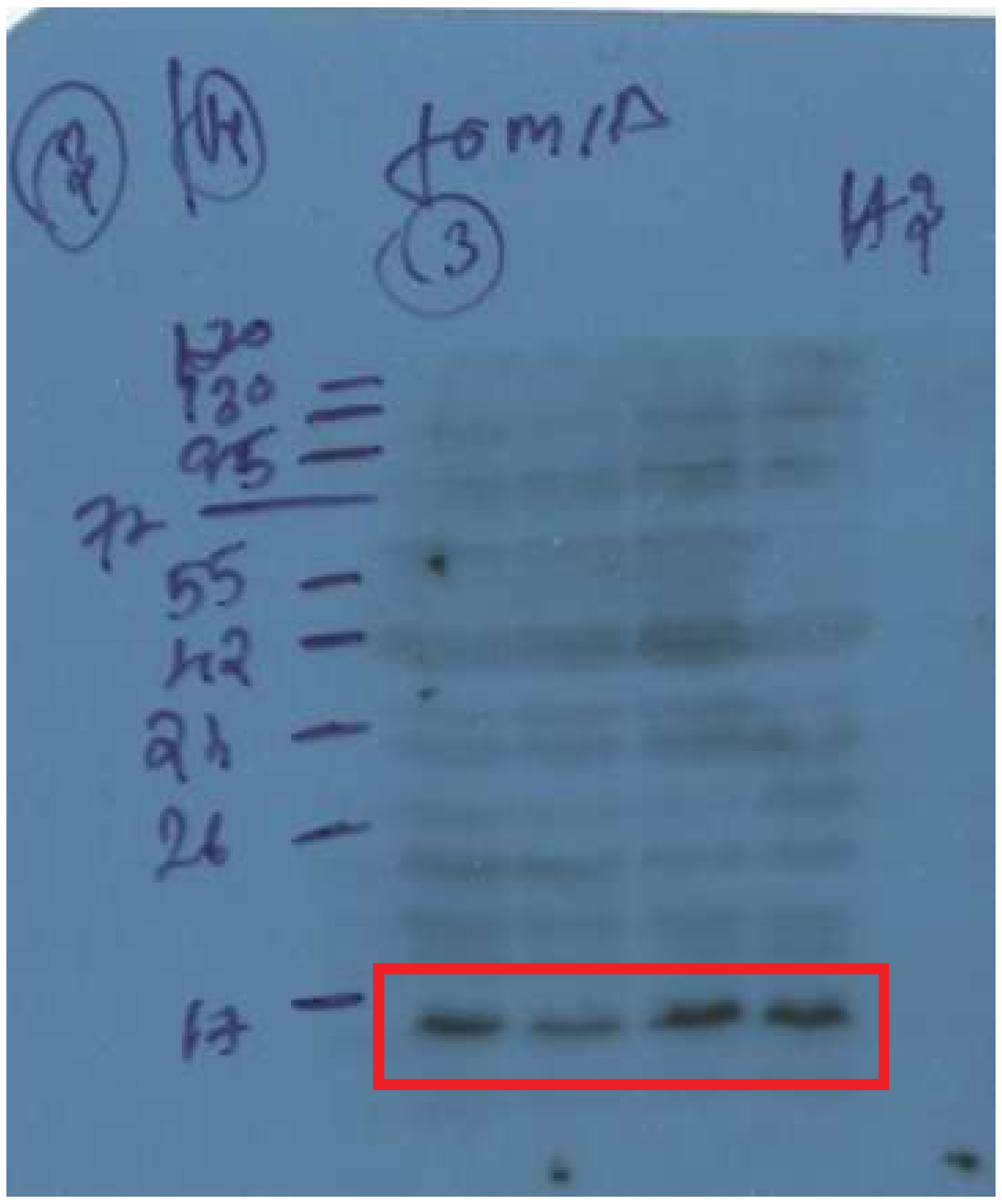

G6PDH for H3

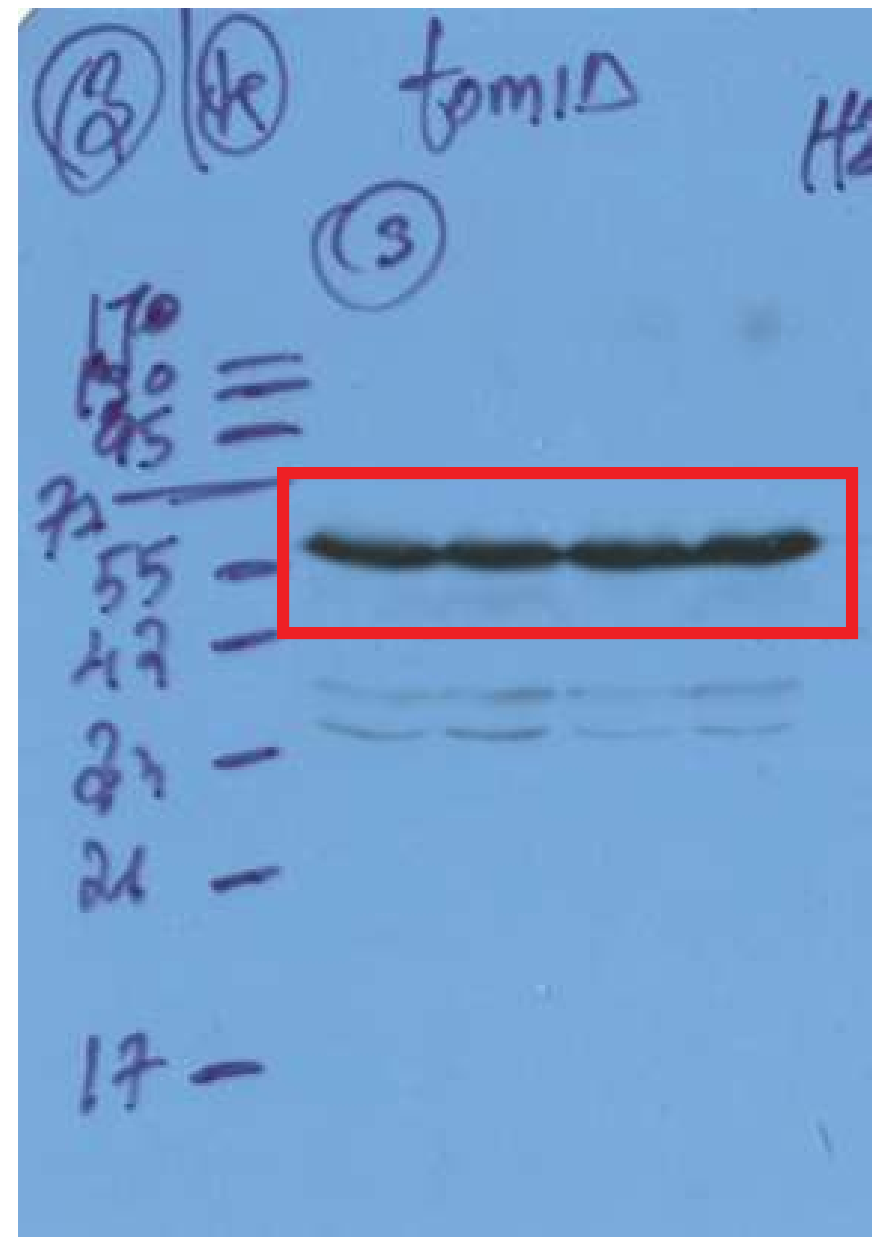

H2A

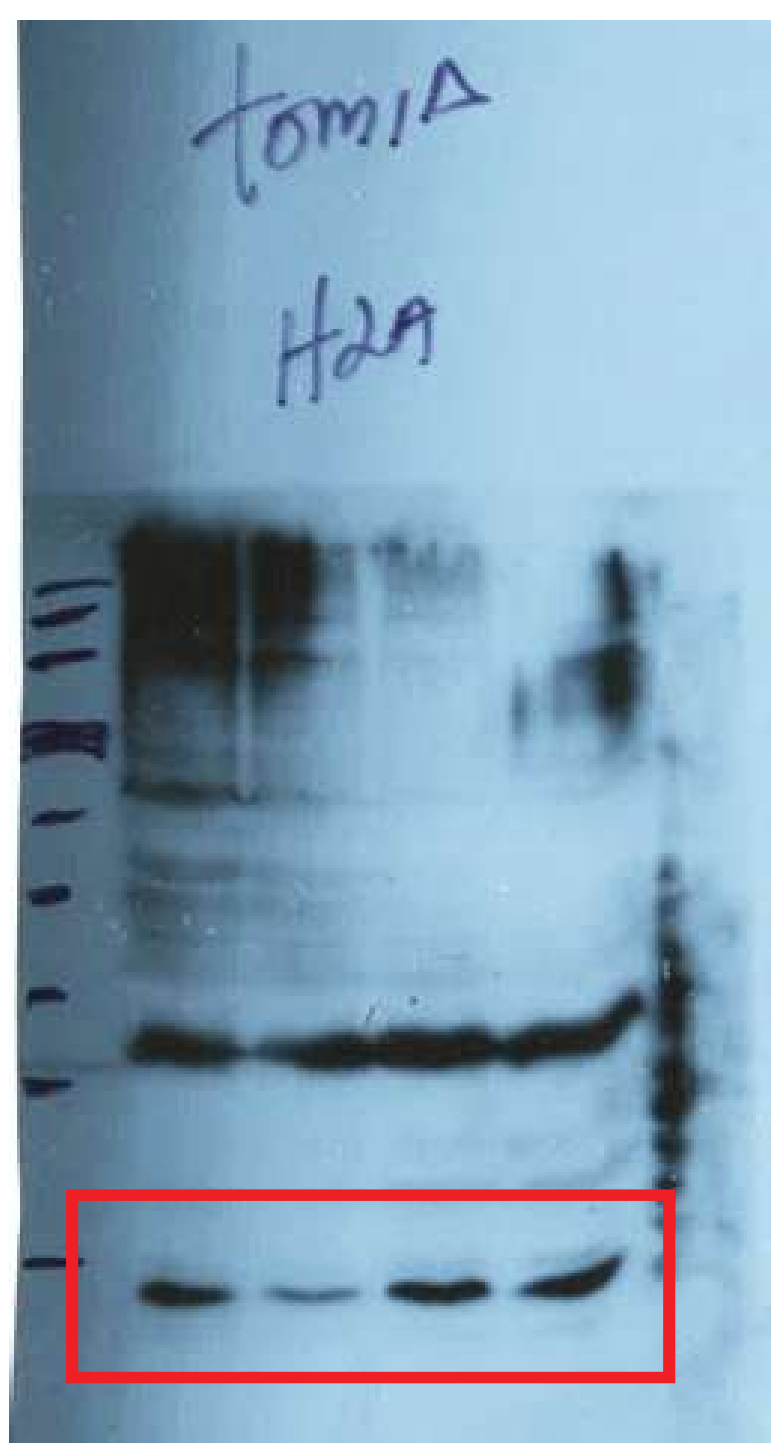

G6PDH for H2A

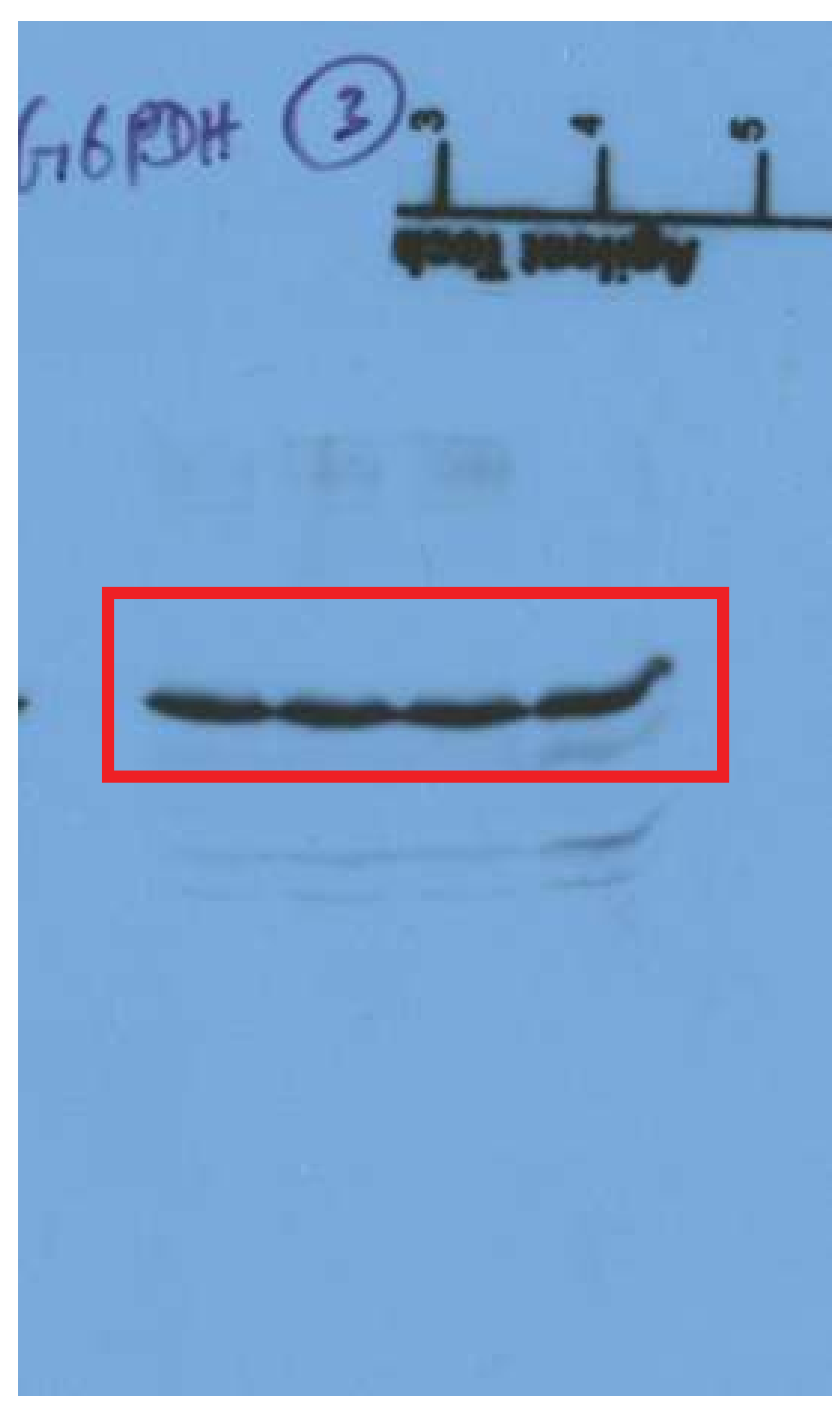

Figure 3C

H3

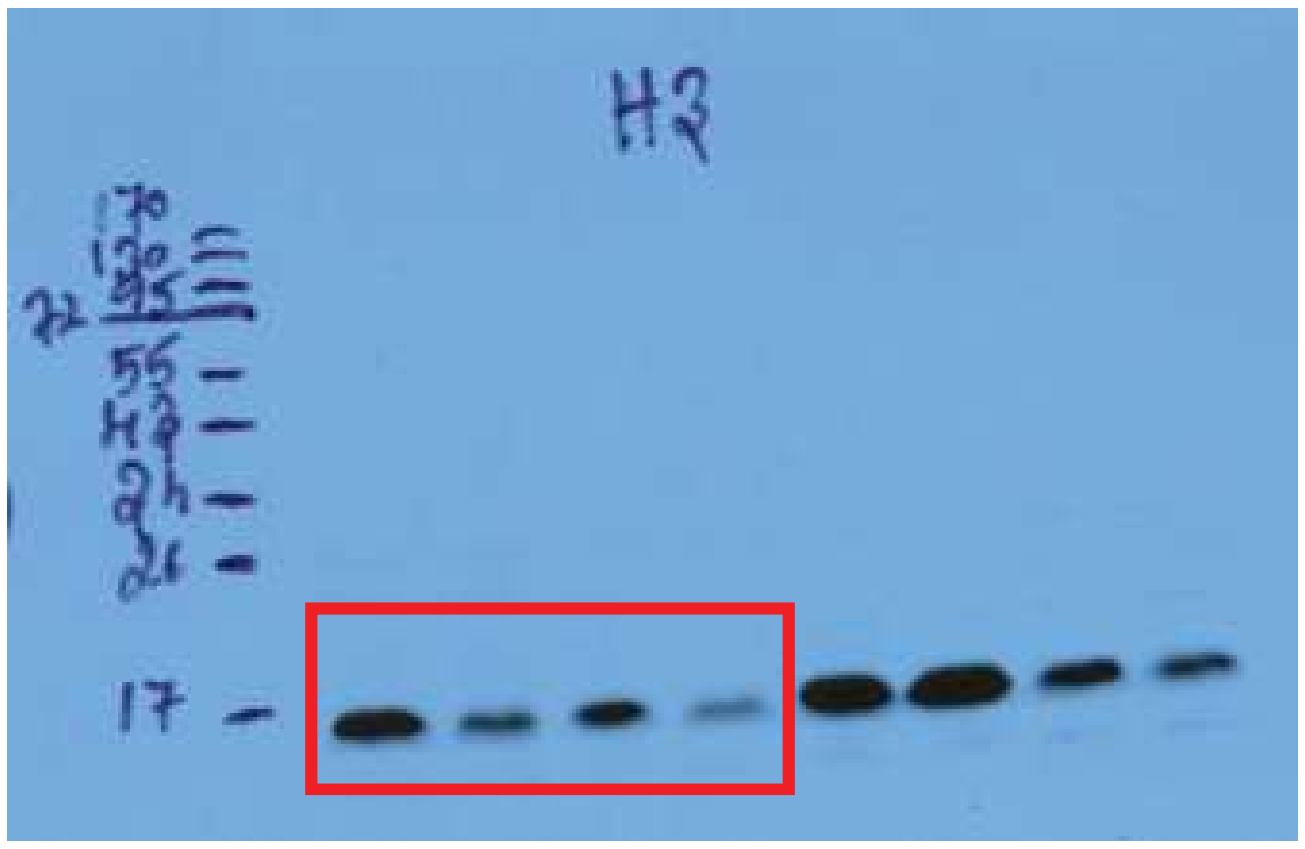

G6PDH for H3

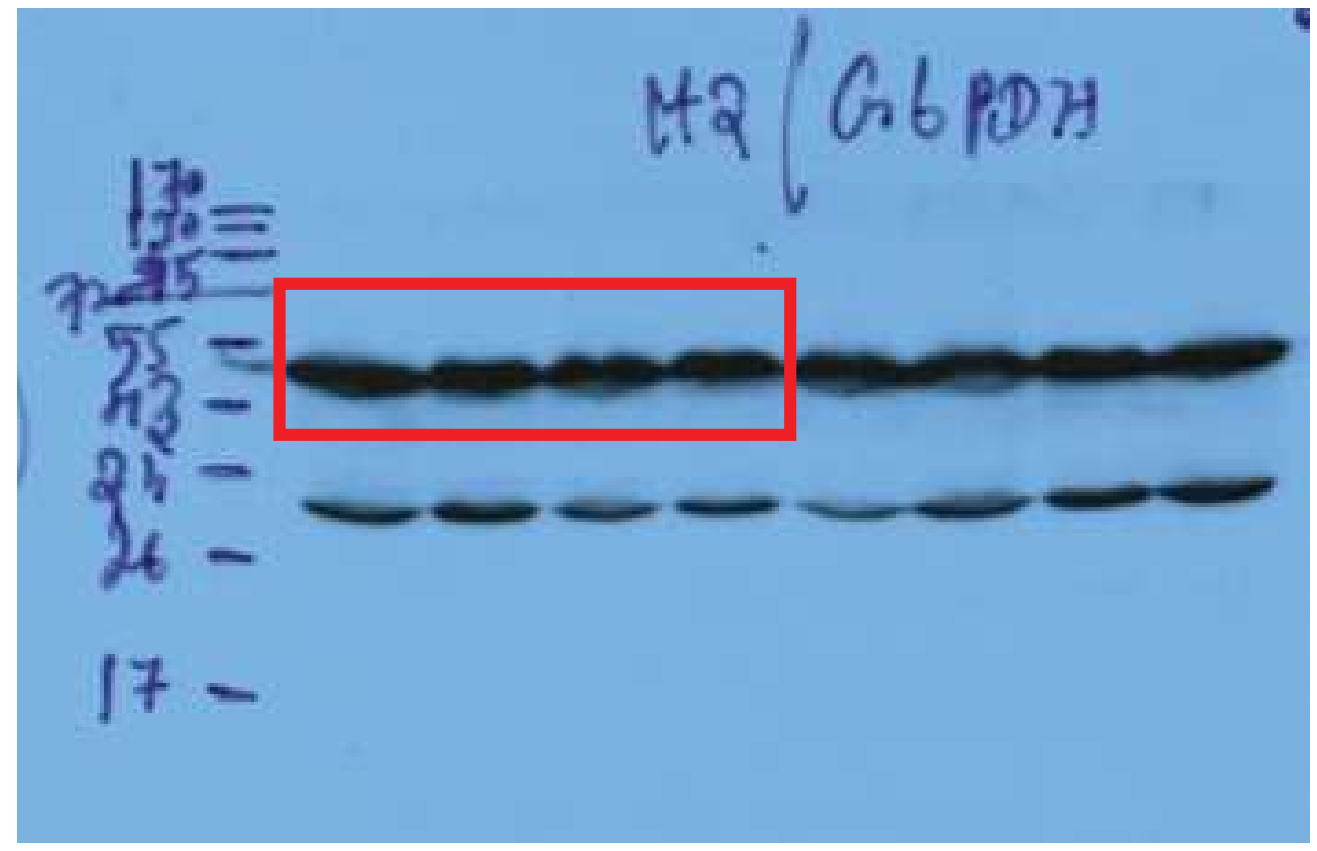

H3K36me3

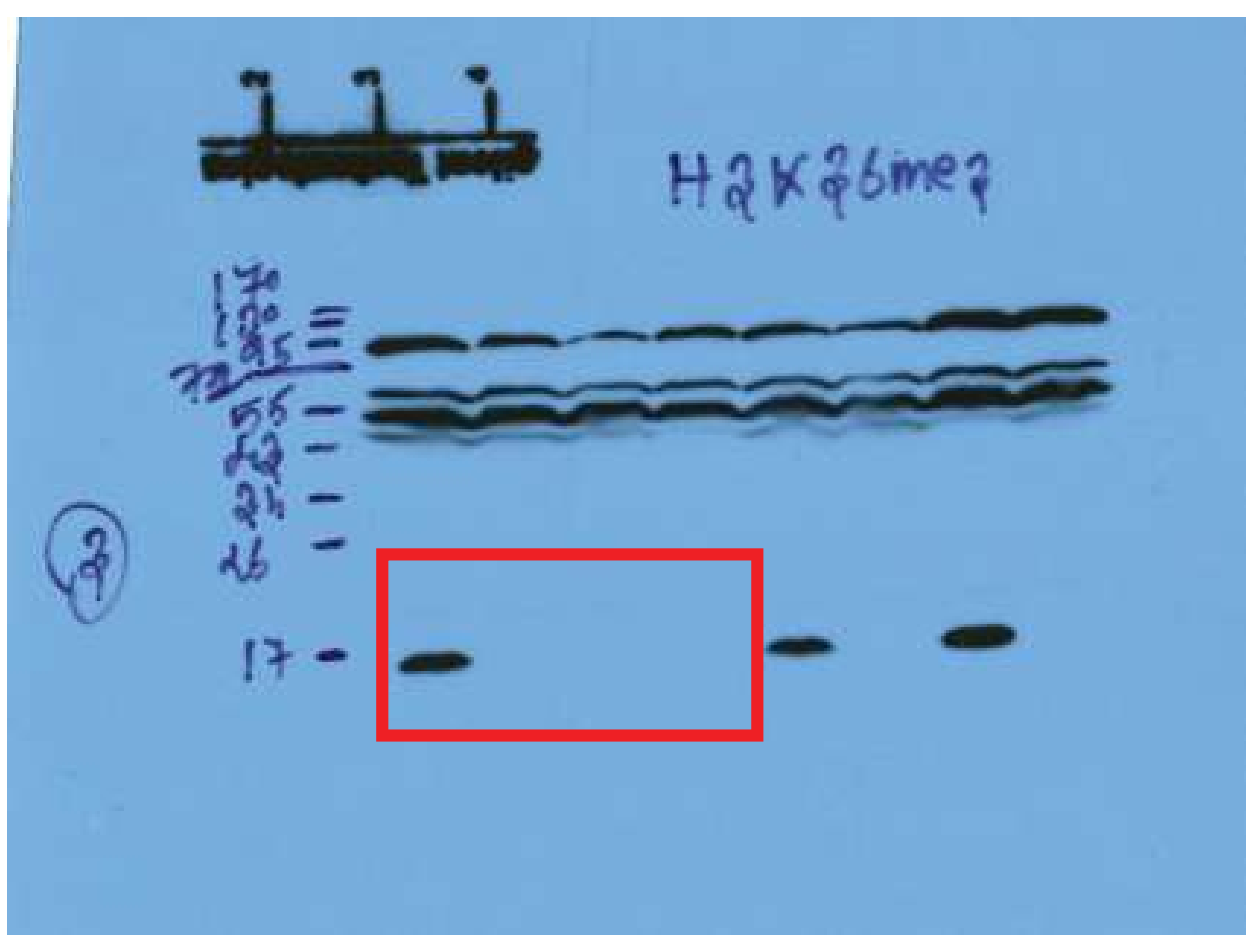

G6PDH for H3K36me3

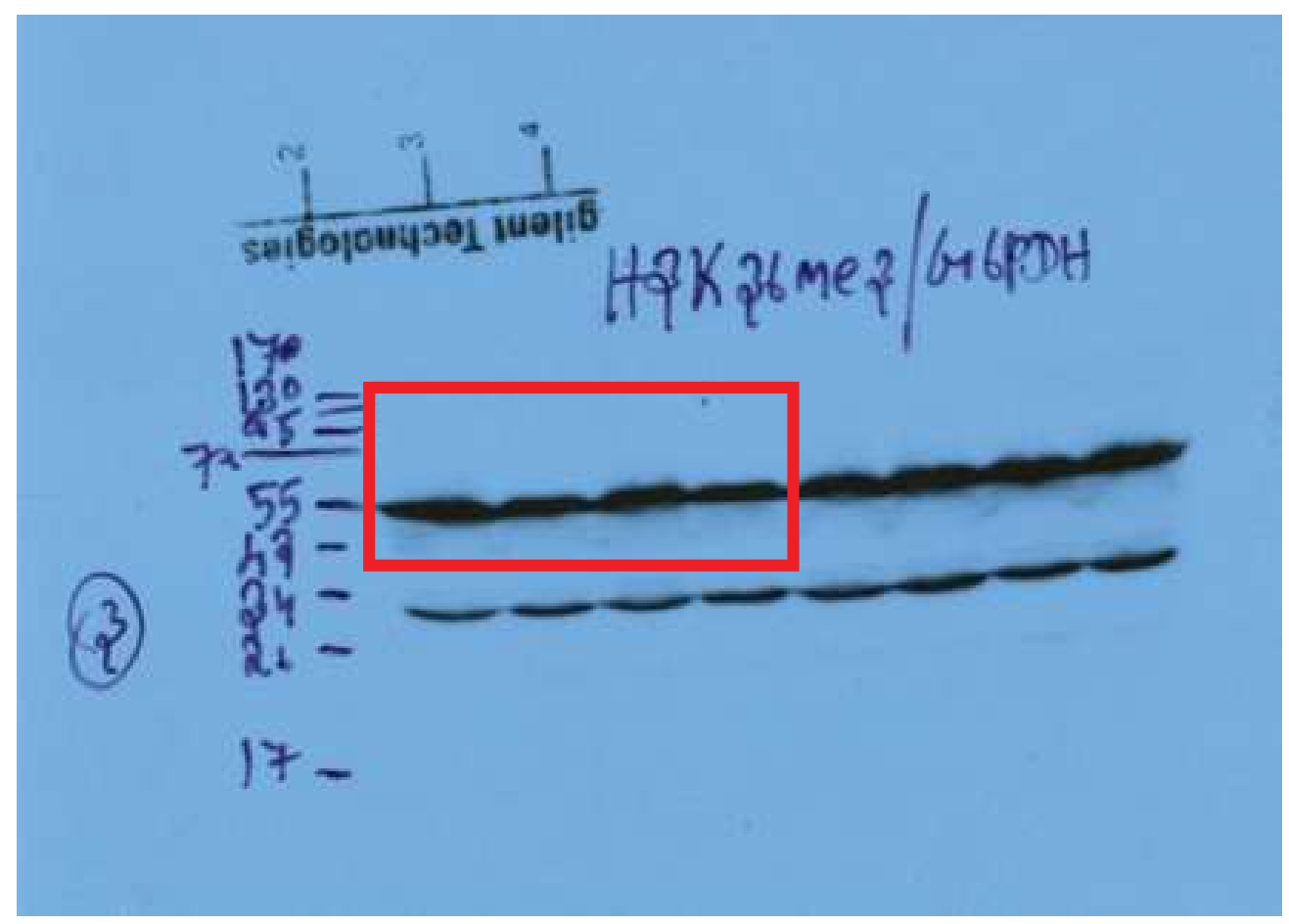

Figure 3E

Set2-HA

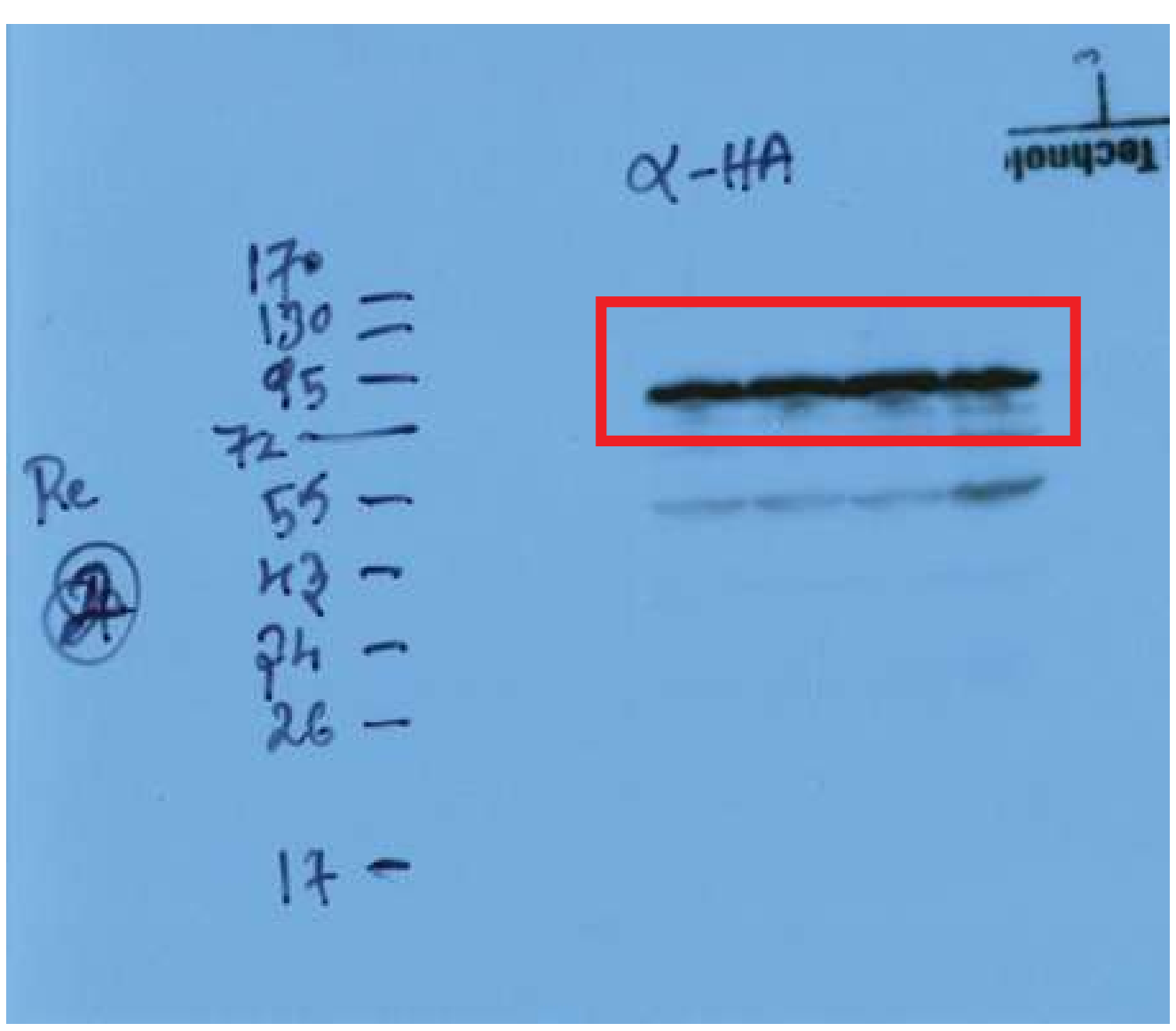

G6PDH for Set2-HA

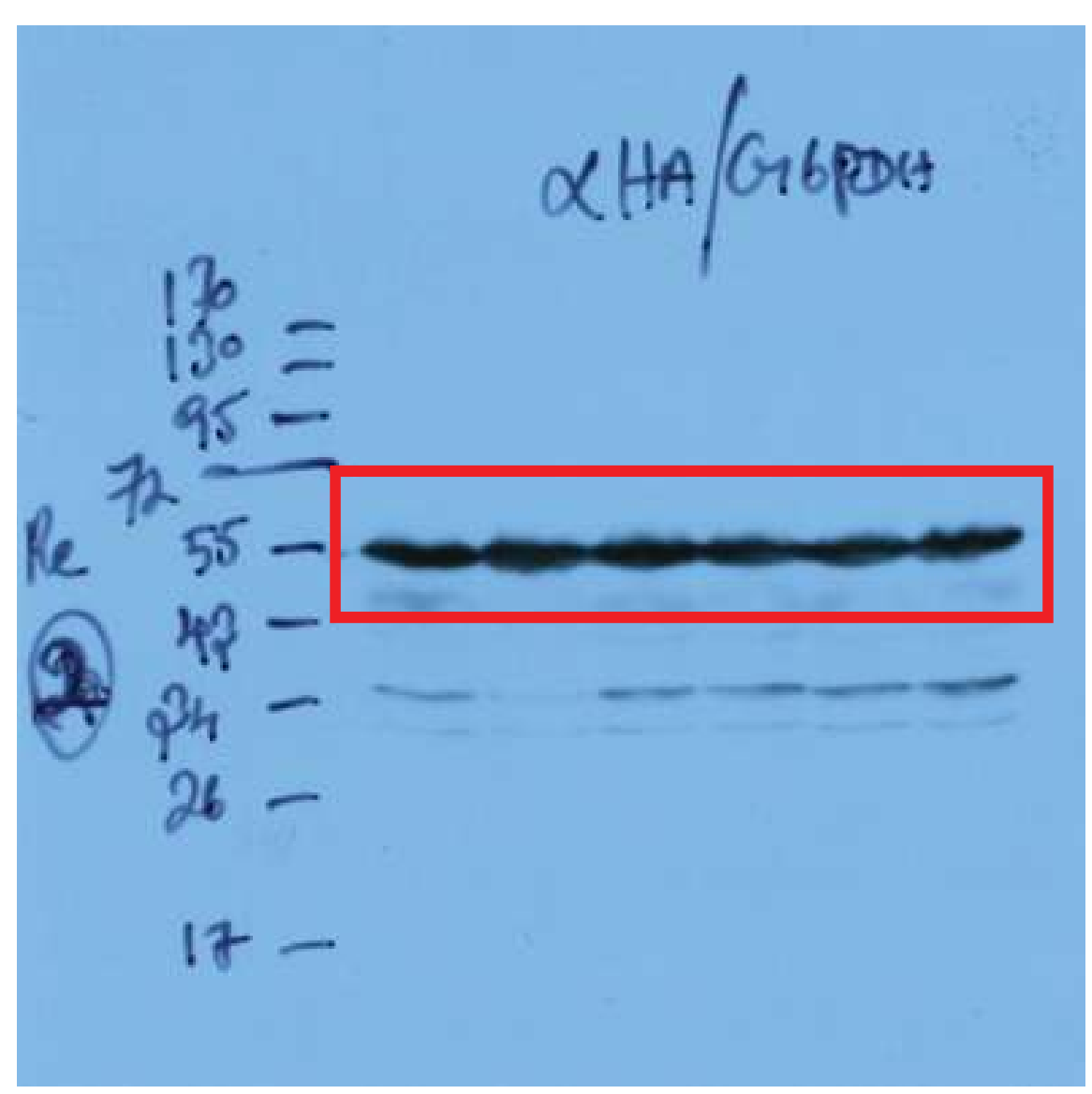

Figure 4E

H3

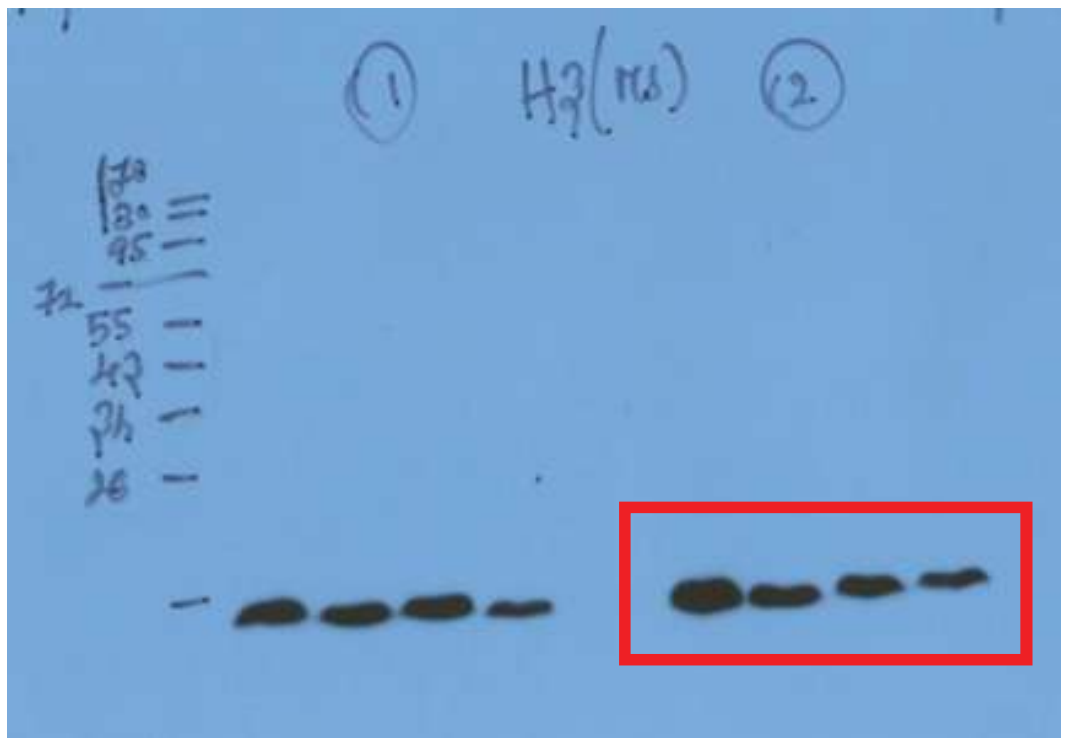

G6PDH for H3

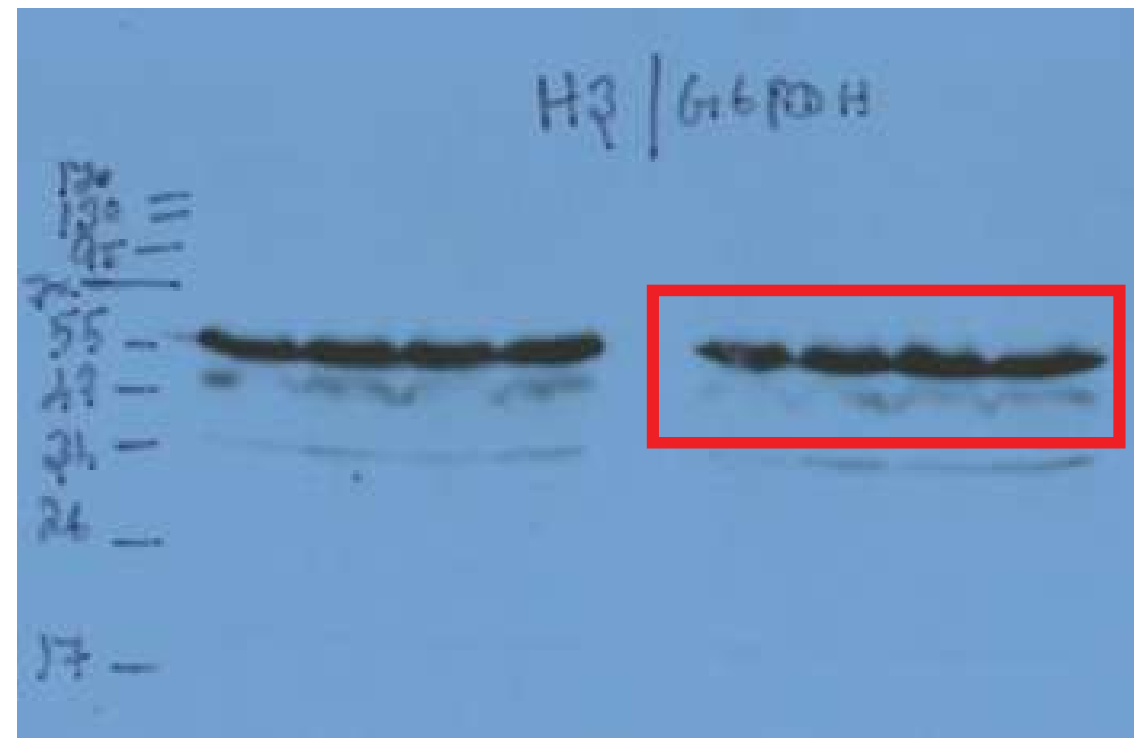

H4

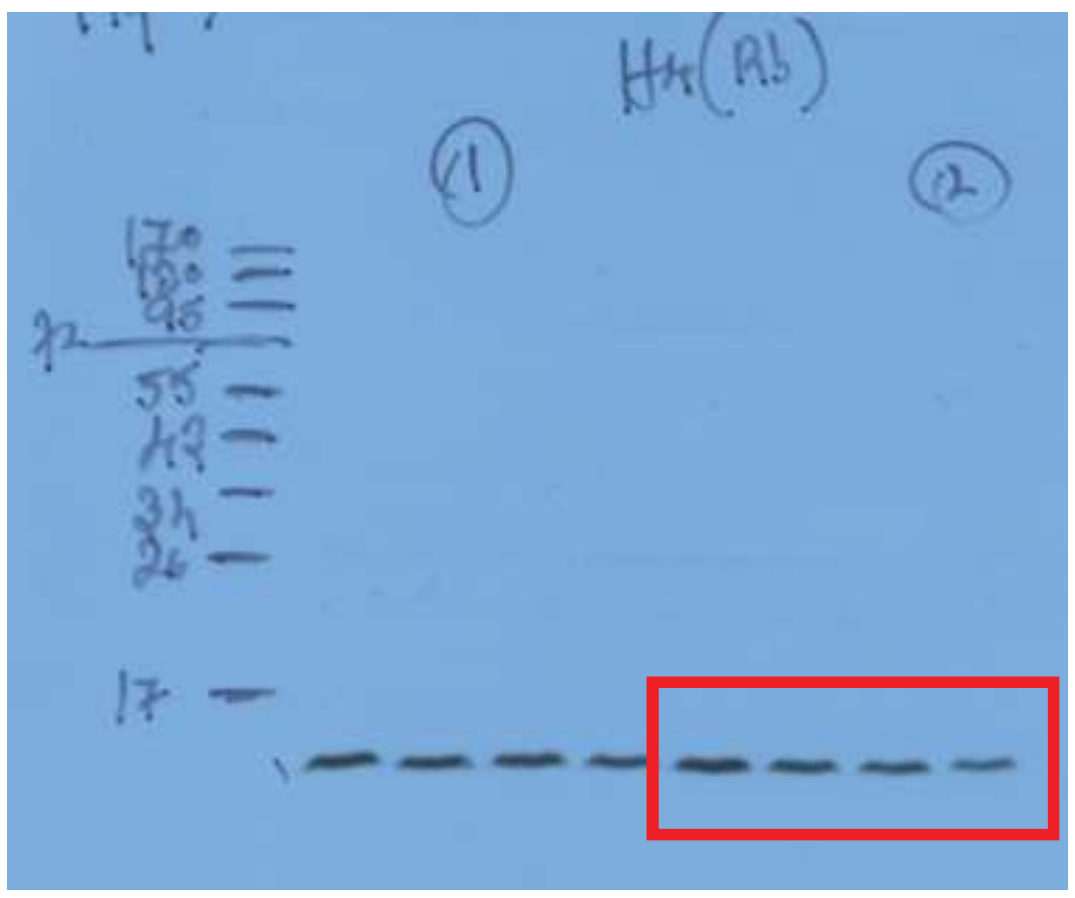

G6PDH for H4

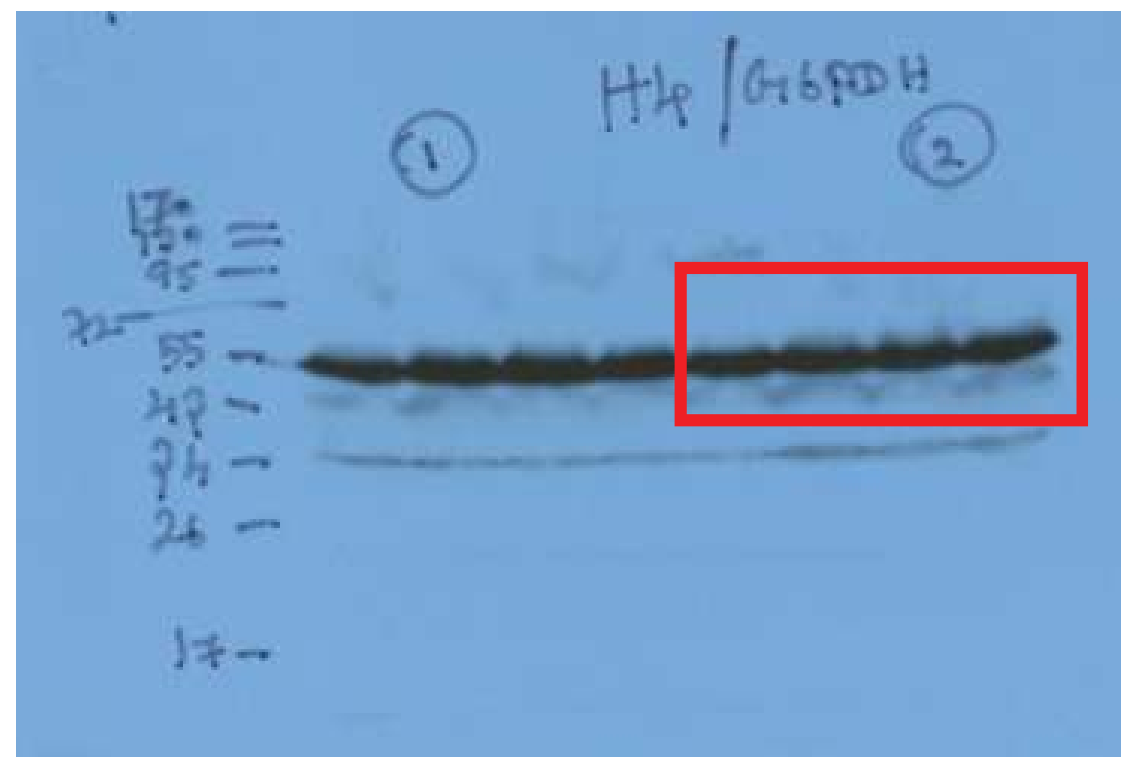

Figure 4H

H3

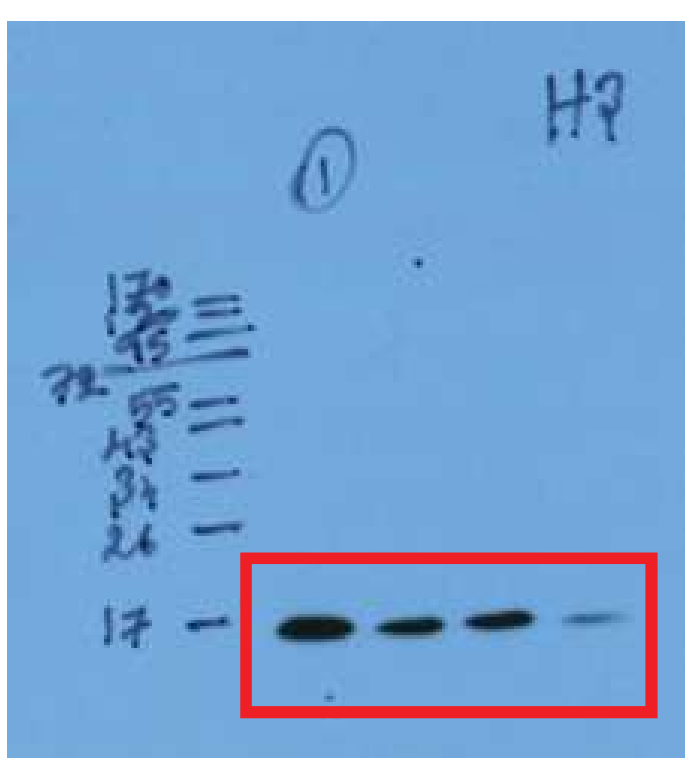

G6PDH for H3

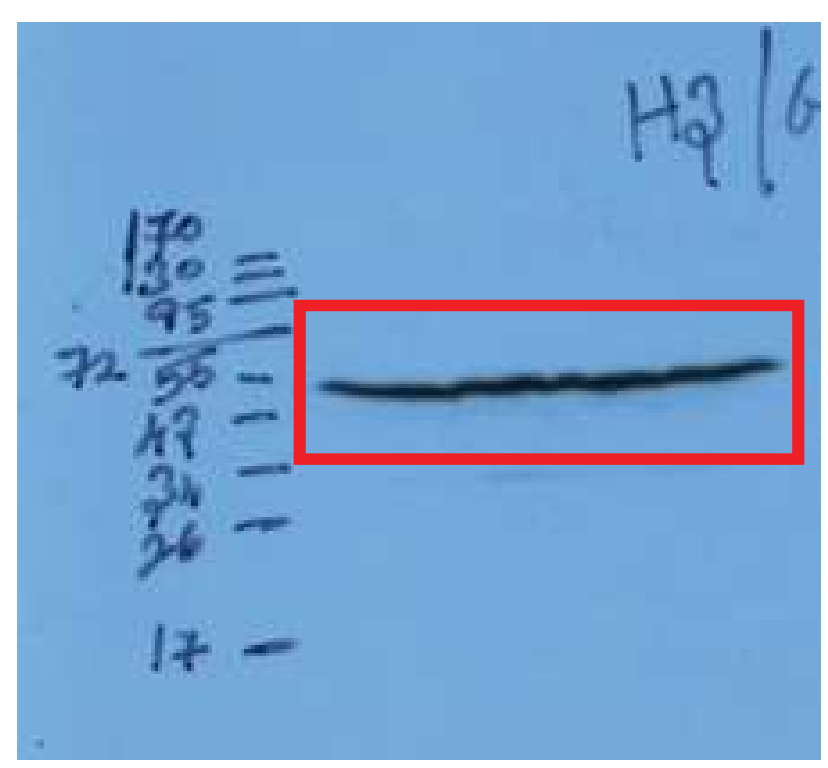

H4

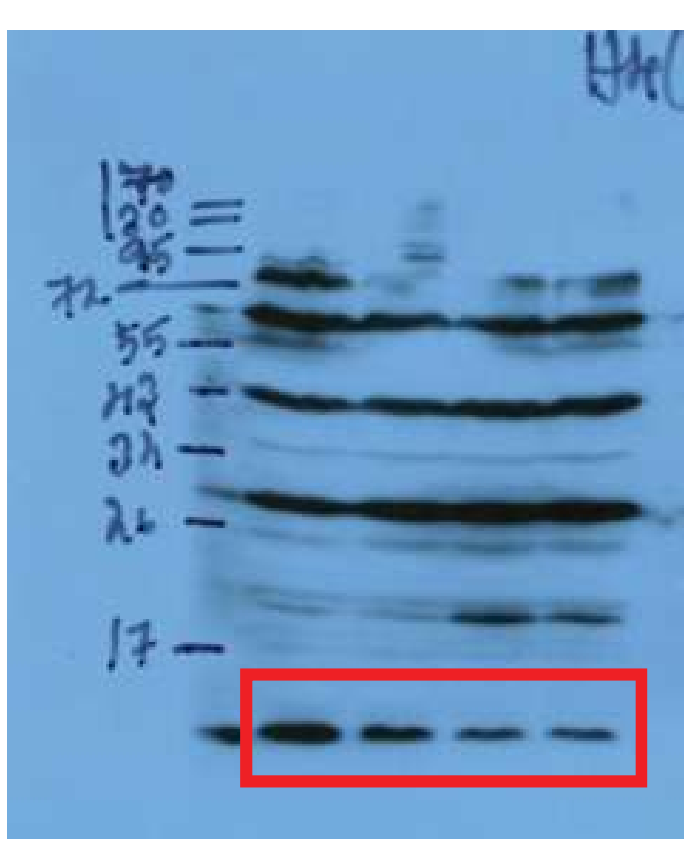

G6PDH for H4

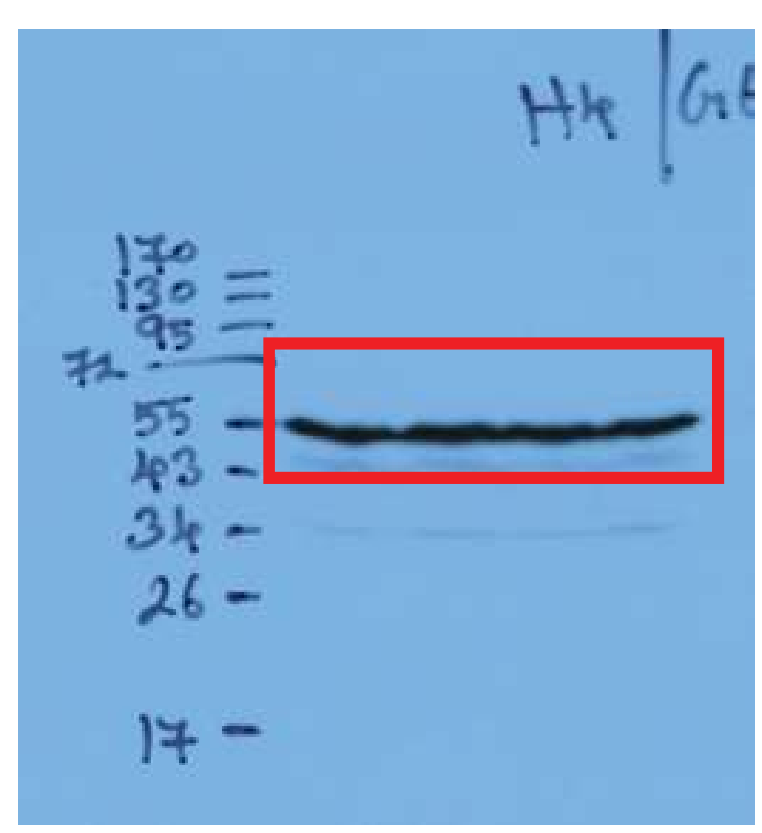

Figure 5A

S6ph S6 - Total

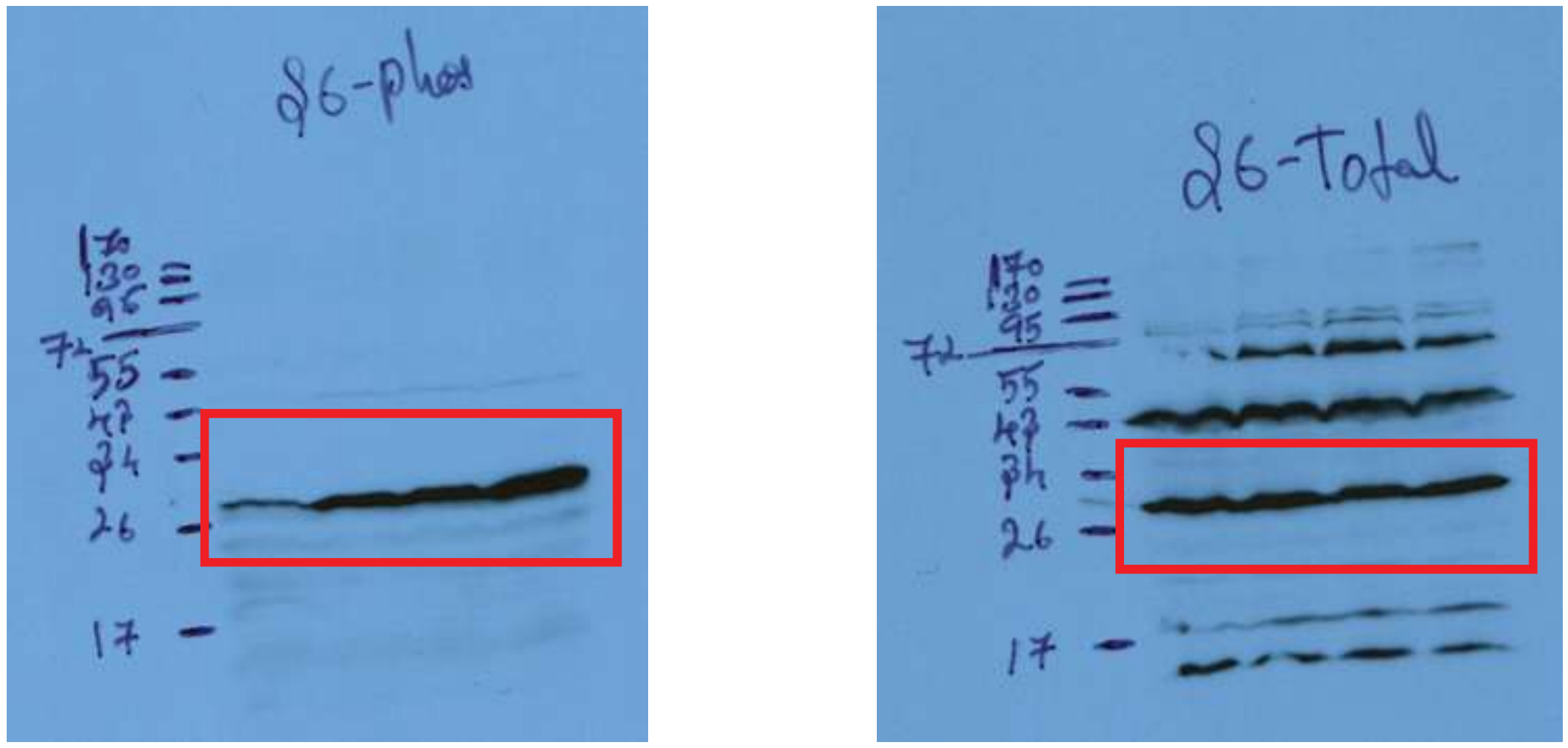

Figure 5C

S6ph S6 - Total

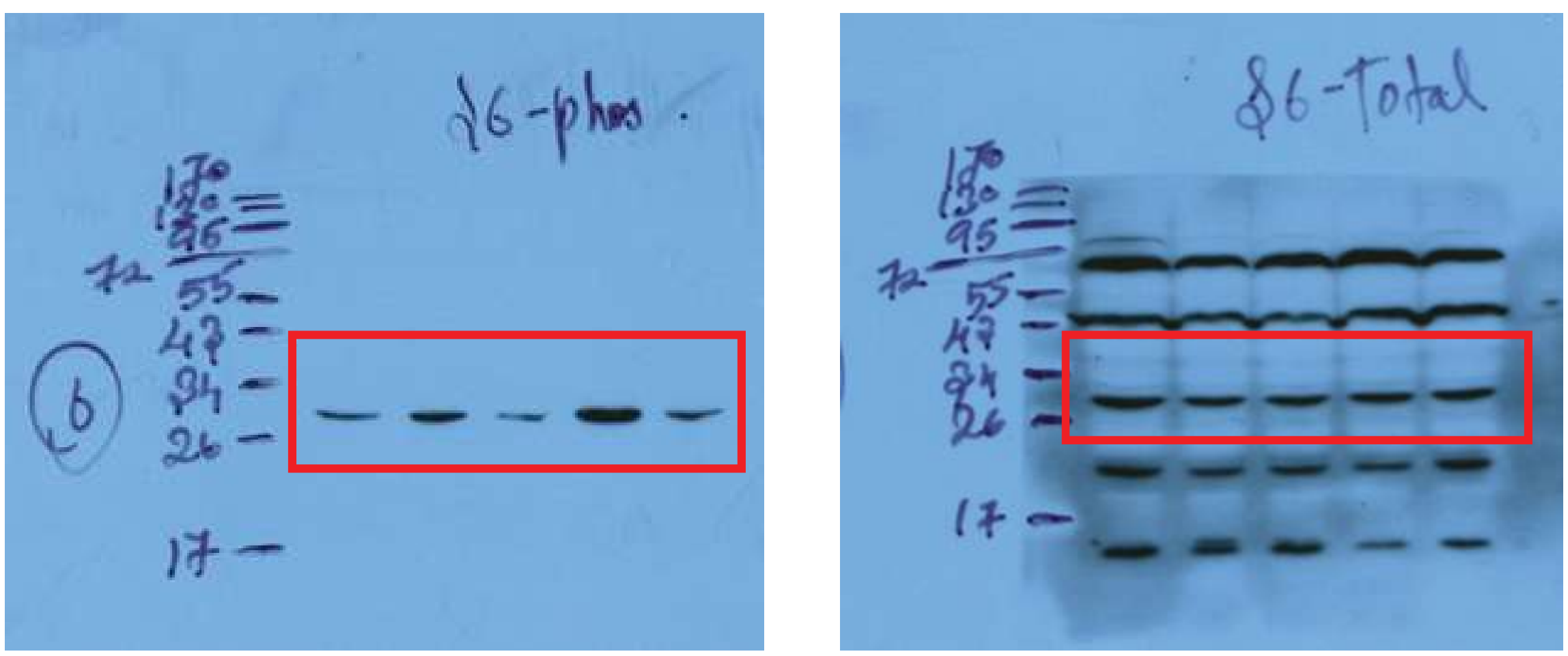

Figure 5E

S6ph S6 - Total

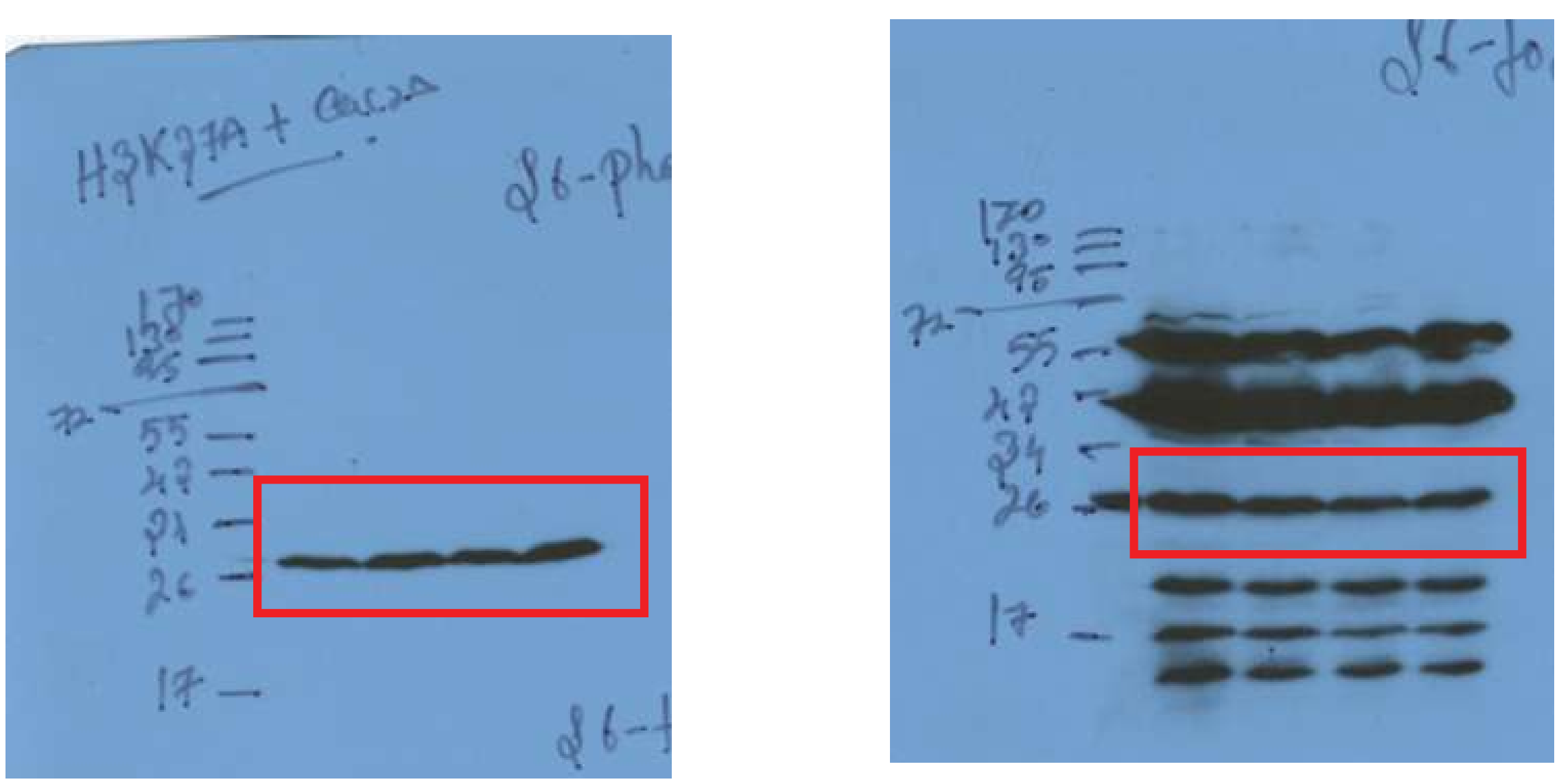

Figure 5G

S6ph

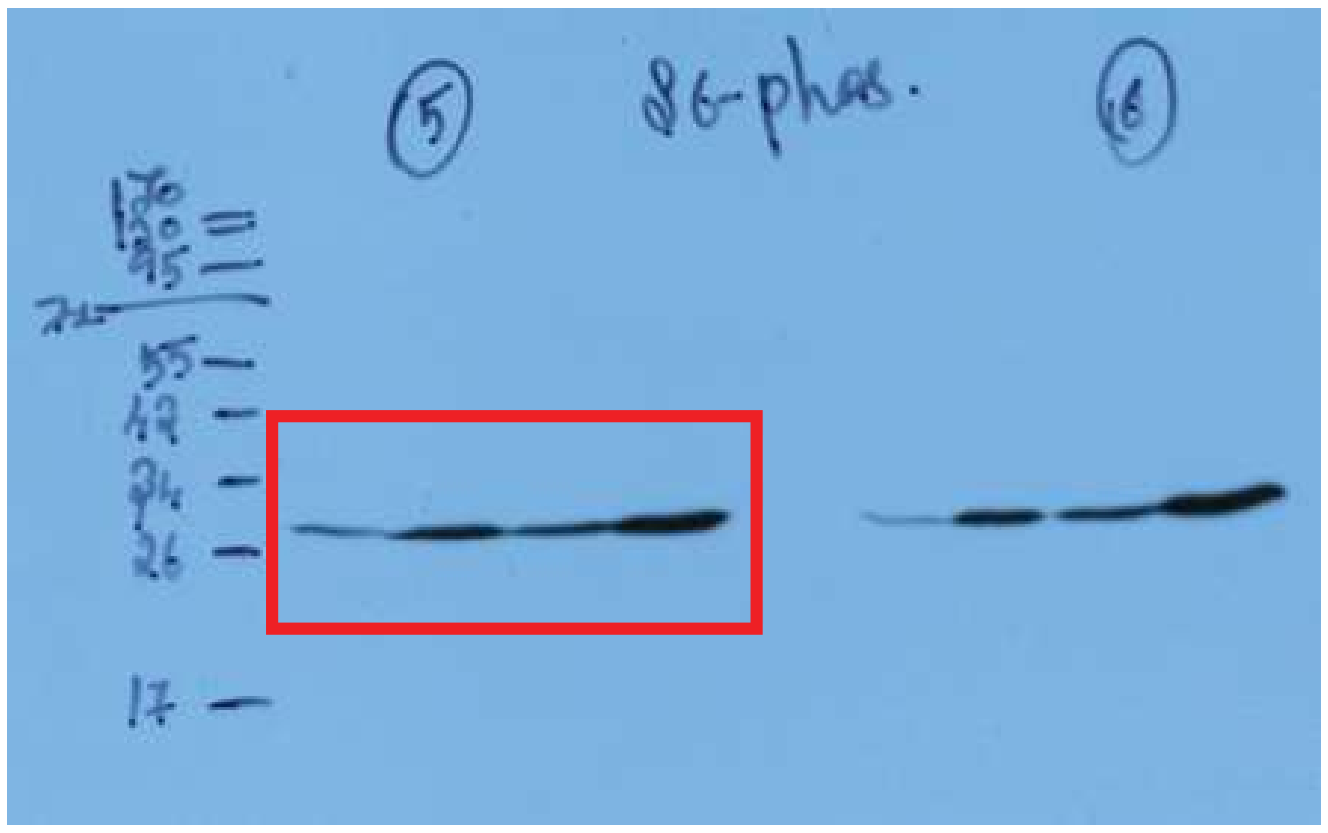

S6 - Total

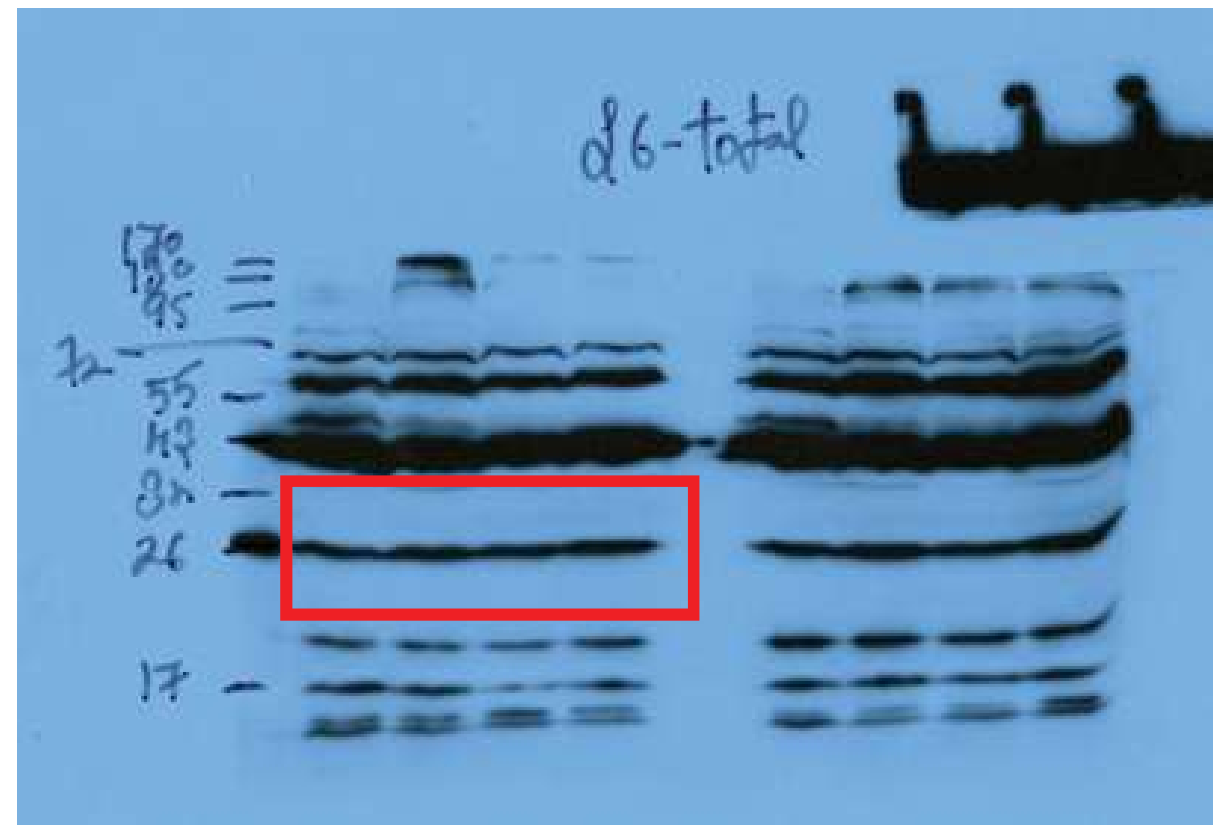

Figure 5I

S6ph

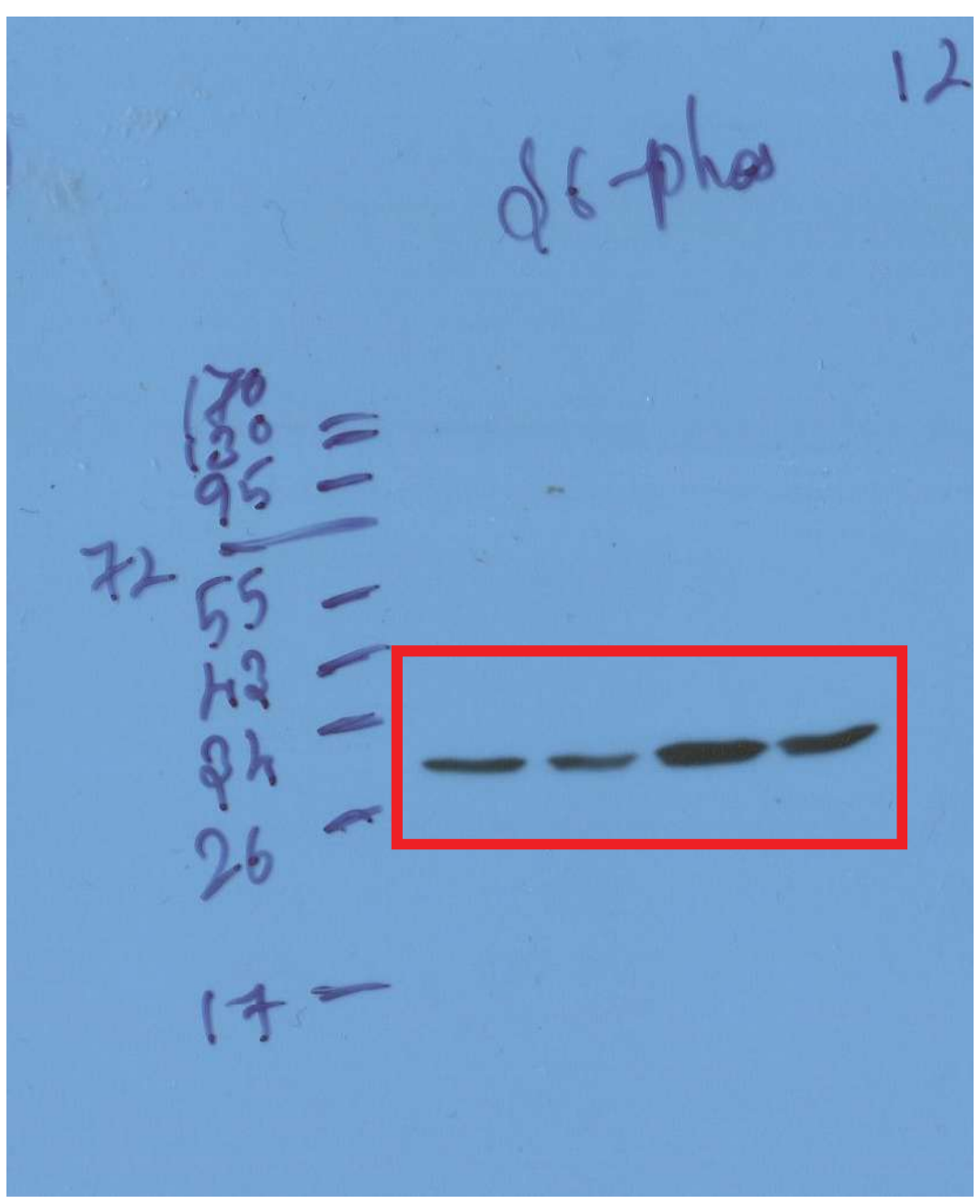

S6 - Total

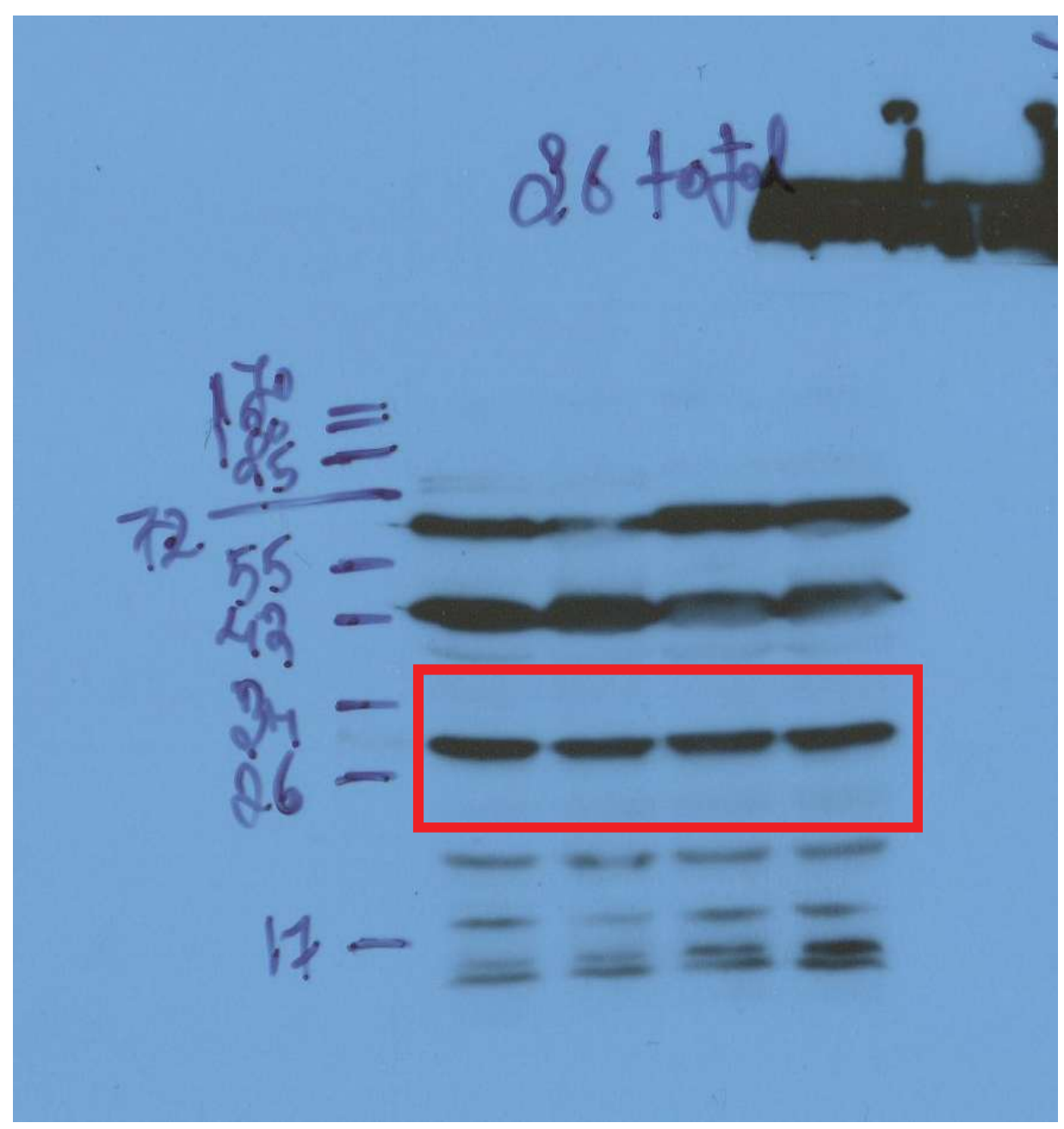

Figure 6A

S6ph

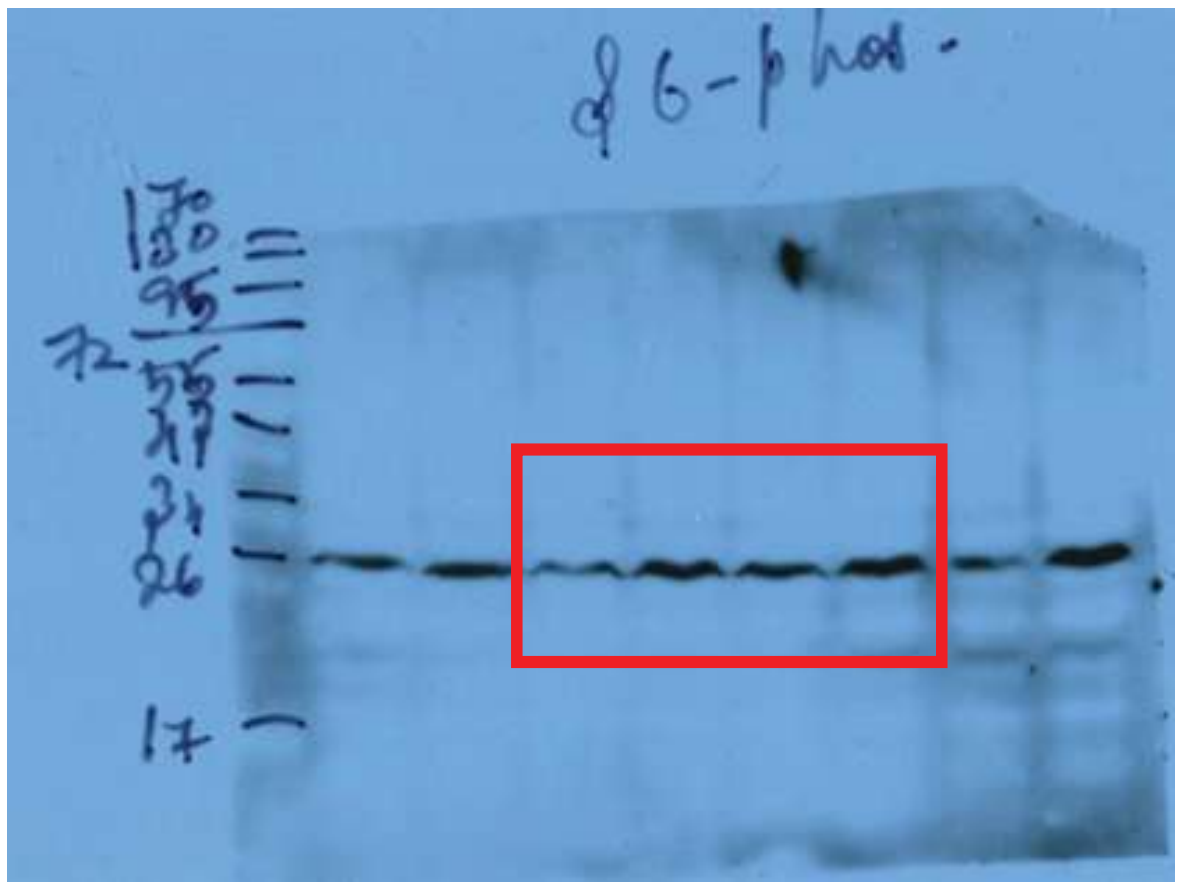

S6 - Total

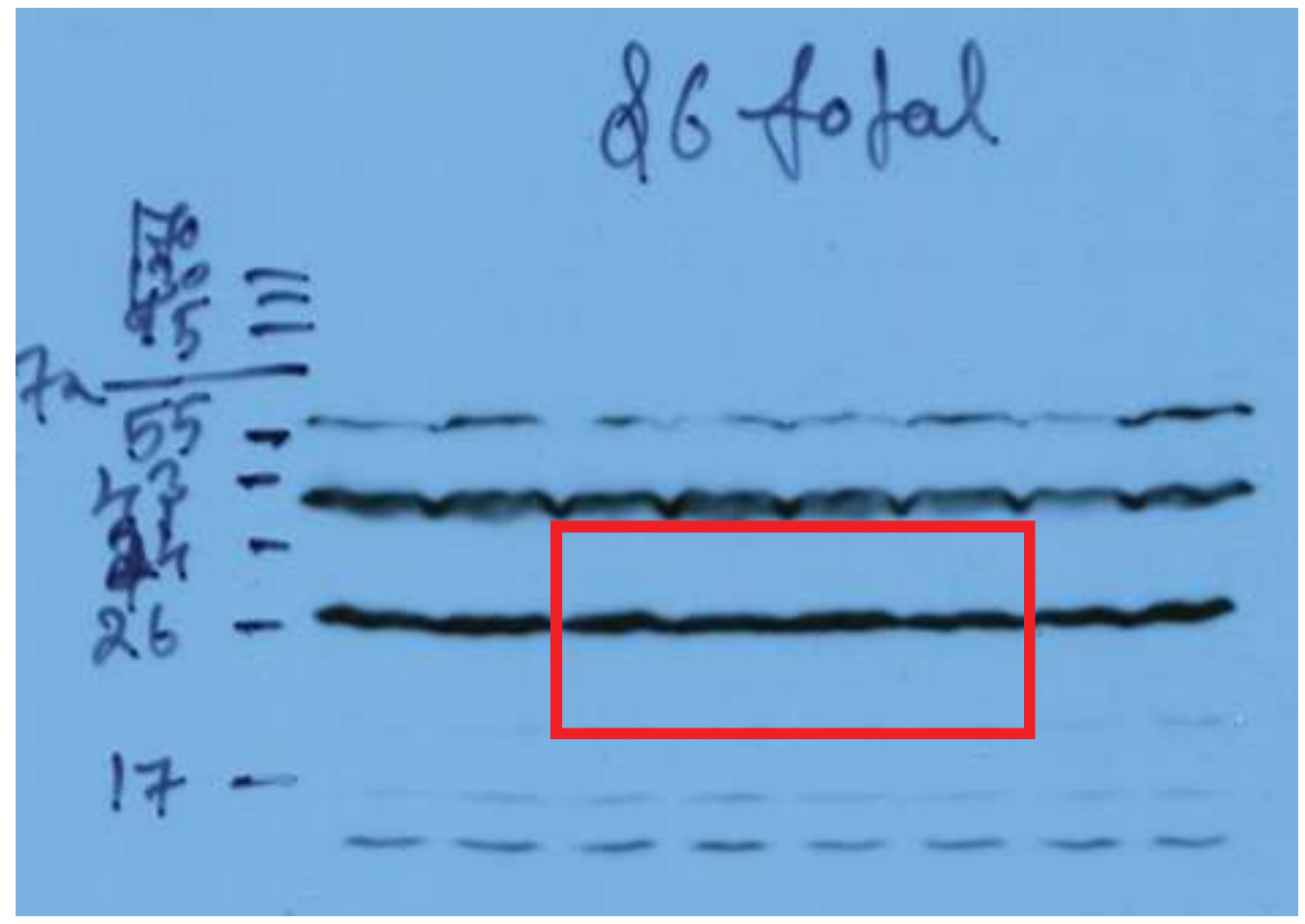

Tco89-Flag

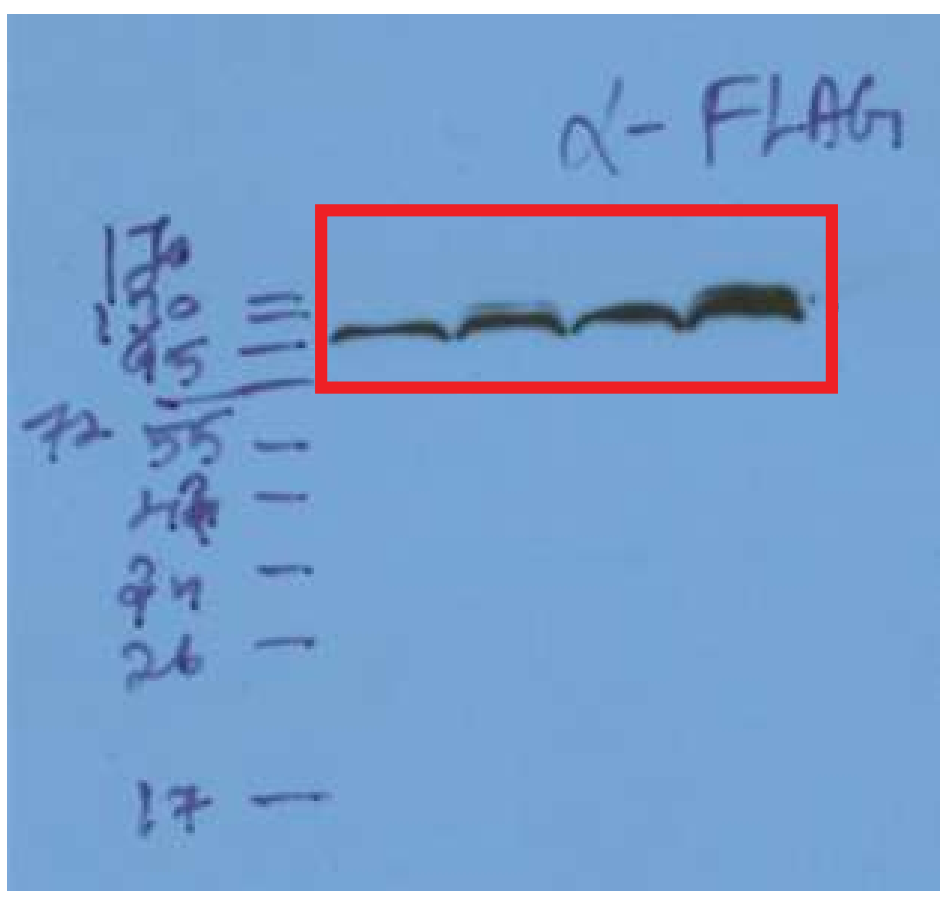

Figure 6D

S6ph

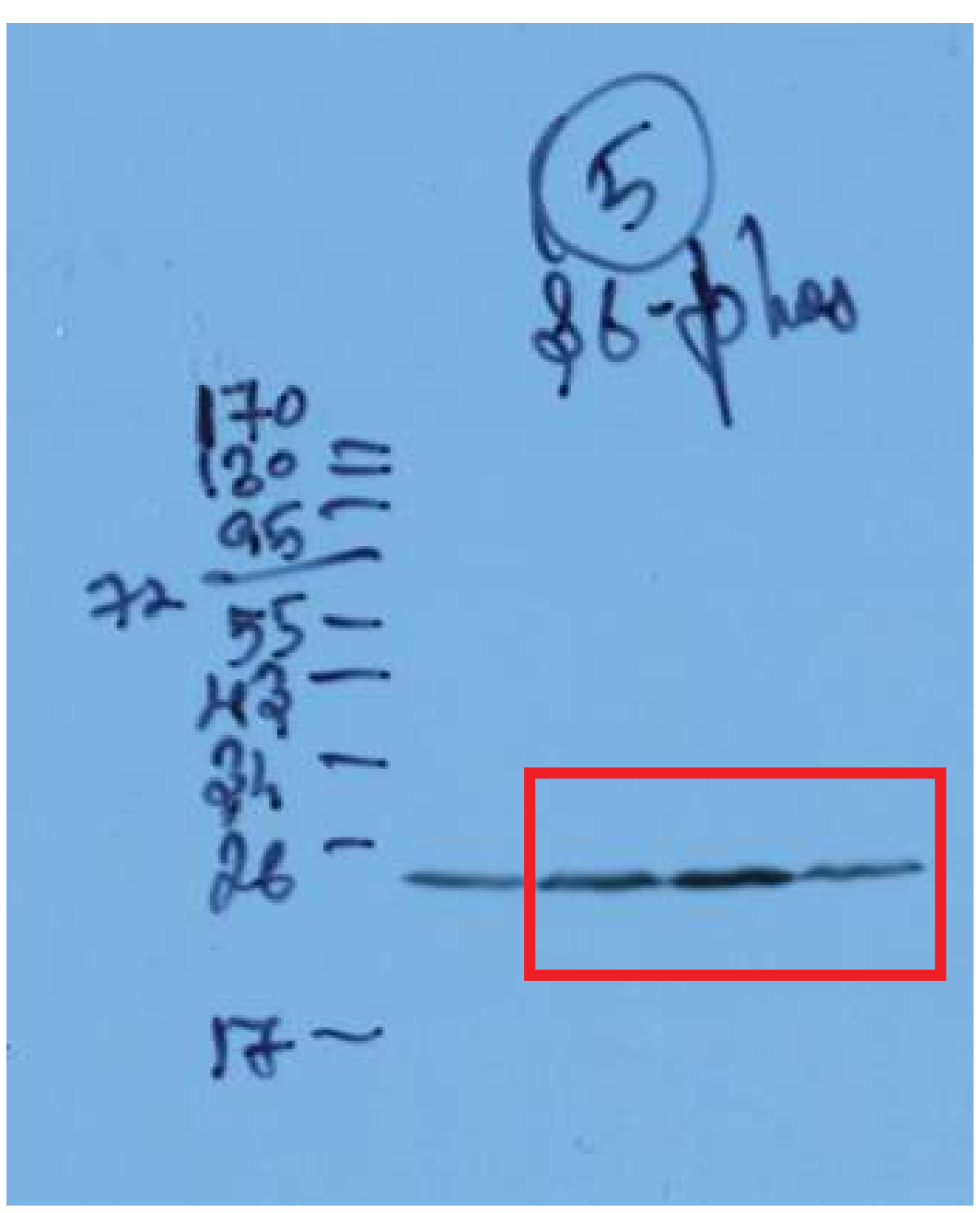

S6 - Total

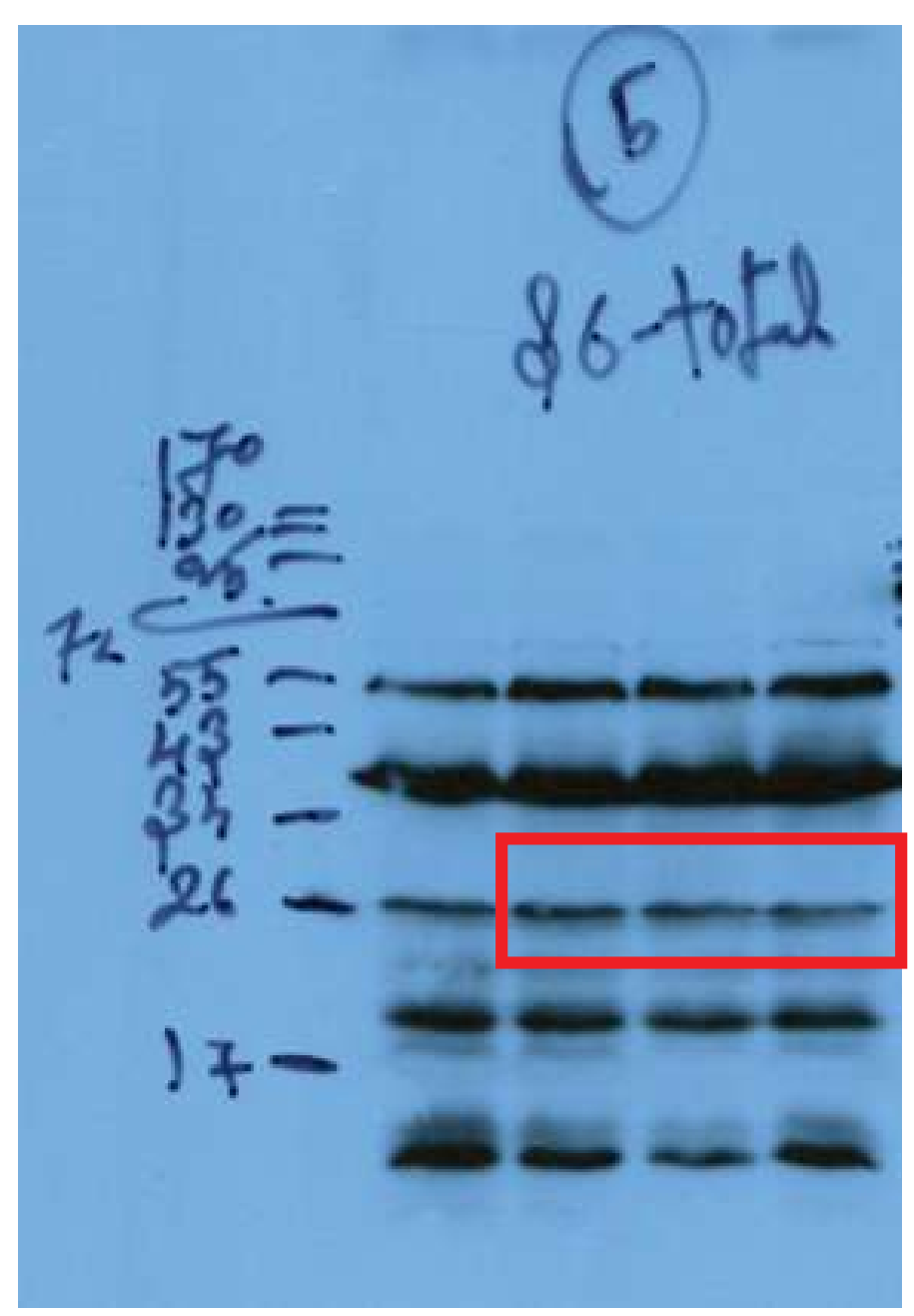

Figure 7D

COX4-HA - Short

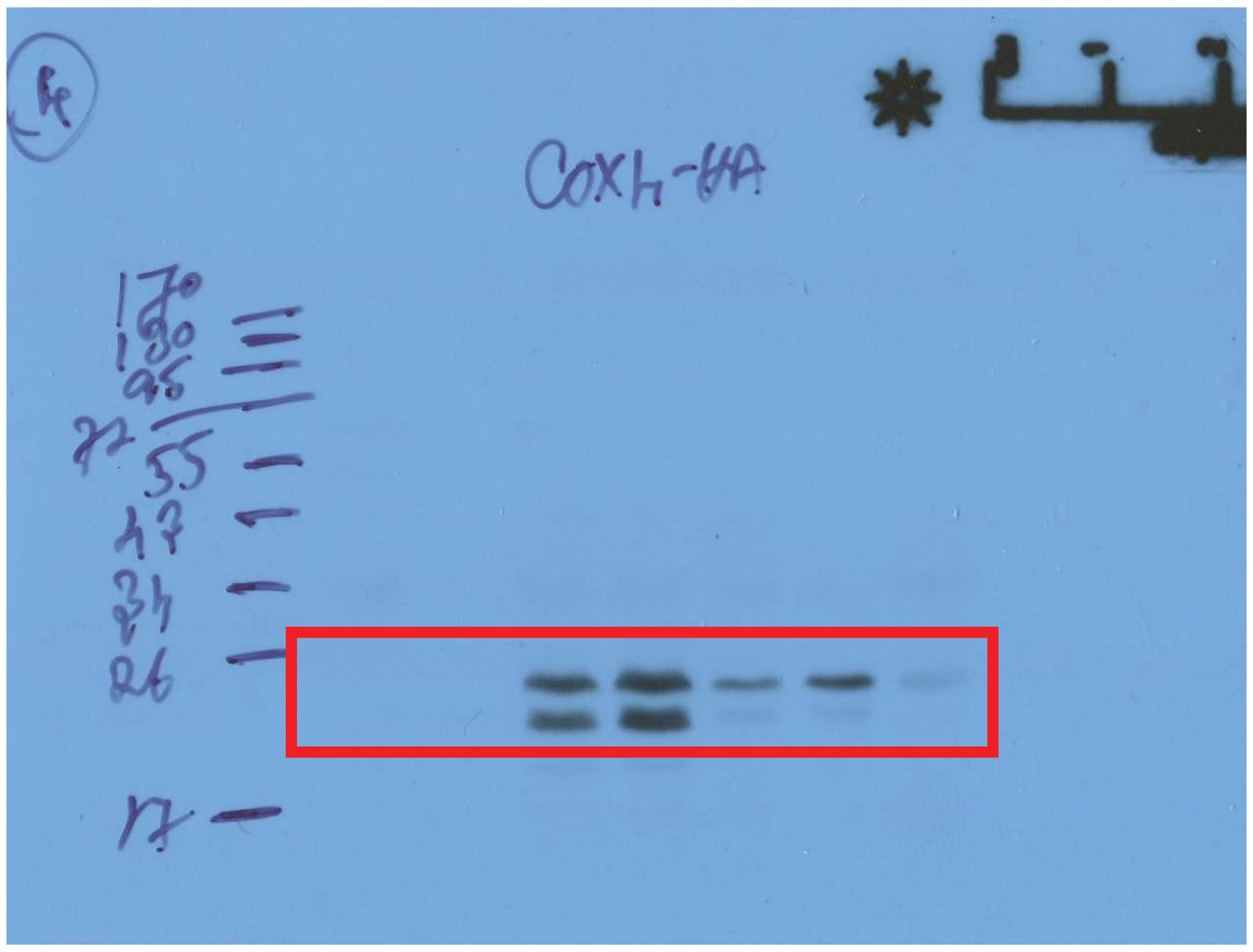

COX4-HA - long

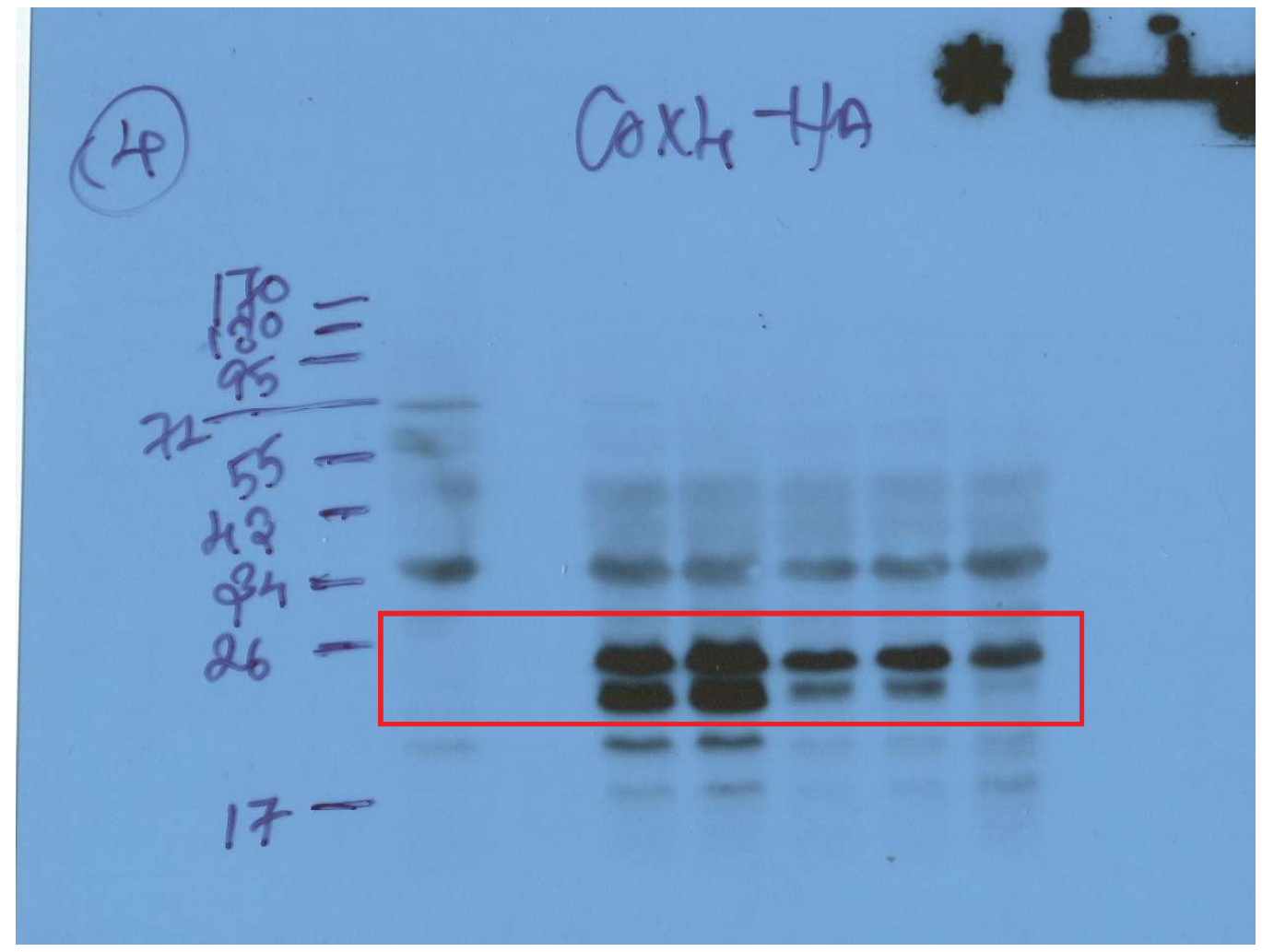

G6PDH for COX4-HA

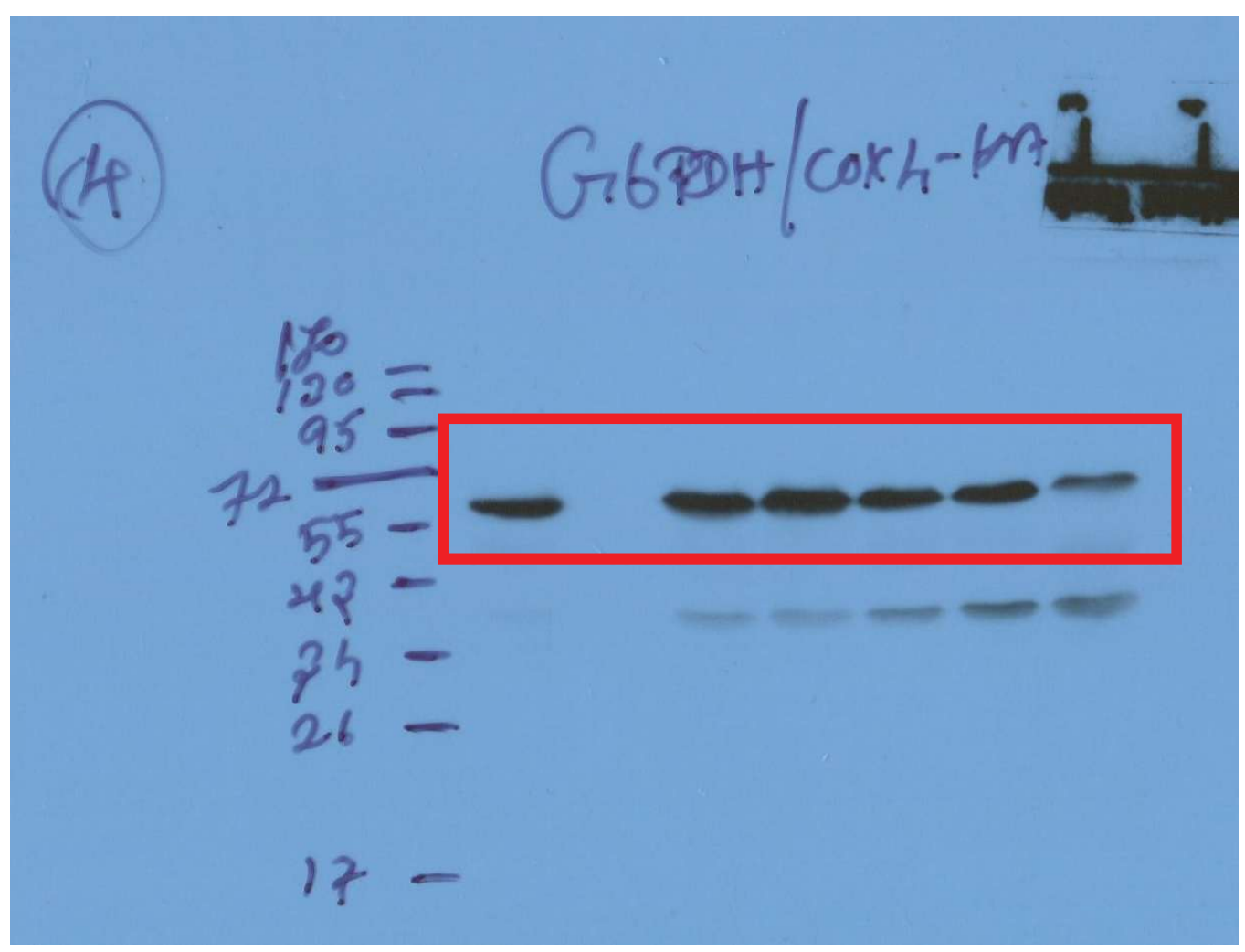

Sup. Fig. 1B

H3

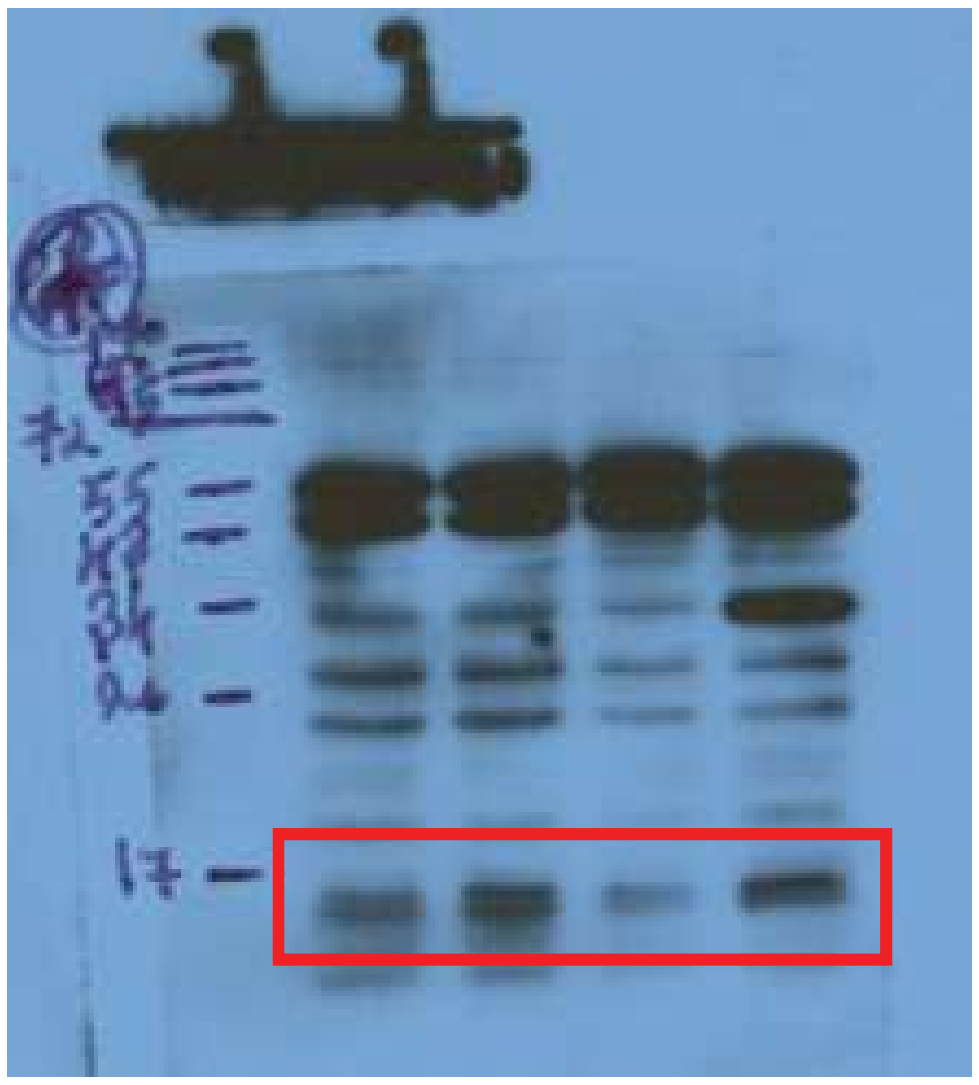

Tubulin for H3

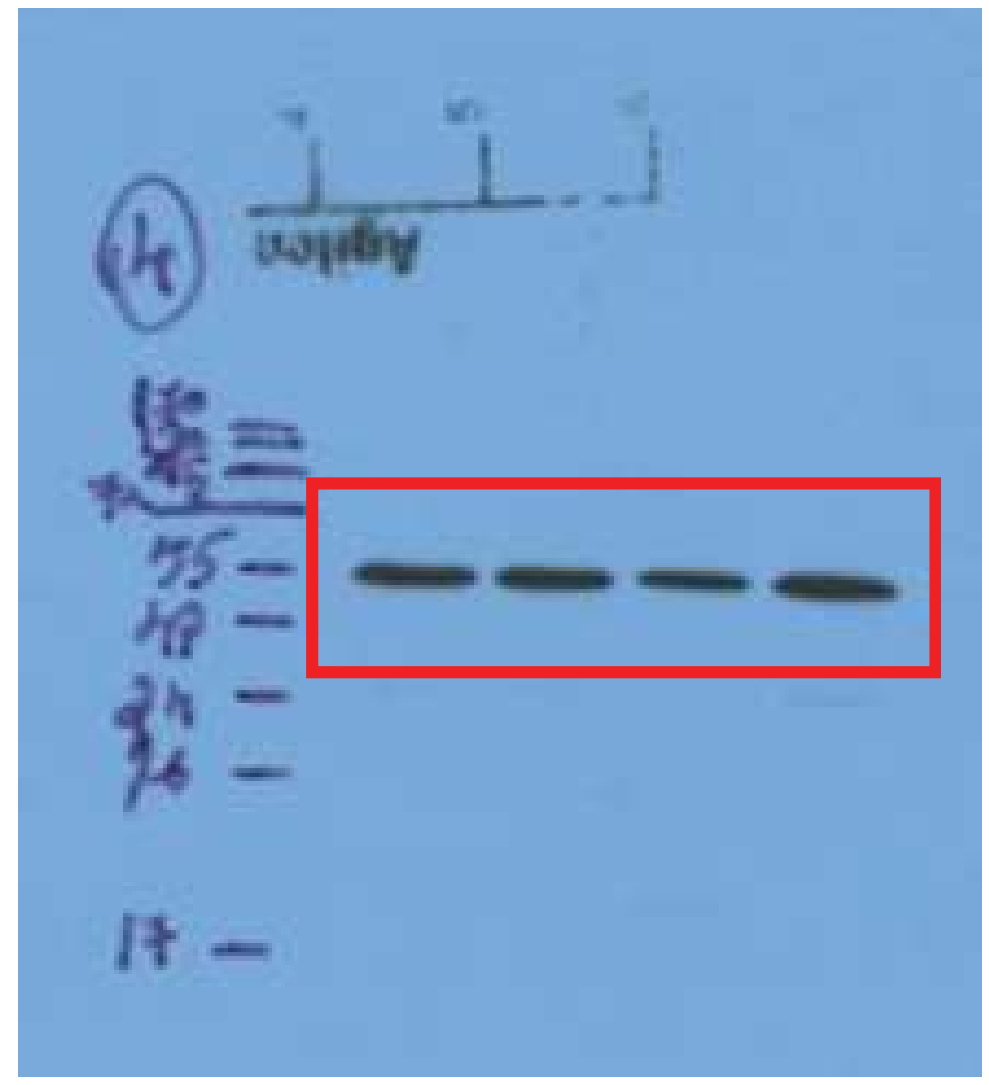

H4

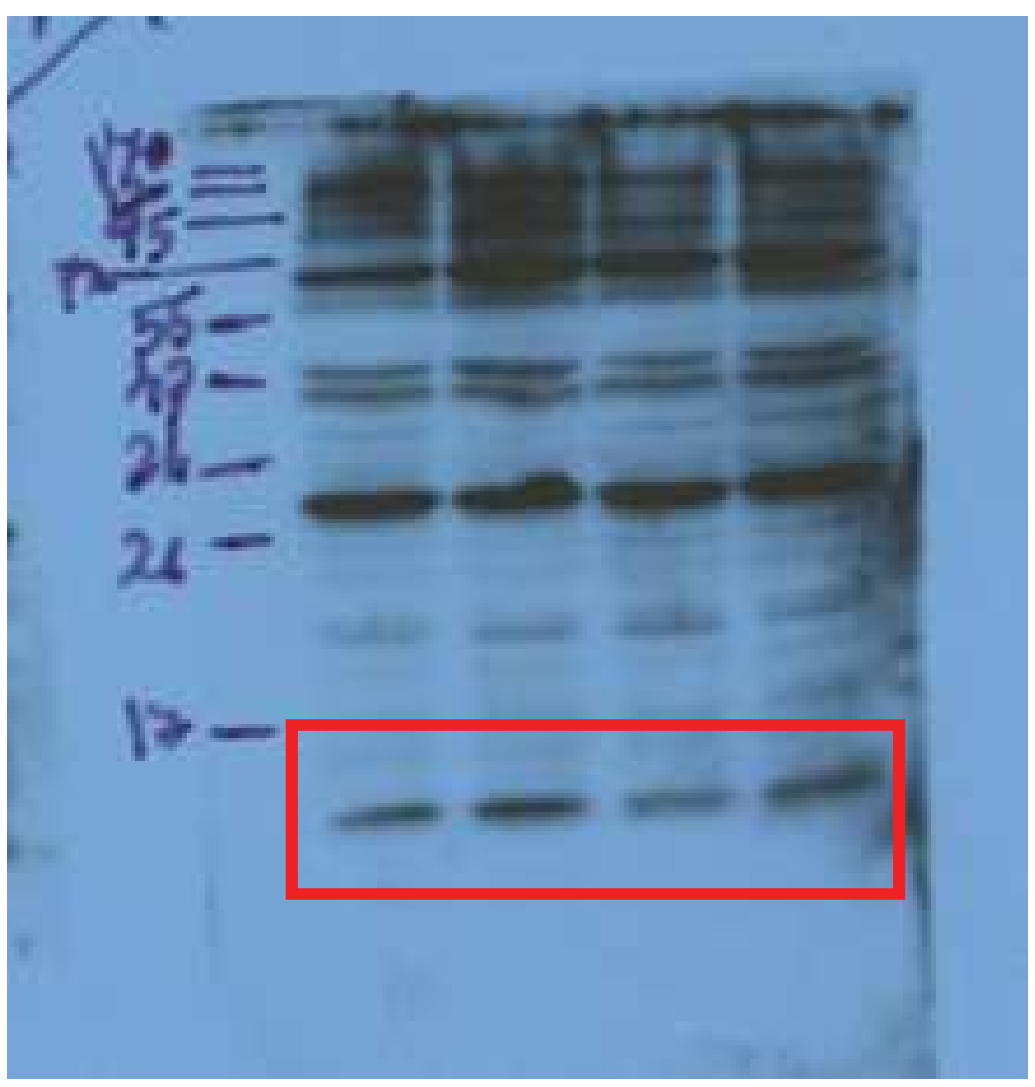

Tubulin for H4

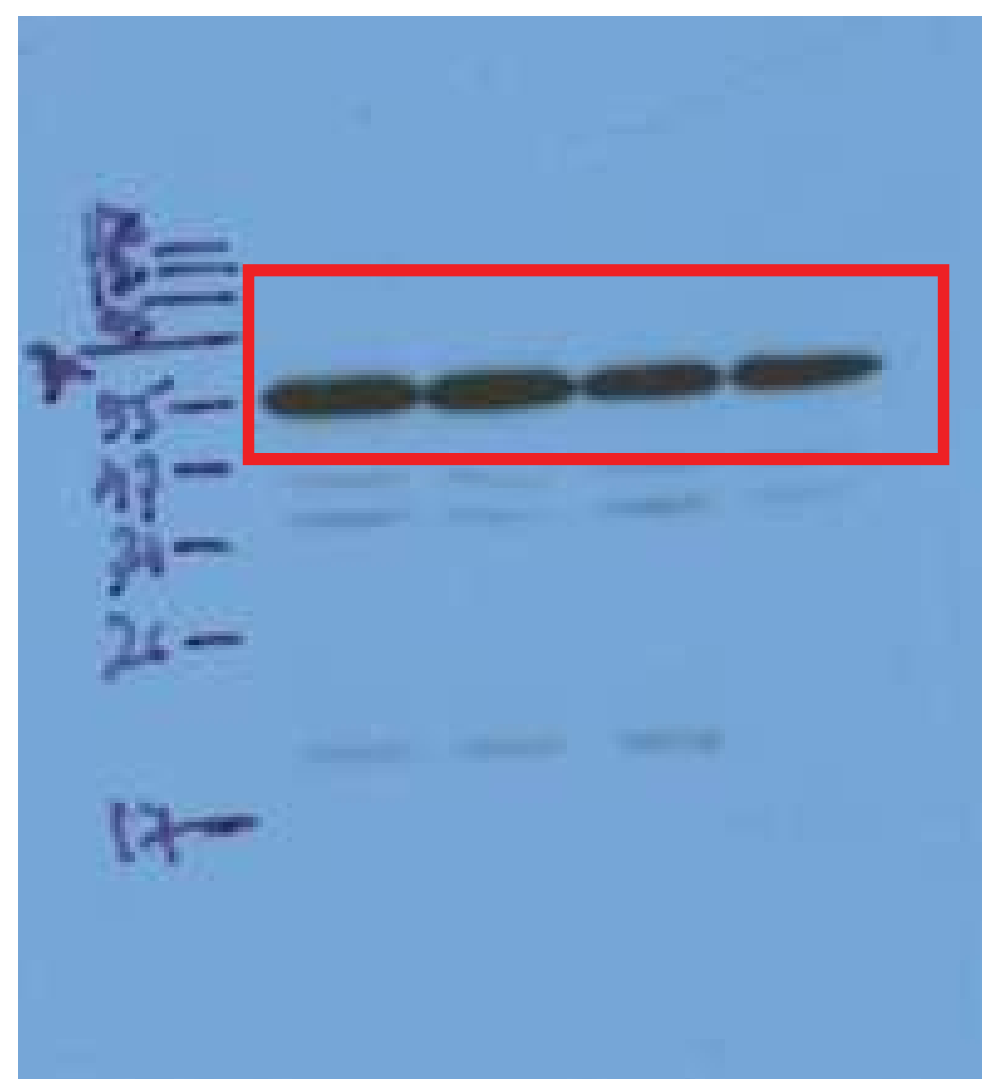

H2A

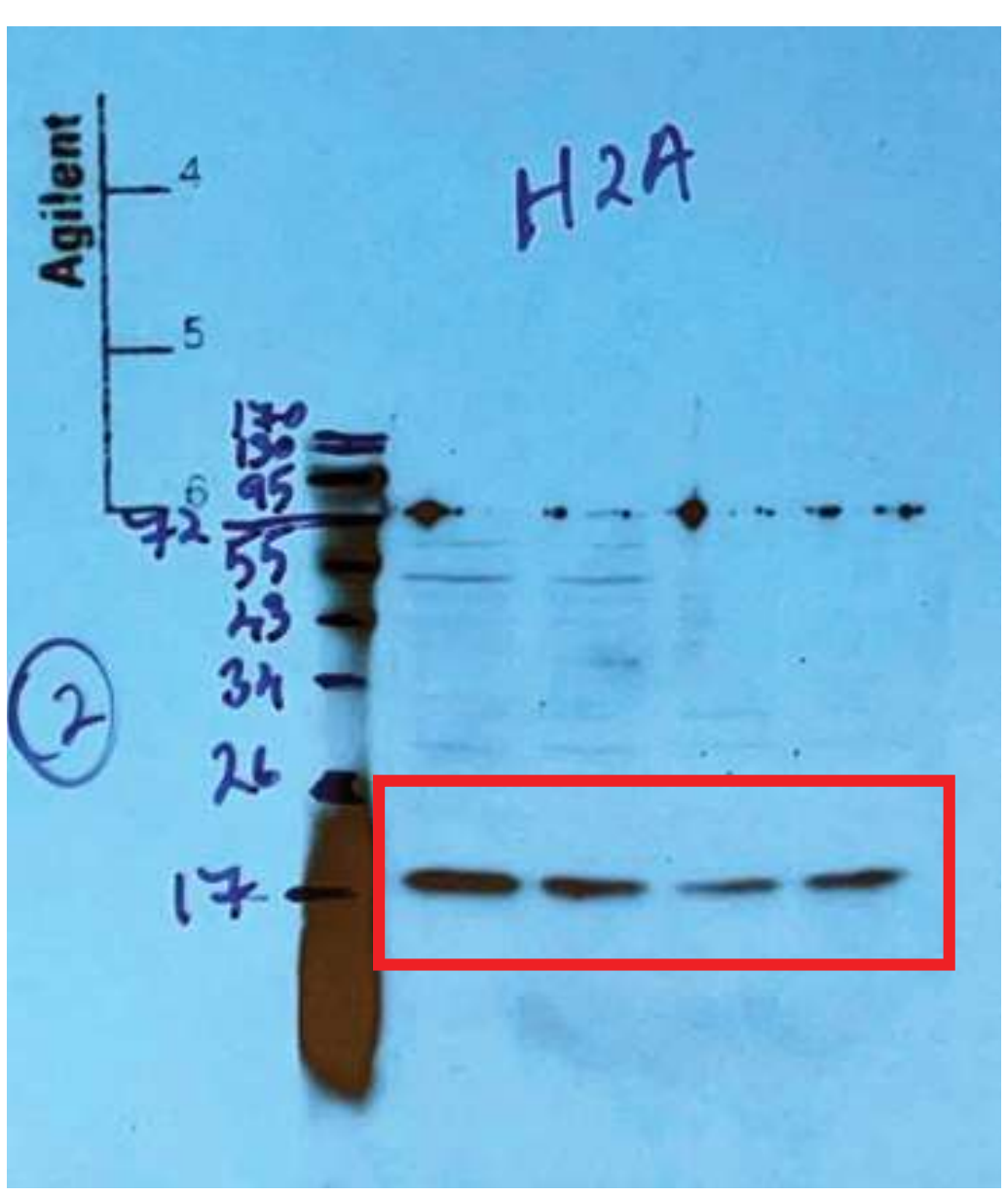

Tubulin for H2A

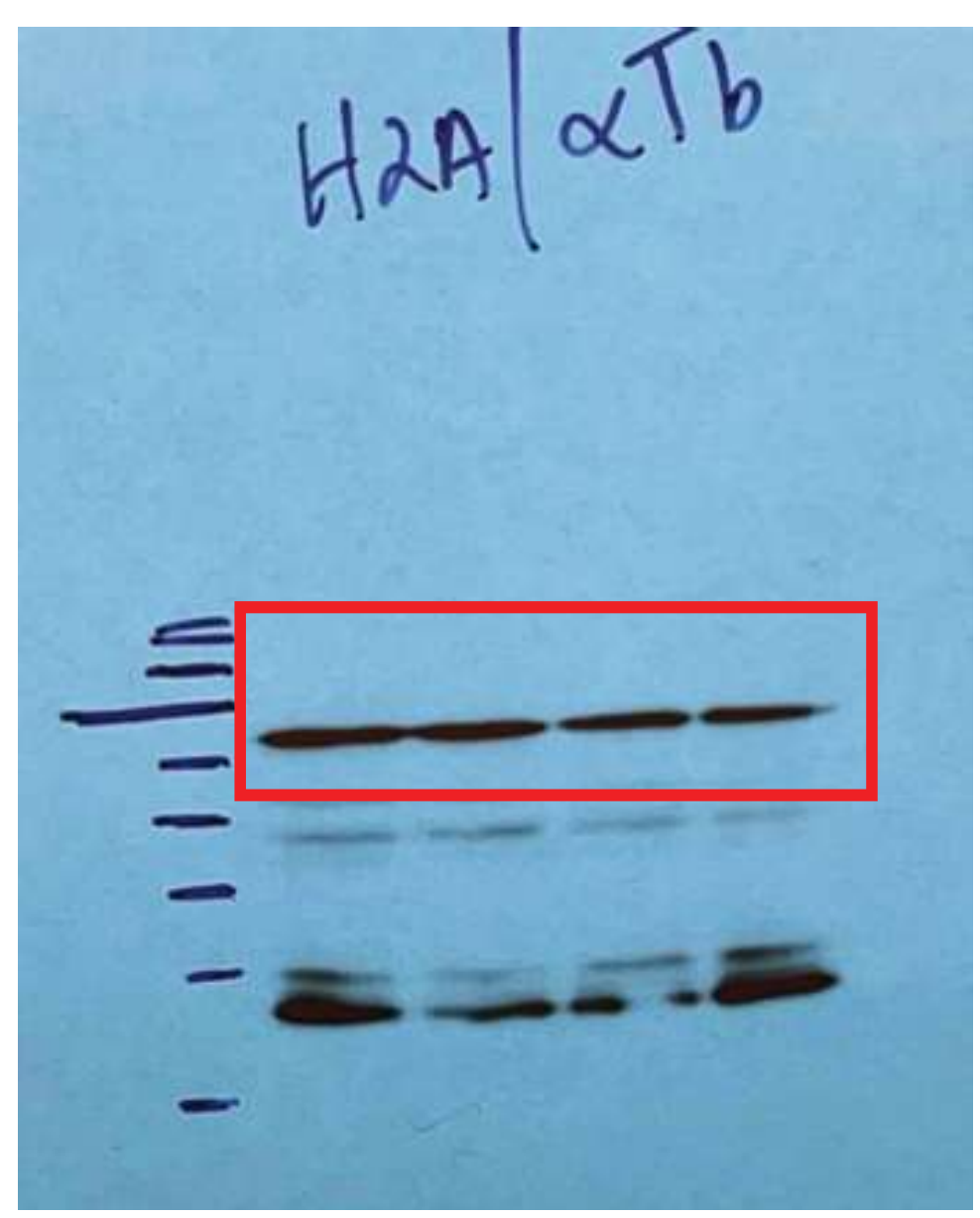

Sup. Fig. 3B

H3

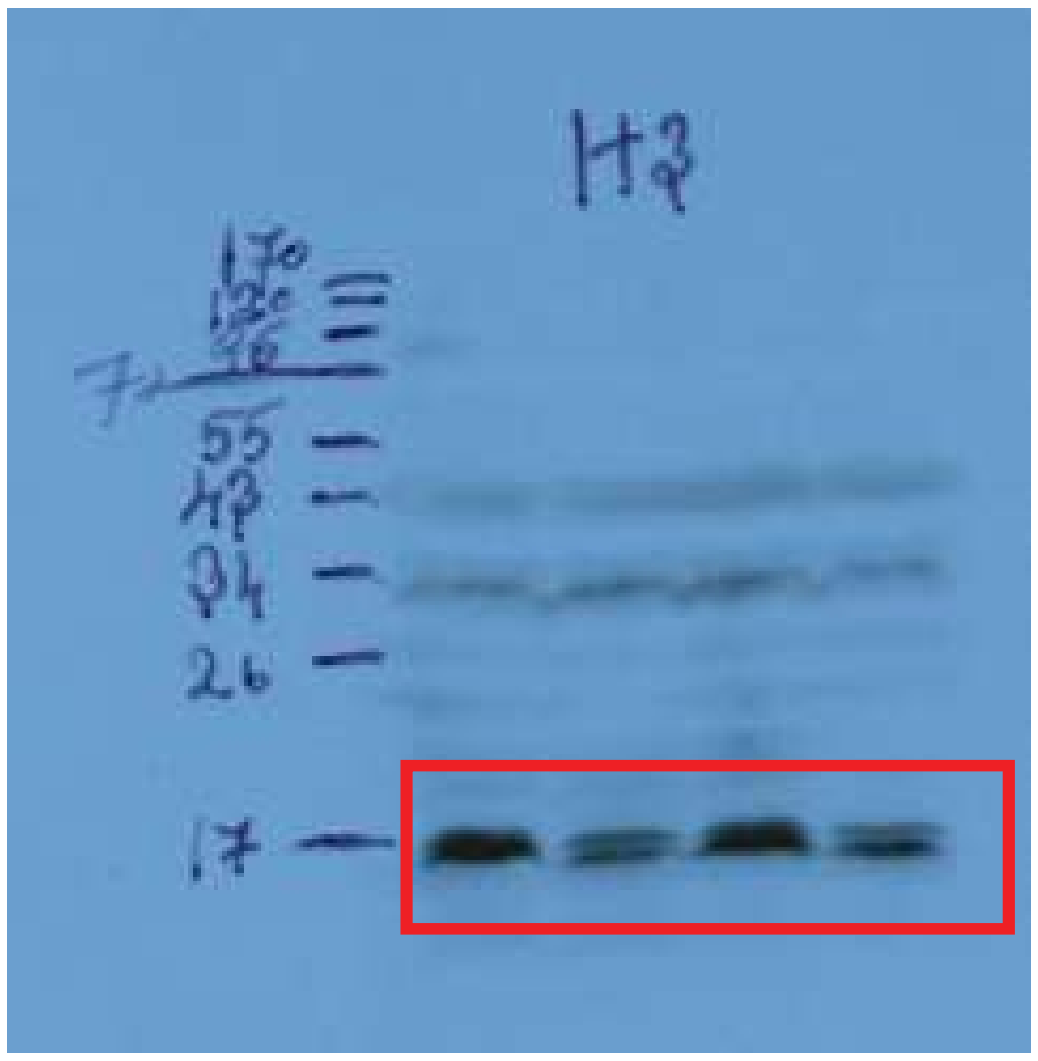

G6PDH for H3

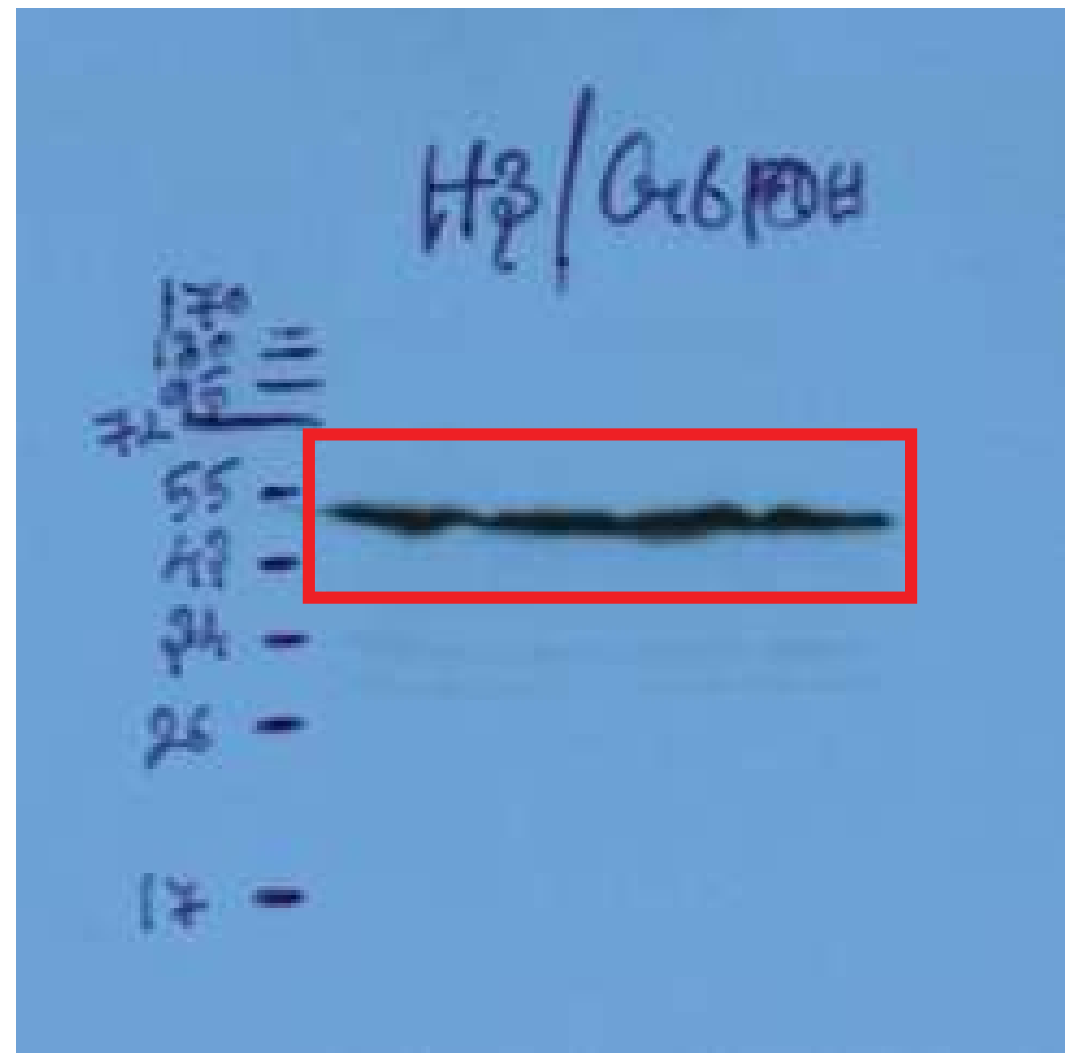

Sup. Fig. 3D

H3K36me3 / H3

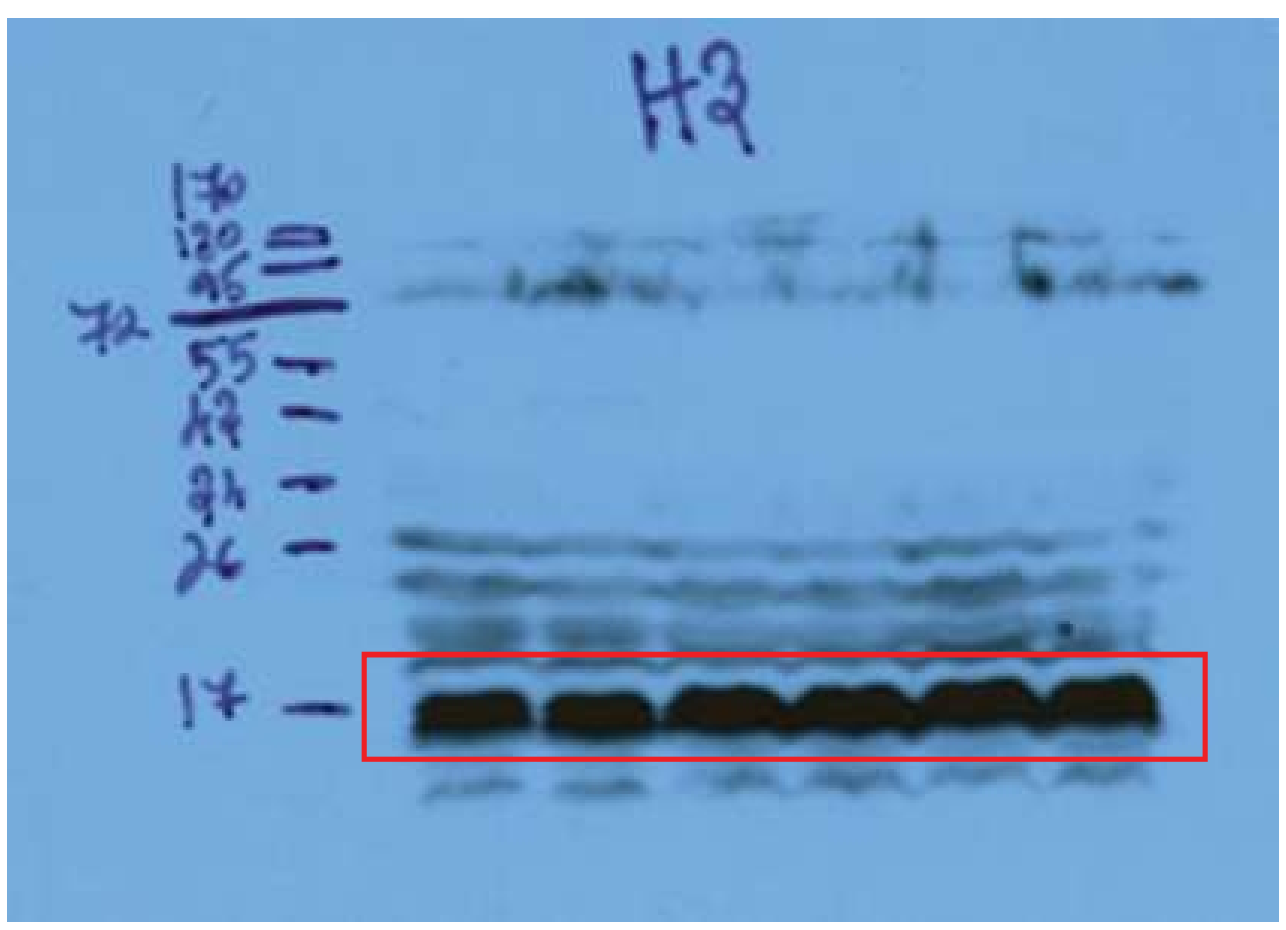

H3K36me3

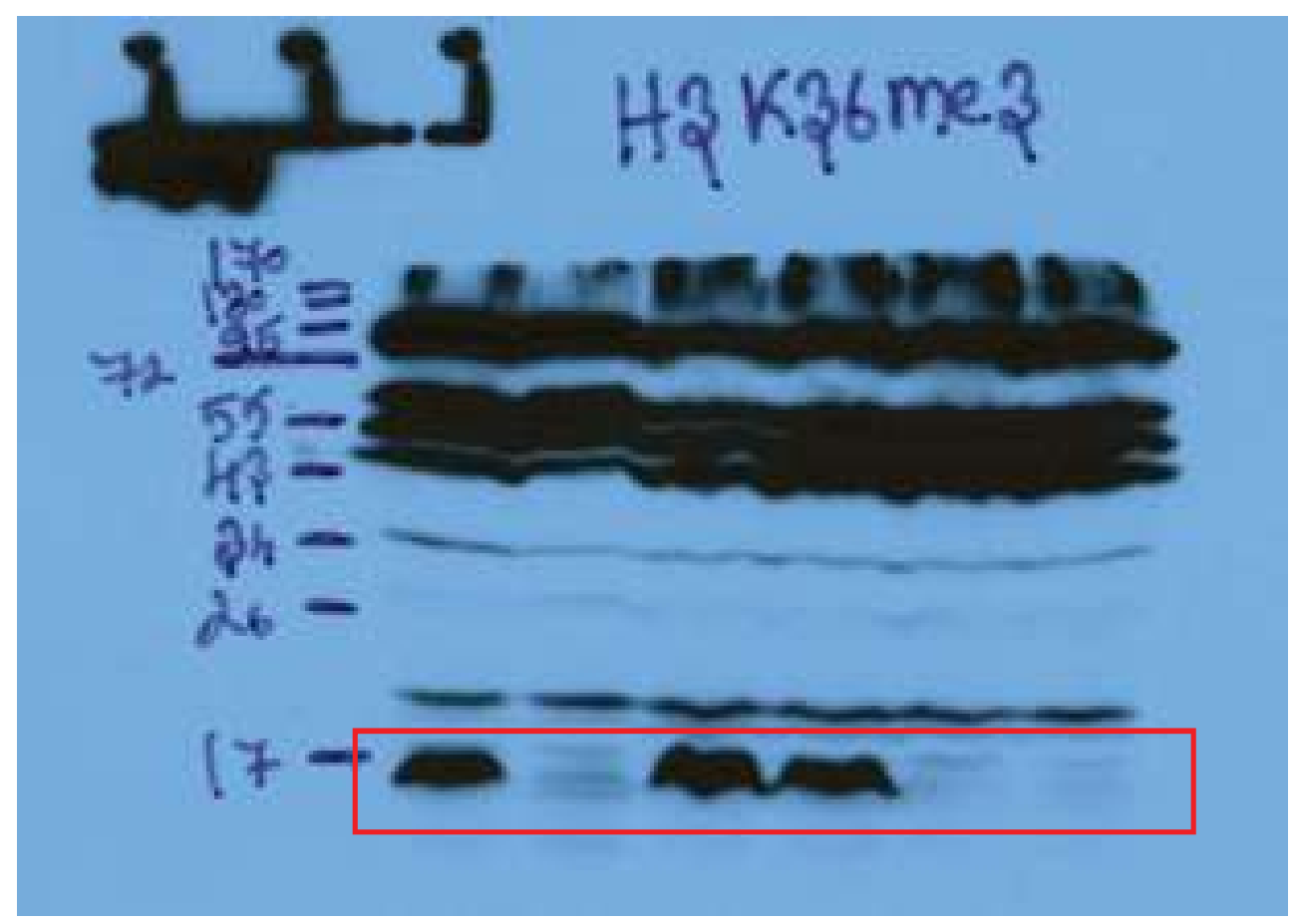

H3K36me2 / H3

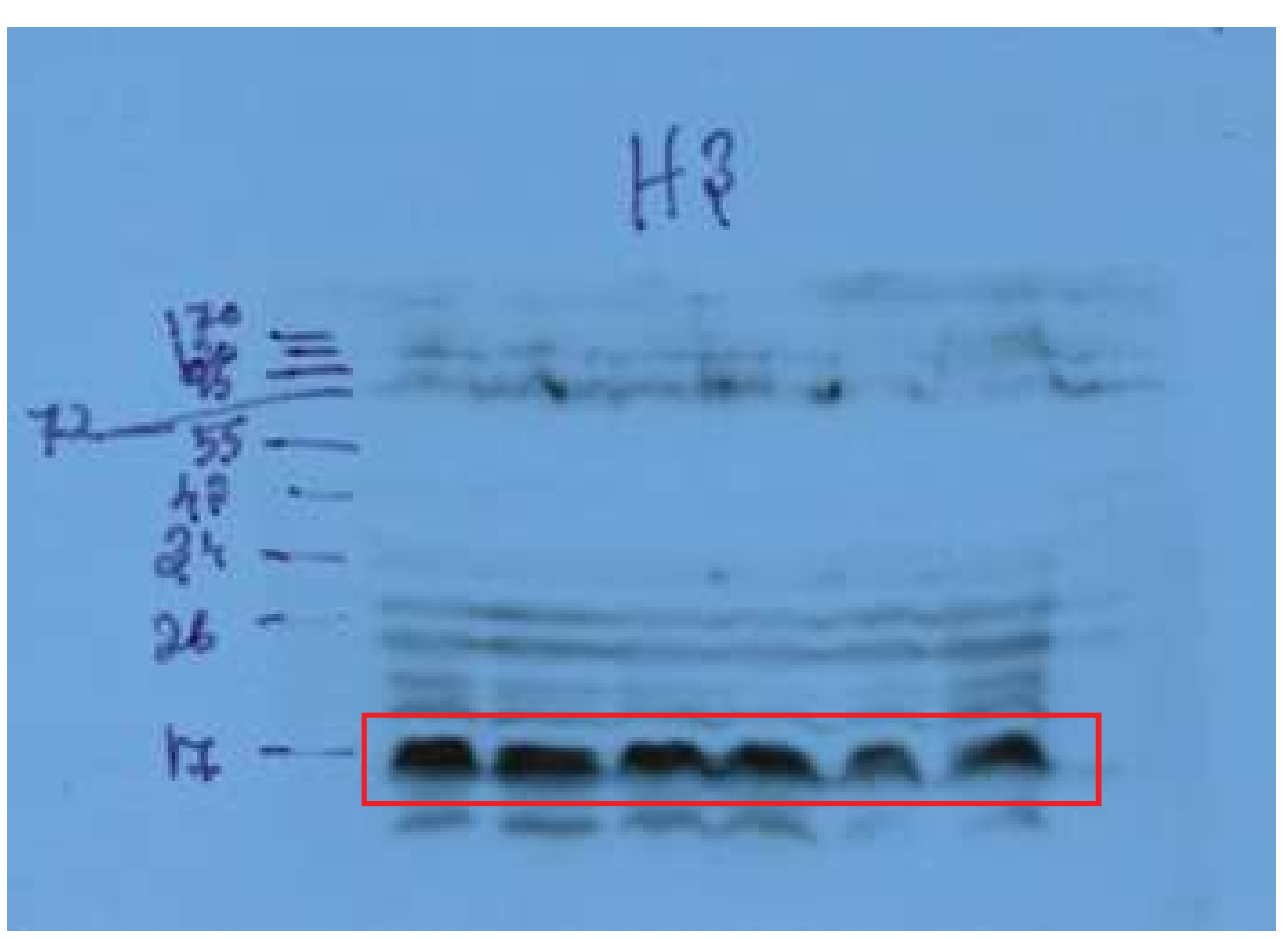

H3K36me2

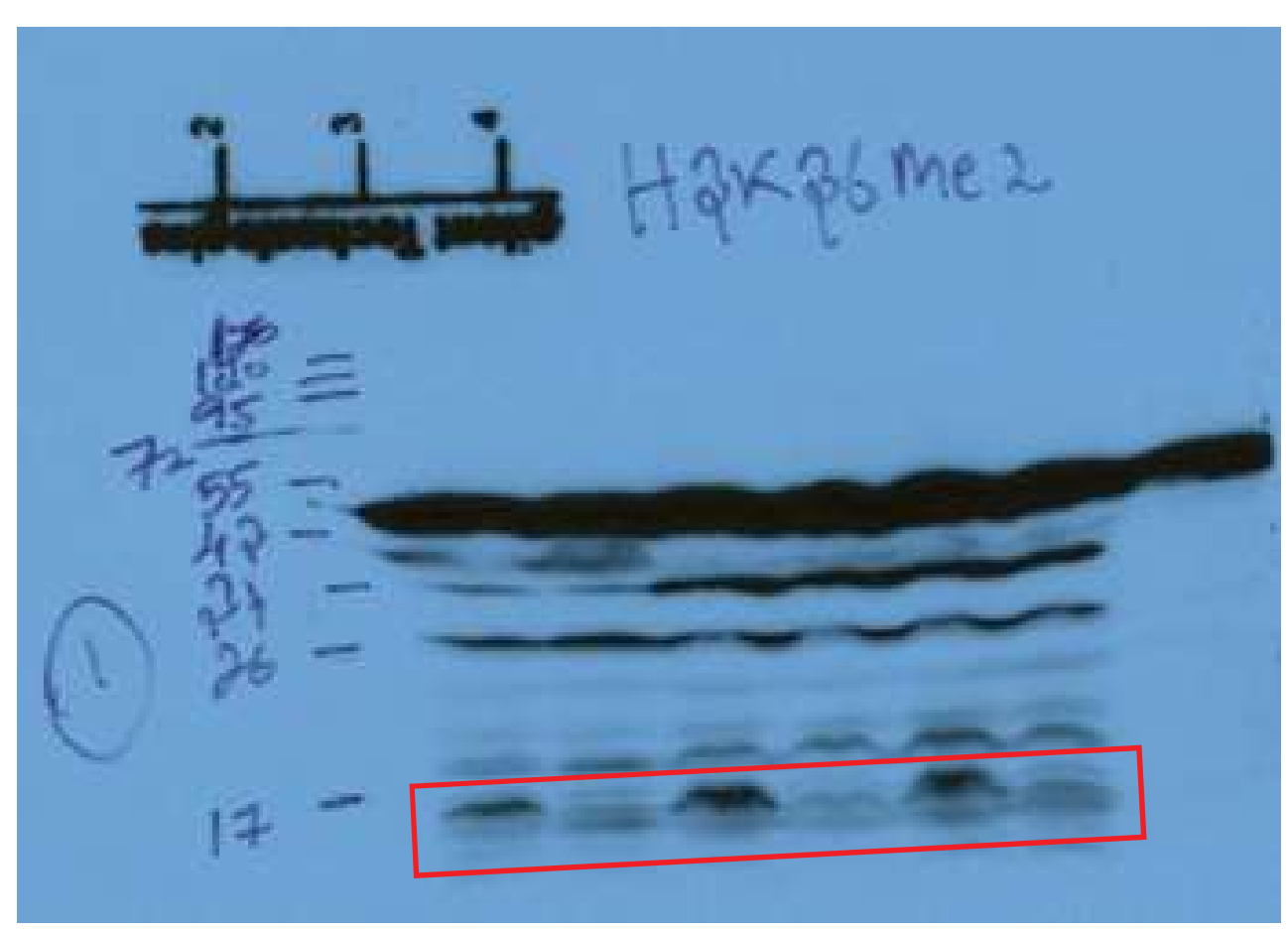

Sup. Fig. 3E

H3K23Ac

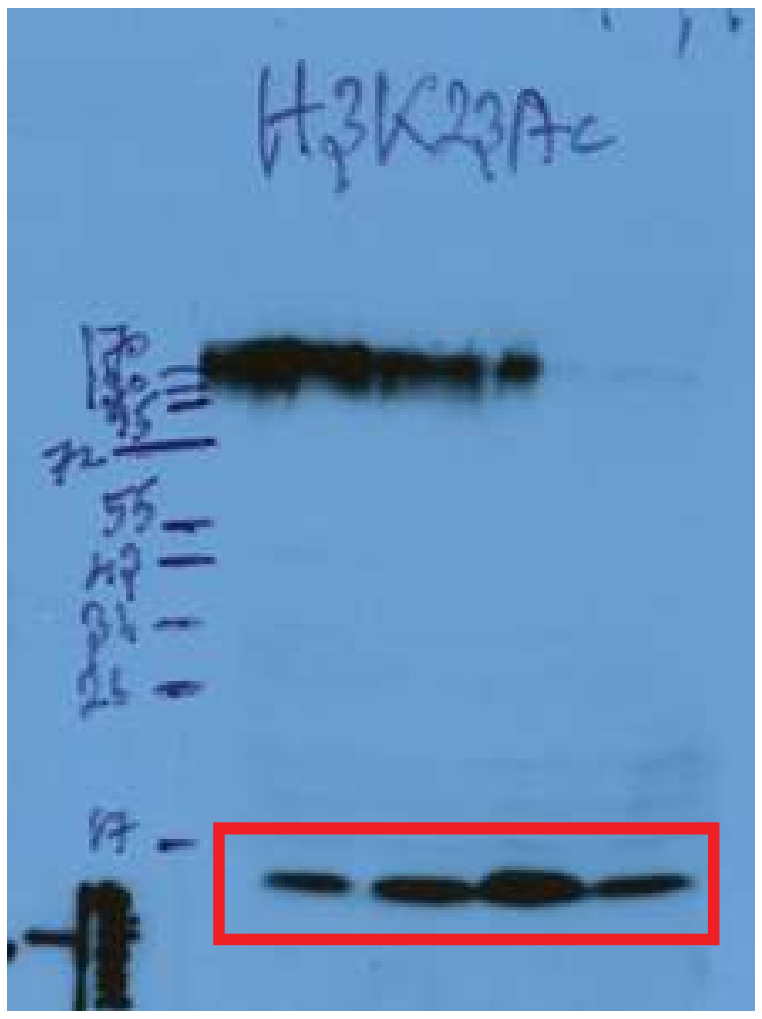

G6PDH for H3K23Ac

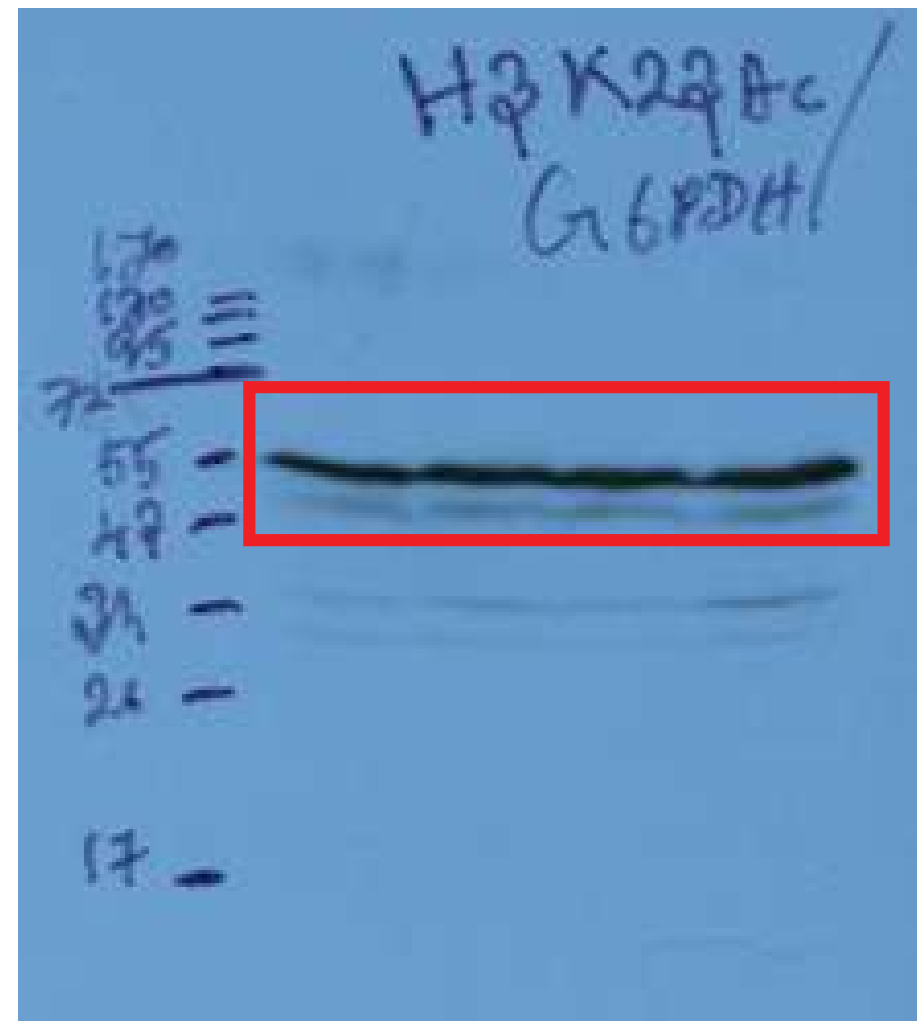

H3

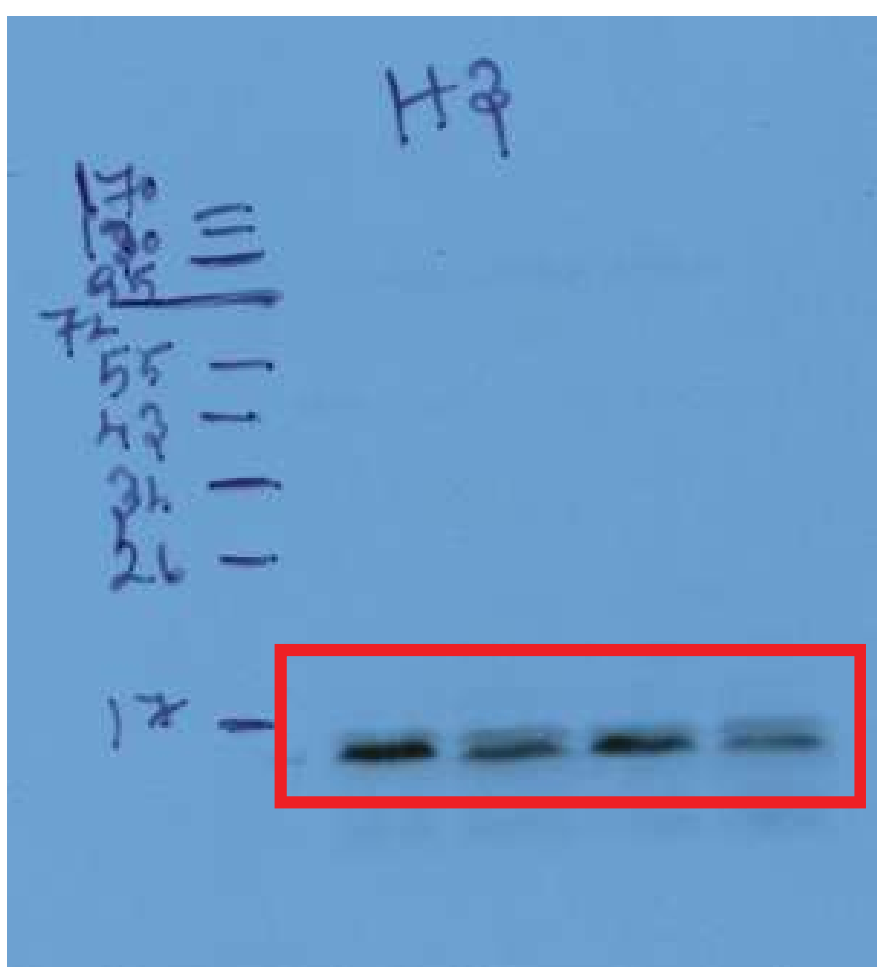

G6PDH for H3

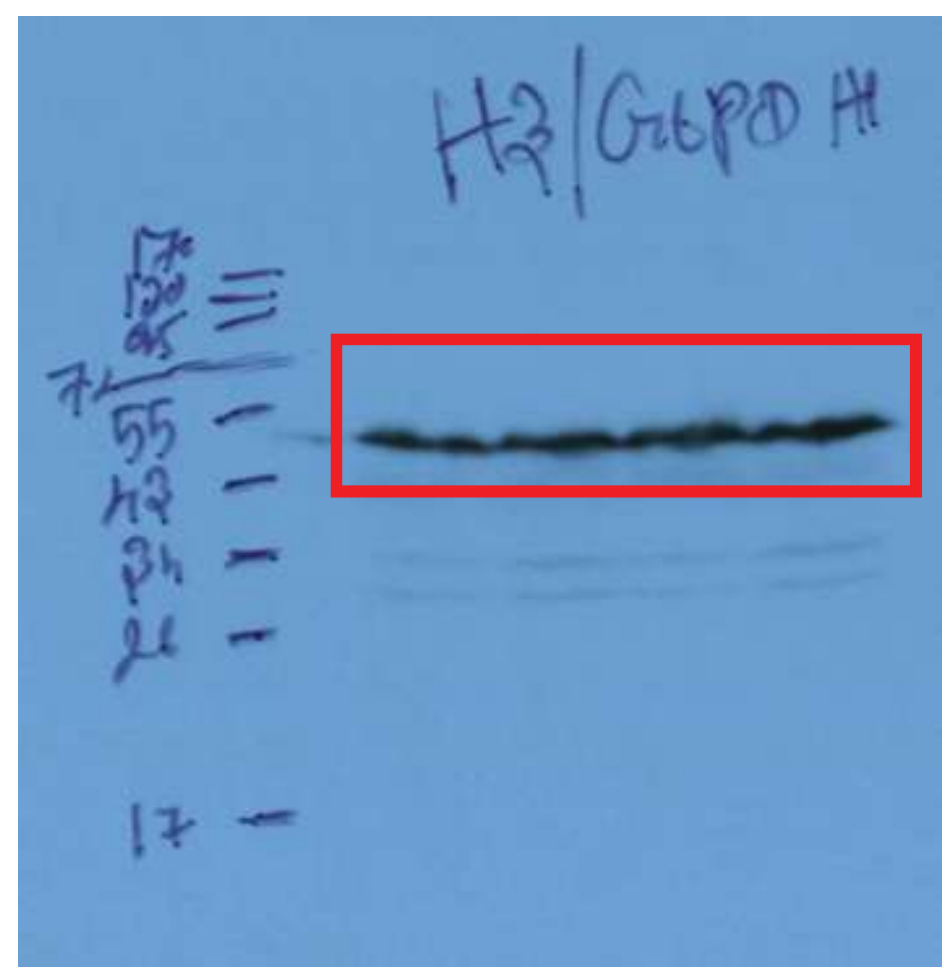

Sup. Fig. 12B

COX4-HA - Short

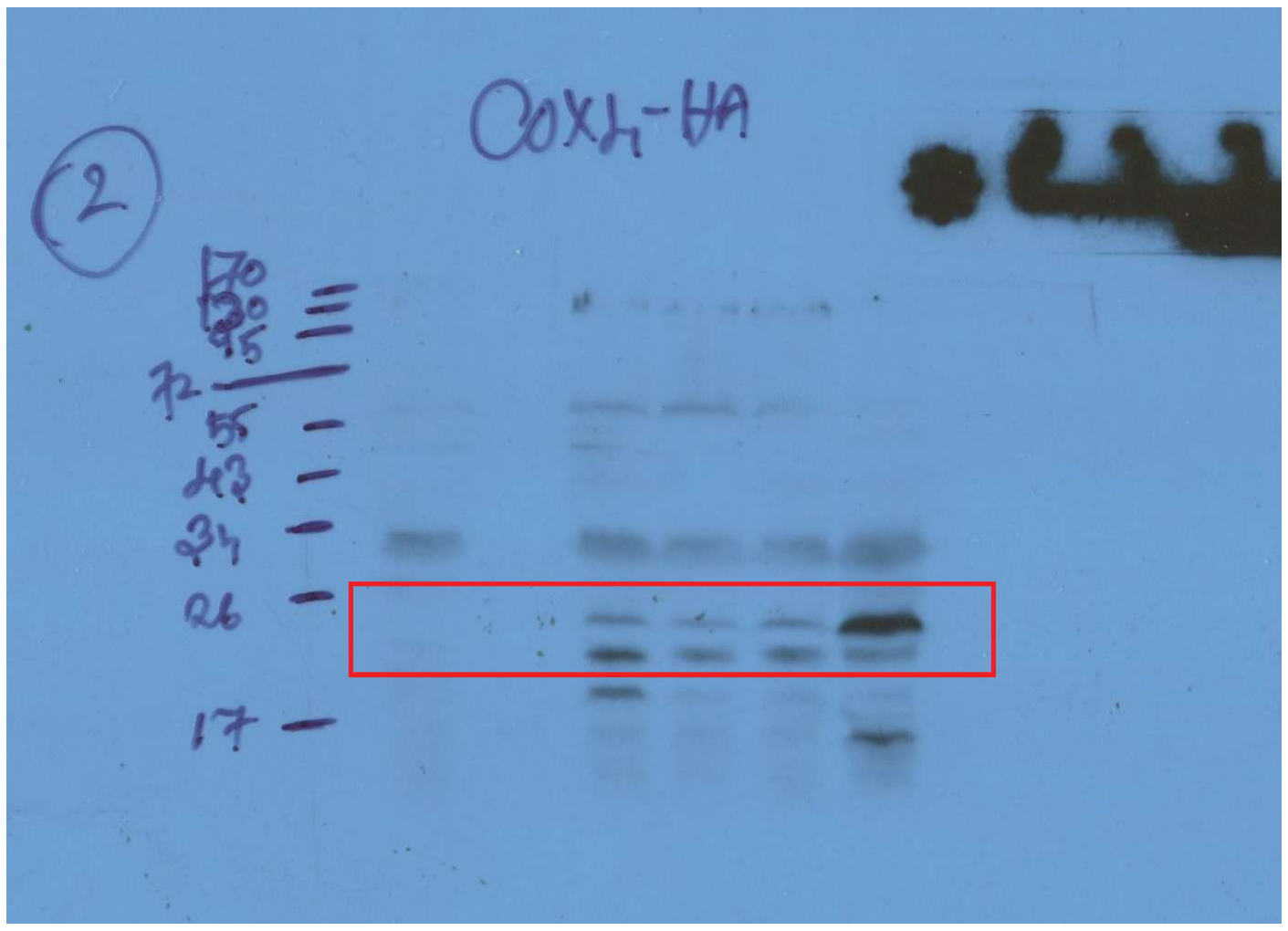

COX4-HA - long

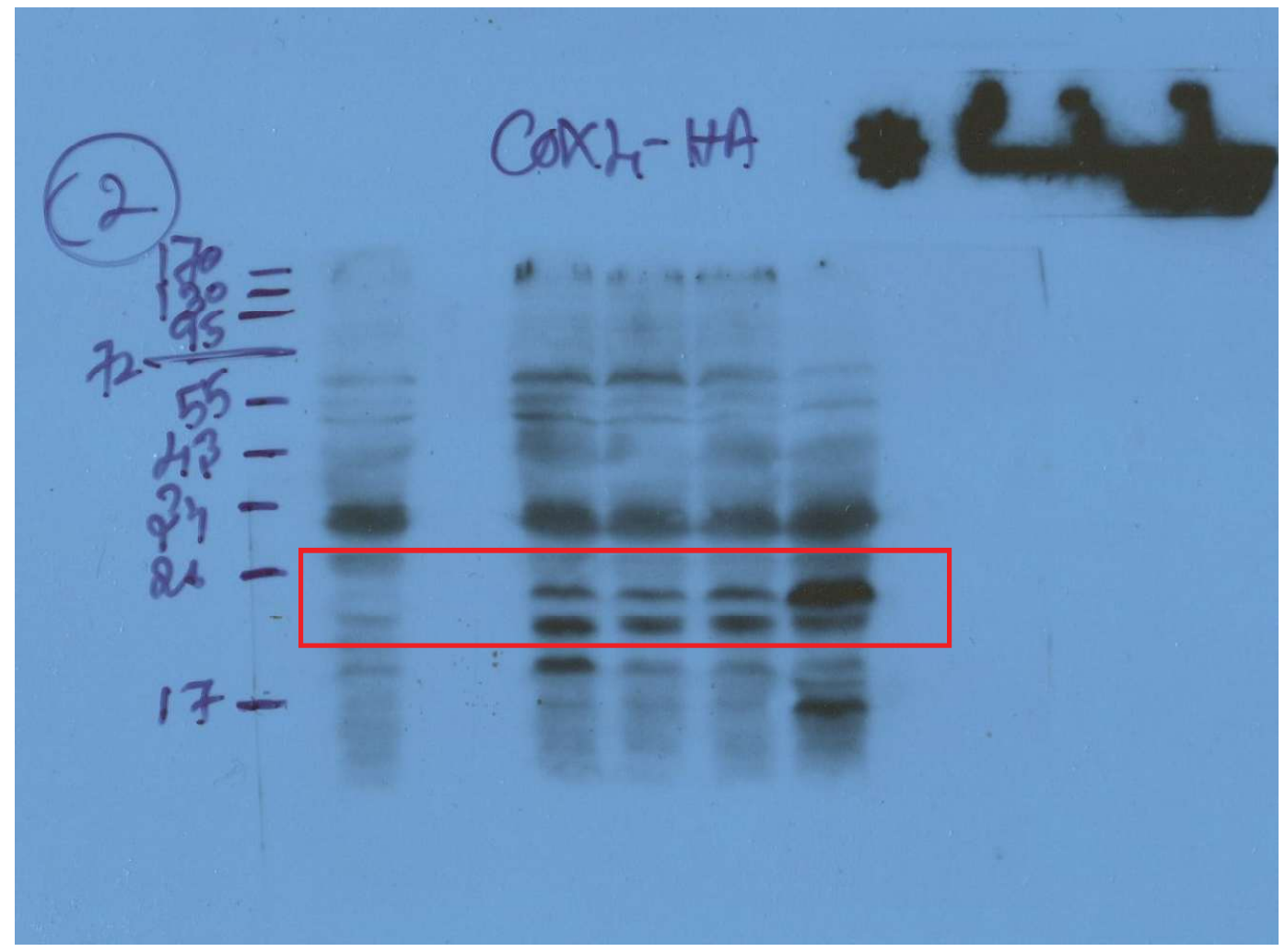

G6PDH for COX4-HA

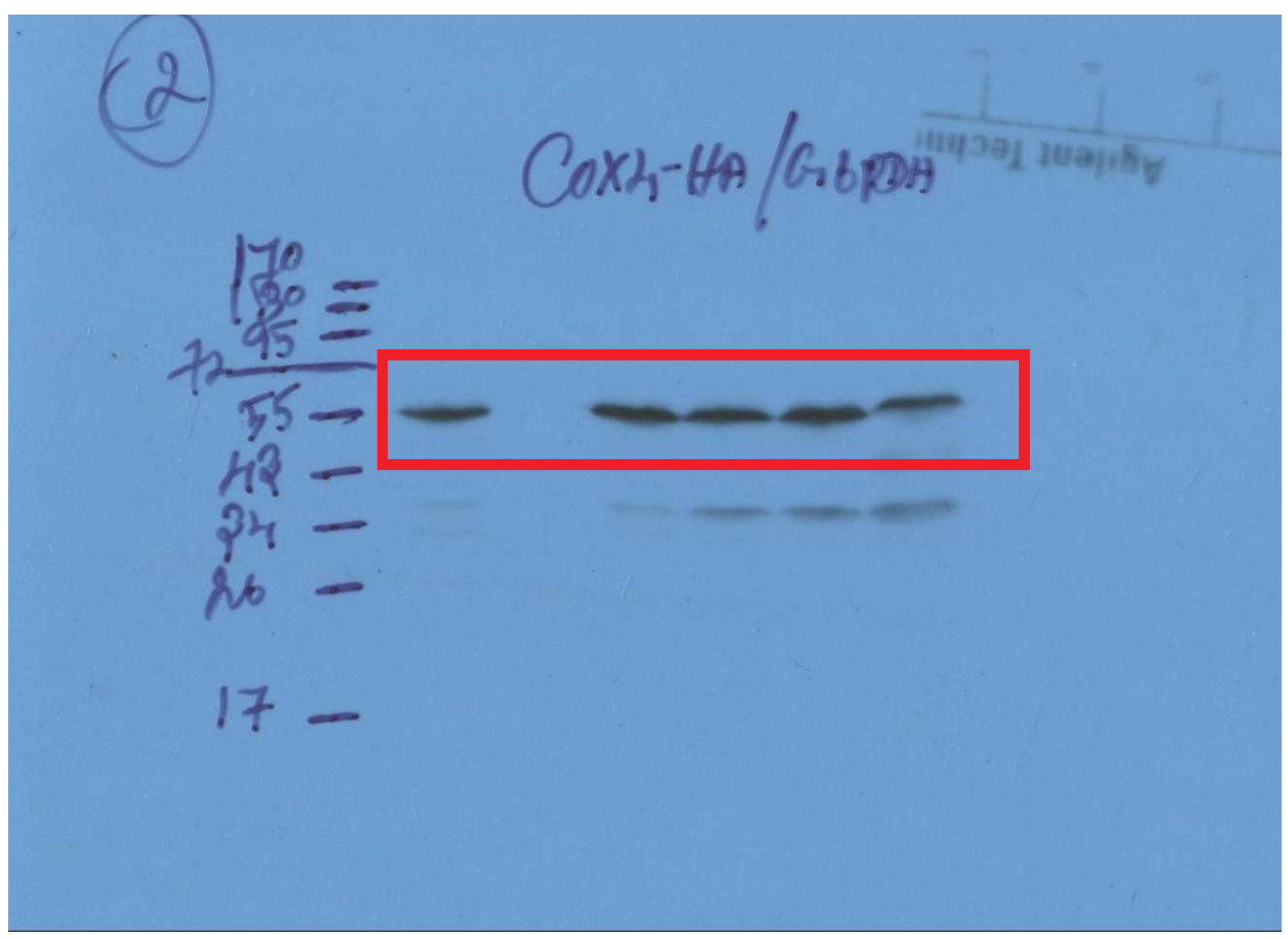

Sup. Fig. 12E

S6ph

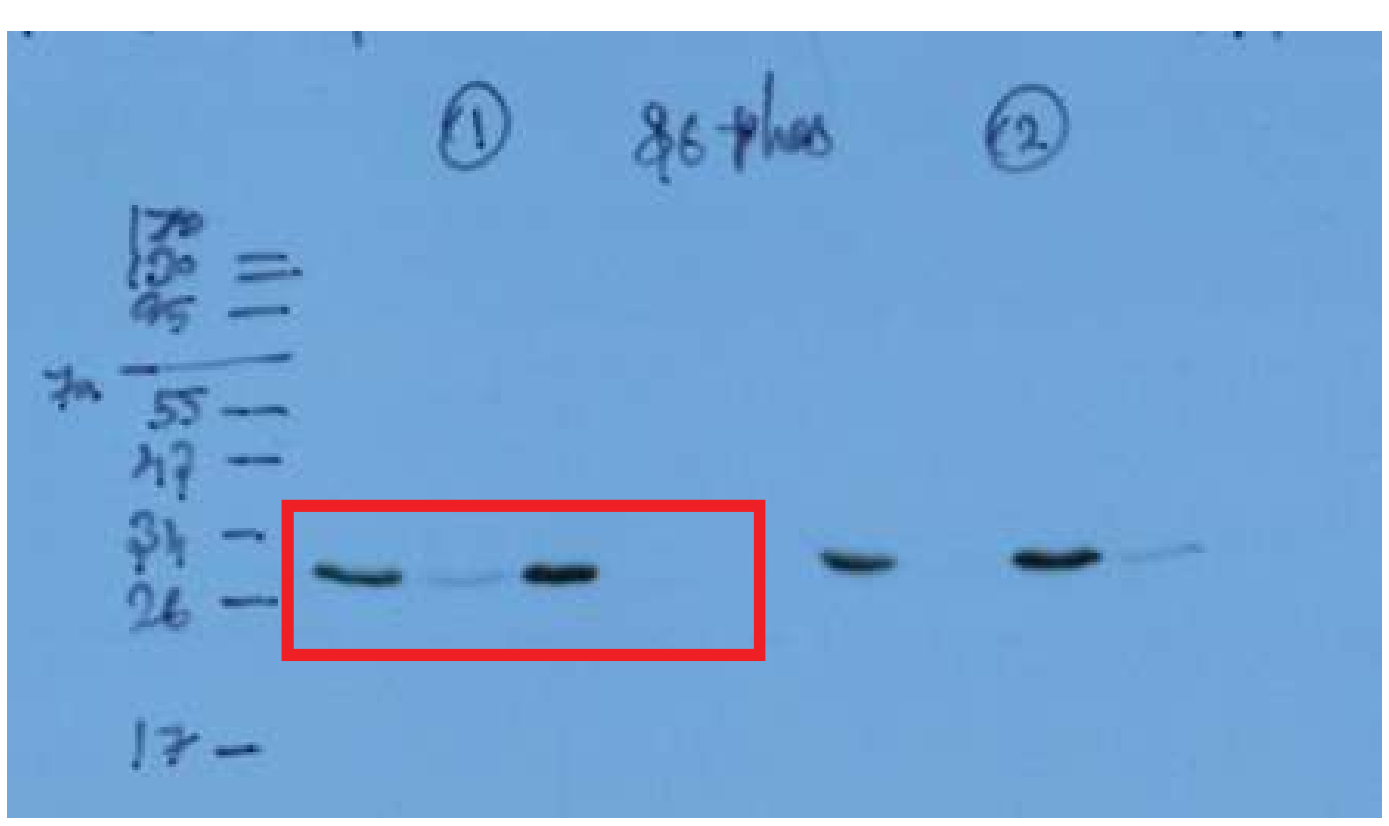

S6 - Total

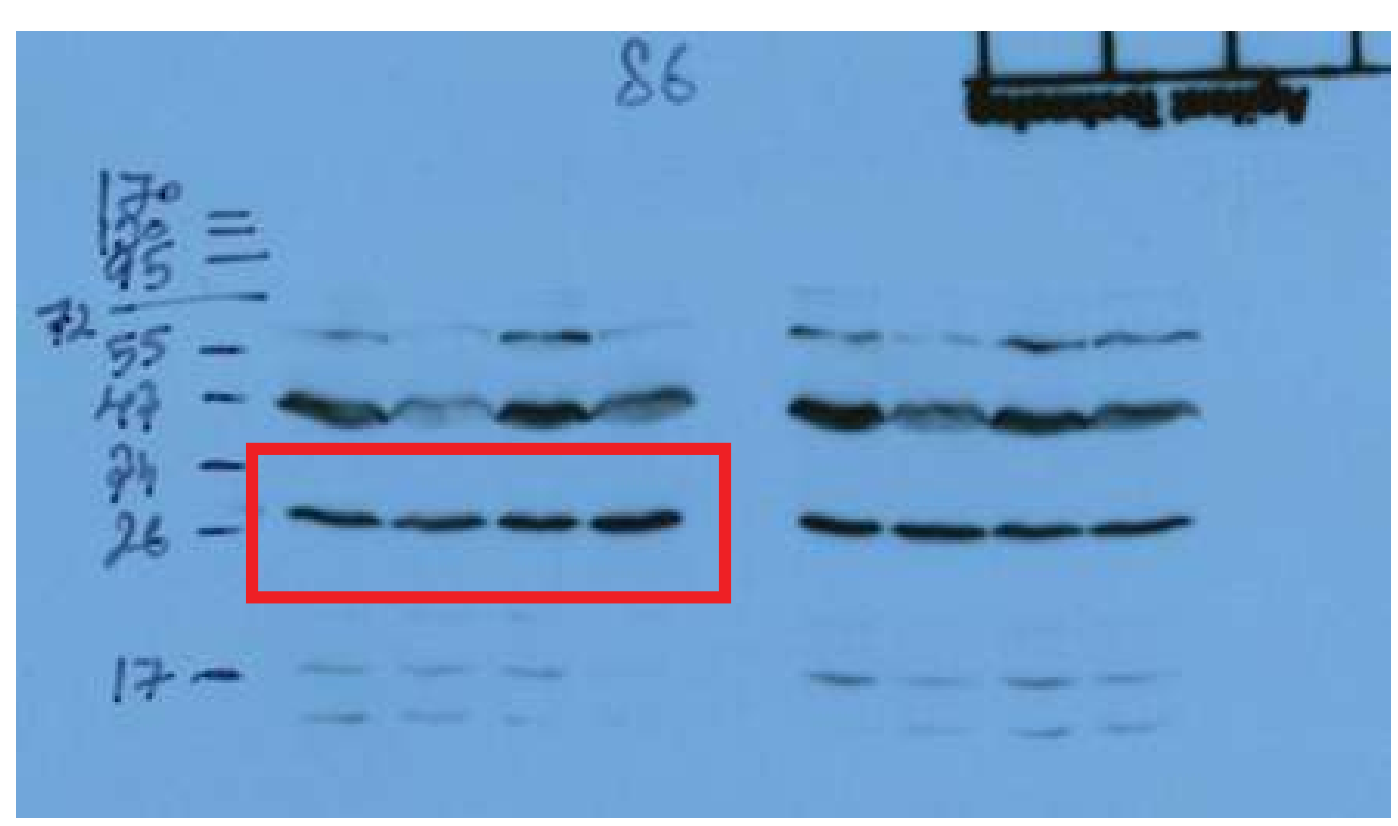

Supplement: Supplementary file 1 [file pgen.1012330.s013.pdf]
