# Supplementary material for: Chromatin stability safeguards mitochondrial homeostasis and restrains TORC1 signaling
Source: PLoS Genet. 2026 Oct 8;22(10):e1012330. doi: 10.1371/journal.pgen.1012330 (PMC13667920; doi:10.1371/journal.pgen.1012330)
Supplement: Supplementary file 2 [file pgen.1012330.s016.docx]

**S3 Table – List of yeast strains**

| **Strain** | **Genotype** | **Source** |
| --- | --- | --- |
| BY4741 | MATa *his3Δ1 leu2Δ0 met15Δ0 ura3Δ0* BY4741 | OPEN Bio |
| H3WT | MATa *his3Δ200 leu2Δ0 lys2Δ0 trp1Δ63 ura3Δ0met15Δ0 can1::MFA1pr-HIS3 hht1-hhf1::NatMX4 hht2-hhf2::[HHTS-HHFS]- ura3Δ* H3WT | OPEN Bio |
| H3K37A | MATa *his3Δ200 leu2Δ0 lys2Δ0 trp1Δ63 ura3Δ0met15Δ0 can1::MFA1pr-HIS3 hht1-hhf1::NatMX4 hht2-hhf2::[HHTS-HHFS]- ura3Δ* H3K37A | OPEN Bio |
| YNL584 | MATa *his3Δ200 leu2Δ0 lys2Δ0 trp1Δ63 ura3Δ0met15Δ0 can1::MFA1pr-HIS3 hht1-hhf1::NatMX4 hht2-hhf2::[HHTS-HHFS]- ura3Δ* H3WT *set2∆::KanMX* | This Study |
| YNL582 | MATa *his3Δ200 leu2Δ0 lys2Δ0 trp1Δ63 ura3Δ0met15Δ0 can1::MFA1pr-HIS3 hht1-hhf1::NatMX4 hht2-hhf2::[HHTS-HHFS]- ura3Δ* H3K37A *set2∆::KanMX* | This Study |
| YNL1119 | MATa *his3Δ200 leu2Δ0 lys2Δ0 trp1Δ63 ura3Δ0met15Δ0 can1::MFA1pr-HIS3 hht1-hhf1::NatMX4 hht2-hhf2::[HHTS-HHFS]- ura3Δ* H3WT *cps50∆::KanMX* | This Study |
| YNL1121 | MATa *his3Δ200 leu2Δ0 lys2Δ0 trp1Δ63 ura3Δ0met15Δ0 can1::MFA1pr-HIS3 hht1-hhf1::NatMX4 hht2-hhf2::[HHTS-HHFS]- ura3Δ* H3K37A *cps50∆::KanMX* | This Study |
| YNL1128 | MATa *his3Δ200 leu2Δ0 lys2Δ0 trp1Δ63 ura3Δ0met15Δ0 can1::MFA1pr-HIS3 hht1-hhf1::NatMX4 hht2-hhf2::[HHTS-HHFS]- ura3Δ* H3WT *dot1∆::HphNT1* | This Study |
| YNL1130 | MATa *his3Δ200 leu2Δ0 lys2Δ0 trp1Δ63 ura3Δ0met15Δ0 can1::MFA1pr-HIS3 hht1-hhf1::NatMX4 hht2-hhf2::[HHTS-HHFS]- ura3Δ* H3K37A *dot1∆::HphNT1* | This Study |
| YNL1051 | MATa *his3Δ200 leu2Δ0 lys2Δ0 trp1Δ63 ura3Δ0met15Δ0 can1::MFA1pr-HIS3 hht1-hhf1::NatMX4 hht2-hhf2::[HHTS-HHFS]- ura3Δ* H3WT *rpd3∆::KanMX* | This Study |
| YNL1053 | MATa *his3Δ200 leu2Δ0 lys2Δ0 trp1Δ63 ura3Δ0met15Δ0 can1::MFA1pr-HIS3 hht1-hhf1::NatMX4 hht2-hhf2::[HHTS-HHFS]- ura3Δ* H3K37A *rpd3∆::KanMX* | This Study |
| YNL1037 | MATa *his3Δ200 leu2Δ0 lys2Δ0 trp1Δ63 ura3Δ0met15Δ0 can1::MFA1pr-HIS3 hht1-hhf1::NatMX4 hht2-hhf2::[HHTS-HHFS]- ura3Δ* H3WT *eaf3∆::KanMX* | This Study |
| YNL1046 | MATa *his3Δ200 leu2Δ0 lys2Δ0 trp1Δ63 ura3Δ0met15Δ0 can1::MFA1pr-HIS3 hht1-hhf1::NatMX4 hht2-hhf2::[HHTS-HHFS]- ura3Δ* H3K37A *eaf3∆::KanMX* | This Study |
| YNL646 | MATa *his3Δ200 leu2Δ0 lys2Δ0 trp1Δ63 ura3Δ0met15Δ0 can1::MFA1pr-HIS3 hht1-hhf1::NatMX4 hht2-hhf2::[HHTS-HHFS]- ura3Δ* H3WT *rph1∆::KanMX* | This Study |
| YNL647 | MATa *his3Δ200 leu2Δ0 lys2Δ0 trp1Δ63 ura3Δ0met15Δ0 can1::MFA1pr-HIS3 hht1-hhf1::NatMX4 hht2-hhf2::[HHTS-HHFS]- ura3Δ* H3K37A *rph1∆::KanMX* | This Study |
| YNL1147 | MATa *his3Δ200 leu2Δ0 lys2Δ0 trp1Δ63 ura3Δ0met15Δ0 can1::MFA1pr-HIS3 hht1-hhf1::NatMX4 hht2-hhf2::[HHTS-HHFS]- ura3Δ* H3WT *ioc4∆::HphNT1* | This Study |
| YNL1149 | MATa *his3Δ200 leu2Δ0 lys2Δ0 trp1Δ63 ura3Δ0met15Δ0 can1::MFA1pr-HIS3 hht1-hhf1::NatMX4 hht2-hhf2::[HHTS-HHFS]- ura3Δ* H3K37A *ioc4∆::HphNT1* | This Study |
| YNL1151 | MATa *his3Δ200 leu2Δ0 lys2Δ0 trp1Δ63 ura3Δ0met15Δ0 can1::MFA1pr-HIS3 hht1-hhf1::NatMX4 hht2-hhf2::[HHTS-HHFS]- ura3Δ* H3WT *pdp3∆::HphNT1* | This Study |
| YNL1153 | MATa *his3Δ200 leu2Δ0 lys2Δ0 trp1Δ63 ura3Δ0met15Δ0 can1::MFA1pr-HIS3 hht1-hhf1::NatMX4 hht2-hhf2::[HHTS-HHFS]- ura3Δ* H3K37A *pdp3∆::HphNT1* | This Study |
| YNL1269 | MATa *his3Δ200 leu2Δ0 lys2Δ0 trp1Δ63 ura3Δ0met15Δ0 can1::MFA1pr-HIS3 hht1-hhf1::NatMX4 hht2-hhf2::[HHTS-HHFS]- ura3Δ* H3WT *cac2∆::KanMX* | This Study |
| YNL1271 | MATa *his3Δ200 leu2Δ0 lys2Δ0 trp1Δ63 ura3Δ0met15Δ0 can1::MFA1pr-HIS3 hht1-hhf1::NatMX4 hht2-hhf2::[HHTS-HHFS]- ura3Δ* H3K37A *cac2∆::KanMX* | This Study |
| YNL1255 | MATa *his3Δ200 leu2Δ0 lys2Δ0 trp1Δ63 ura3Δ0met15Δ0 can1::MFA1pr-HIS3 hht1-hhf1::NatMX4 hht2-hhf2::[HHTS-HHFS]- ura3Δ* H3WT *hir2∆::HphNT1* | This Study |
| YNL1257 | MATa *his3Δ200 leu2Δ0 lys2Δ0 trp1Δ63 ura3Δ0met15Δ0 can1::MFA1pr-HIS3 hht1-hhf1::NatMX4 hht2-hhf2::[HHTS-HHFS]- ura3Δ* H3K37A *hir2∆::HphNT1* | This Study |
| YNL1243 | MATa *his3Δ200 leu2Δ0 lys2Δ0 trp1Δ63 ura3Δ0met15Δ0 can1::MFA1pr-HIS3 hht1-hhf1::NatMX4 hht2-hhf2::[HHTS-HHFS]- ura3Δ* H3WT *rtg1∆::HphNT1* | This Study |
| YNL1245 | MATa *his3Δ200 leu2Δ0 lys2Δ0 trp1Δ63 ura3Δ0met15Δ0 can1::MFA1pr-HIS3 hht1-hhf1::NatMX4 hht2-hhf2::[HHTS-HHFS]- ura3Δ* H3K37A *rtg1∆::HphNT1* | This Study |
| YNL1249 | MATa *his3Δ200 leu2Δ0 lys2Δ0 trp1Δ63 ura3Δ0met15Δ0 can1::MFA1pr-HIS3 hht1-hhf1::NatMX4 hht2-hhf2::[HHTS-HHFS]- ura3Δ* H3WT *set2∆::KanMX* *rtg1∆::HphNT1* | This Study |
| YNL1251 | MATa *his3Δ200 leu2Δ0 lys2Δ0 trp1Δ63 ura3Δ0met15Δ0 can1::MFA1pr-HIS3 hht1-hhf1::NatMX4 hht2-hhf2::[HHTS-HHFS]- ura3Δ* H3K37A *set2∆::KanMX* *rtg1∆::HphNT1* | This Study |
| YNL1200 | MATa *his3Δ200 leu2Δ0 lys2Δ0 trp1Δ63 ura3Δ0met15Δ0 can1::MFA1pr-HIS3 hht1-hhf1::NatMX4 hht2-hhf2::[HHTS-HHFS]- ura3Δ* H3WT *hho1∆::HphNT1* | This Study |
| YNL1202 | MATa *his3Δ200 leu2Δ0 lys2Δ0 trp1Δ63 ura3Δ0met15Δ0 can1::MFA1pr-HIS3 hht1-hhf1::NatMX4 hht2-hhf2::[HHTS-HHFS]- ura3Δ* H3K37A *hho1∆::HphNT1* | This Study |
| YNL1283 | MATa *his3Δ200 leu2Δ0 lys2Δ0 trp1Δ63 ura3Δ0met15Δ0 can1::MFA1pr-HIS3 hht1-hhf1::NatMX4 hht2-hhf2::[HHTS-HHFS]- ura3Δ* H3WT *tom11∆::HphNT1* | This Study |
| YNL1285 | MATa *his3Δ200 leu2Δ0 lys2Δ0 trp1Δ63 ura3Δ0met15Δ0 can1::MFA1pr-HIS3 hht1-hhf1::NatMX4 hht2-hhf2::[HHTS-HHFS]- ura3Δ* H3K37A *tom1∆::HphNT1* | This Study |
| YNL1204 | MATa *his3Δ200 leu2Δ0 lys2Δ0 trp1Δ63 ura3Δ0met15Δ0 can1::MFA1pr-HIS3 hht1-hhf1::NatMX4 hht2-hhf2::[HHTS-HHFS]- ura3Δ* H3WT *COX4-3XHA::HphNT1* | This Study |
| YNL1206 | MATa *his3Δ200 leu2Δ0 lys2Δ0 trp1Δ63 ura3Δ0met15Δ0 can1::MFA1pr-HIS3 hht1-hhf1::NatMX4 hht2-hhf2::[HHTS-HHFS]- ura3Δ* H3K37A *COX4-3XHA::HphNT1* | This Study |

**Reference**

1.	Baker Brachmann, C., et al., *Designer deletion strains derived from Saccharomyces cerevisiae S288C: a useful set of strains and plasmids for PCR‐mediated gene disruption and other applications.* Yeast, 1998. **14**(2): p. 115-132.

2.	Clark-Adams, C.D., et al., *Changes in histone gene dosage alter transcription in yeast.* Genes & development, 1988. **2**(2): p. 150-159.

3.	DiFiore, J.V., et al., *Unique and shared roles for histone H3K36 methylation states in transcription regulation functions.* Cell Reports, 2020. **31**(10).

4.	Kizer, K.O., et al., *A novel domain in Set2 mediates RNA polymerase II interaction and couples histone H3 K36 methylation with transcript elongation.* Molecular and cellular biology, 2005. **25**(8): p. 3305-3316.

5.	Chen, H., et al., *The histone H3 lysine 56 acetylation pathway is regulated by target of rapamycin (TOR) signaling and functions directly in ribosomal RNA biogenesis.* Nucleic acids research, 2012. **40**(14): p. 6534-6546.

6.	Laferté, A., et al., *The transcriptional activity of RNA polymerase I is a key determinant for the level of all ribosome components.* Genes & development, 2006. **20**(15): p. 2030-2040.
